# Assembly and application of a low-cost high-resolution imaging device for hyphae in soil

**DOI:** 10.1371/journal.pone.0318083

**Published:** 2025-01-24

**Authors:** Holger Schaefer

**Affiliations:** Kansai Research Center, Forestry and Forest Products Research Institute, Kyoto City, Kyoto Prefecture, Japan; Universitat Jaume 1, SPAIN

## Abstract

Soil imaging in the field and laboratory has greatly advanced our understanding of plant root systems. Soil fungi function as important plant symbionts and decomposers of complex organic material in soil environments. For fungal hyphae, however, the application of soil imaging remains scarce, limiting our understanding of hyphal systems in soil. This scarce application is partly due to the challenging development of a soil imaging device for hyphae: technical requirements to resolve fine hyphae (2–5 μm in diameter) are high, while the device cost must be low to facilitate sufficient deployment that can capture the high spatial heterogeneity of hyphal dynamics in soil. This protocol describes the do-it-yourself assembly and application of a low-cost high-resolution imaging device for observing hyphae in soil. The assembly of the open-source imaging device relies on many 3D-printed parts, reducing material costs to ca. 930 USD. The application of the imaging device yields soil profile images with a resolution of up to 0.52 μm px^-1^ (49000 dpi) within an observable volume of 70 × 210 × 1.5 mm. By repeatedly imaging a soil profile using the presented techniques, changes in the amount, distribution, and morphology of hyphae in soil can be observed and quantified.

## Introduction

Soil imaging—the repeated photographing of an exposed soil profile with a camera or scanning system—has become a key technology for ecologists to quantify and understand physiological processes in soil. In previous studies, soil imaging has mostly been applied to capture changes in the amount, distribution, and morphology of plant roots [1]. Major advantages of soil imaging over destructive root sampling are that it enables the repeated *in situ* observation of the same individual root and the simultaneous measurement of root production and mortality [1, 2]. In the field, soil imaging is often performed using specialized devices called “minirhizotrons” [2, 3], where the camera or scanning system is placed in a transparent plastic or glass tube buried in the soil. Alternatively, transparent boxes containing flatbed scanners have been used [4]. In laboratory studies, soil imaging in boxes or pots is conducted with imaging devices that are either buried [5, 6] or placed against the transparent walls of the soil container [7]. The application of soil imaging in both the field and laboratory over many decades has led to great leaps in our understanding of root systems.

Plants coexist with soil fungi: mycorrhizal soil fungi, for instance, are important plant symbionts that provide nutritional benefits in exchange for photosynthates [8, 9], while saprotrophic soil fungi are important decomposers of complex organic material and mediate the mineralization of plant litter [10, 11]. The biomass of soil fungi constitutes a significant fraction of the total microbial biomass in terrestrial soil [11] and is of the same order of magnitude as the root biomass in some ecosystems [12]. Much of the soil fungal biomass consists of fine, elongated filamentous structures called hyphae, which branch into diffuse fans or form linear aggregations such as “rhizomorphs” [13, 14]. In the field, hyphal biomass has mostly been measured destructively through the monthly or yearly placement of hyphal ingrowth bags in soil [15–19]. Soil imaging, while viewed as a promising method for the repeated, nondisruptive *in situ* observation of hyphae [20], has only been applied at a few sites in the United States [21–25]. In laboratory studies, the imaging of hyphae in soil has been slightly more frequent, usually performed through labor-intensive manual investigations of transparent soil containers with a microscope [26, 27]. Overall, however, the application of soil imaging to hyphae remains scarce, limiting our understanding of hyphal systems in soil.

One important reason for the limited application of soil imaging to hyphae is the lack of suitable imaging devices. Imaging hyphae requires a high imaging resolution, since individual hyphae are often only 2–5 μm thick [27, 28]. The imaging resolution of soil imaging devices is usually stated in dots per inch (dpi), i.e., the number of pixels along a horizontal or vertical distance of 25.4 mm on the imaged surface. However, a more intuitive measure is the pixel width (μm px^-1^), i.e., the horizontal or vertical distance on the imaged surface covered by a single pixel. To resolve objects in soil imagery, they should be at least two pixels wide or, when considering factors such as environmental conditions and object orientation, four pixels wide [29–31]. Thus, to resolve a very fine root that is 100 μm in diameter, an imaging resolution of 25–50 μm px^-1^ (500–1000 dpi) suffices, which is already achievable with low-cost document scanners. In contrast, to resolve fine hyphae with diameters of 2 μm, a much higher imaging resolution of 0.5–1 μm px^-1^ (25400–50800 dpi) is required. Even the, to my knowledge, highest imaging resolution currently achieved by a soil imaging device at 4.7 μm px^-1^ (5400 dpi) [24, 31] is, therefore, insufficient to resolve fine hyphae.

To increase the imaging resolution of soil imaging devices, hardware manufacturers have developed increasingly advanced camera and scanning systems. A commonly used design relies on a digital microscope camera (DMC) that magnifies the soil profile up to 100× [21, 25, 32]. Since DMCs have a limited field of view, numerous images must be taken from precisely spaced camera positions and later assembled in software to create a larger image of the soil profile [32]. These advanced camera and scanning systems must be capable of fine movements performed by precisely manufactured parts, which leads to higher development and production costs, and in turn, higher per-unit prices on the market. Soil imaging devices typically cost many thousand USD per unit [33], limiting the number of devices deployable in a study. Hyphal growth dynamics in soil, however, have shown a high spatial heterogeneity in laboratory [34] and field studies [17, 20], demanding low-cost soil imaging devices that can be attained and deployed in large numbers. At the same time, the soil imaging of hyphae should ideally be automated to capture hyphae with short lifespans of only several days [27, 35] without requiring frequent, laborious manual investigations.

The key to enabling the automated high-resolution imaging of hyphae in soil at a low cost may lie in the do-it-yourself assembly of open-source hardware. Since information on the design and assembly process of open-source hardware is published under permissive licenses, anyone can study, reproduce, distribute, and modify the hardware [36]. The do-it-yourself assembly of open-source hardware in research laboratories can considerably reduce the cost of research equipment [37]. Due to its accessibility, high rate of innovation, and low cost, open-source hardware has continually replaced functionally equivalent proprietary research equipment in various scientific fields [37, 38]. In microscopy, several protocols have been published on how to assemble and apply imaging devices for the observation of prepared samples in small static media such as the *OpenSPIM* [39], *OpenFlexure* [40], *Microscopi* [41], and *UC2* microscopes [42]. However, these imaging devices have observable volumes limited to several millimeters on each spatial axis, e.g. 12 × 12 × 4 mm for the *OpenFlexure* microscope [43]. Such small observable volumes are insufficient to capture the changes in hyphal systems across a soil profile.

This protocol describes the do-it-yourself assembly and application of a low-cost, high-resolution imaging device for hyphae in soil, called *Hyphascope*. The design of the imaging device was adopted from the 3D printer i3 MK3S+ (Prusa Research, Prague, Czech Republic), with a DMC (3R-MSUSB601; 3R Solution, Fukuoka, Japan) replacing the filament extruder. After a comprehensive *Materials* section that breaks down material costs and potential retailers, the protocol provides a detailed step-by-step description of how the imaging device is assembled, wired, and supplied with software, building upon the original assembly manuals for the 3D printer i3 MK3S+. Subsequently, the setup of the imaging device in soil and the imaging process are described, including the automated acquisition of DMC images and their registration into a large continuous image of the soil profile. The 3D-printing of device parts takes approximately 32 h, the device assembly approximately 1–2 days, and the installation in soil takes several hours. By repeatedly imaging a soil profile following the protocol, researchers can to observe and quantify changes in the amount, distribution, and morphology of hyphae in soil.

## Materials and methods

The protocol described in this peer-reviewed article is published on protocols.io, https://dx.doi.org/10.17504/protocols.io.bp2l6xo3zlqe/v1, and is included for printing as S1 File with this article. Furthermore, three sets of high-resolution images yielded following the protocol are available from Zenodo at https://doi.org/10.5281/zenodo.10730414. The high-resolution images were acquired between May and October 2023 in a *Quercus serrata* grove at the Kansai Research Center of the Forestry and Forest Products Research Institute (FFPRI) in Kyoto City, Japan (34°56’N, 135°46’E). The grove was located on a Cambisol [44] in flat terrain. Monthly mean air temperature ranged between 18.2 and 30.3°C, and monthly precipitation ranged between 61 and 253 mm [45].

## Expected results

**Fig 1:**
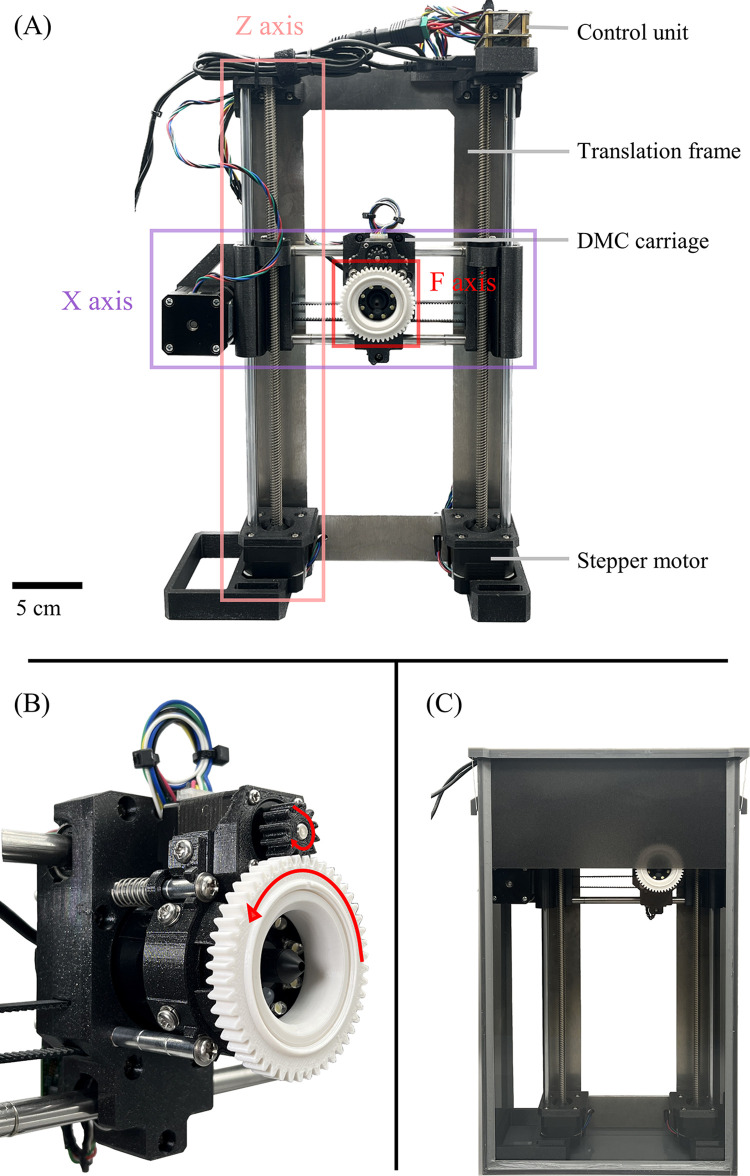
The low-cost high-resolution imaging device for hyphae in soil assembled following the protocol. (A) Labeled photo of the assembled imaging device. The design was adopted from the 3D printer i3 MK3S+ (Prusa Research, Prague, Czech Republic), with a digital microscope camera (DMC) replacing the filament extruder. (B) Close-up of the DMC carriage which has a focusing mechanism that moves the DMC perpendicular to the soil profile by rotating a threaded attachment (in white). (C) Photo of the imaging device inside the observation box.

The assembled imaging device moves a DMC (600× magnification) horizontally (X axis), vertically (Z axis), and perpendicular to the soil profile (F axis; Fig 1A). Horizontal movement is facilitated by a timing belt rotated with a stepper motor at the side of the device, and vertical movement is enabled by two threaded rods rotated with stepper motors at the bottom of the device. The X and Z axis designs were adopted from the 3D printer i3 MK3S+ with only minor modifications. Movement perpendicular to the soil profile is enabled by an original focusing mechanism integrated into the DMC carriage (Fig 1B). The DMC is mounted on four parallel rods and maintained, with a compression spring, lightly pushed against the inside of the observation box buried in soil (Fig 1C). By rotating a threaded attachment with a stepper motor the DMC is pushed away from or moved towards the soil profile. Stepper motors are operated by a small single-board computer attached to the top of the device. The DMC movement along the three axes increases the observable volume from the field of view (0.83 × 0.62 mm) and depth of field (0.025 mm) of the DMC to 70 mm (X axis) × 210 mm (Z axis) × 1.5 mm (F axis). Material costs for assembling the imaging device were approximately 930 USD (see the *Materials* section in the protocol).

**Fig 2:**
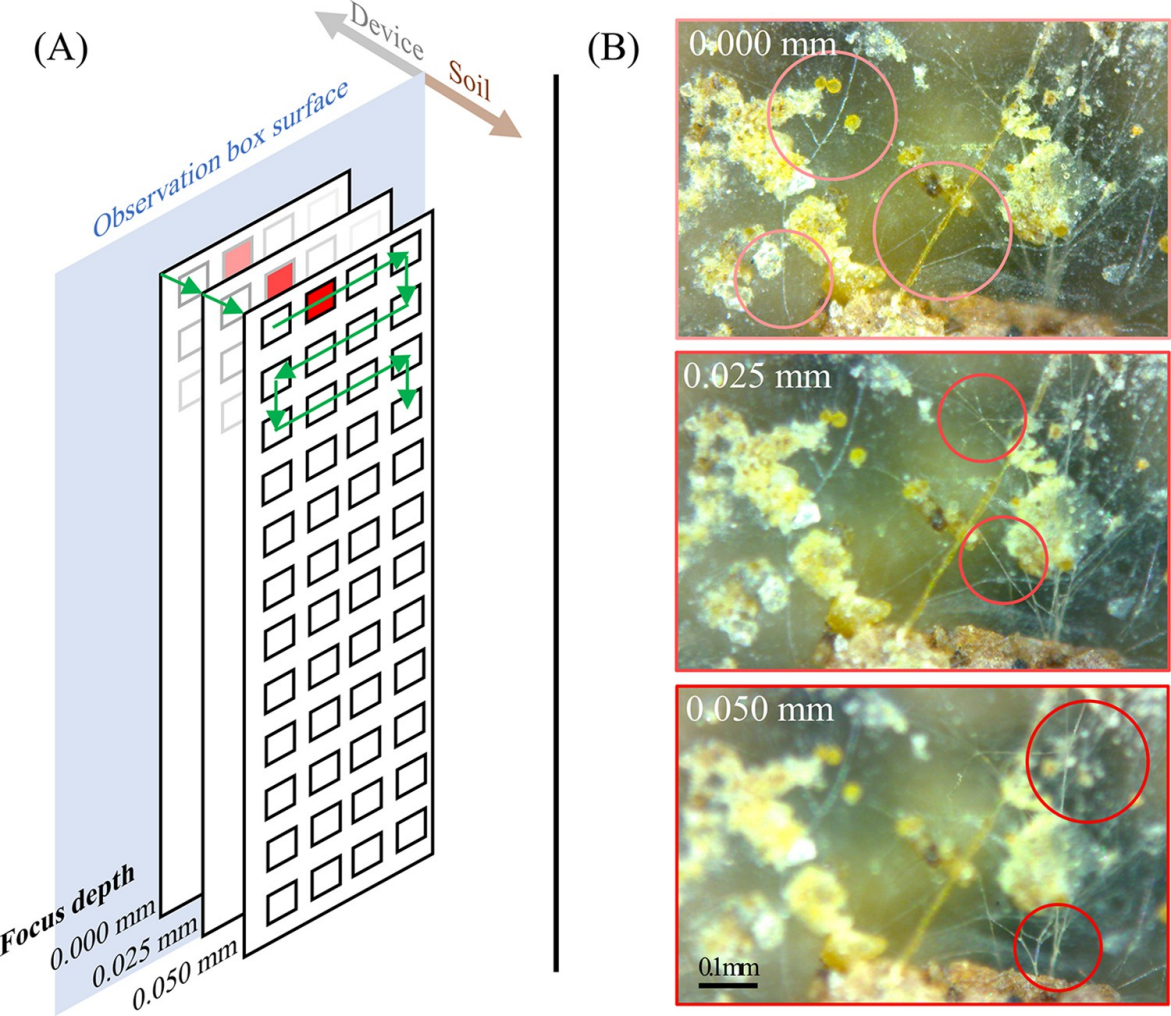
Automated soil imaging process initiated by the user following the protocol. (A) Diagram depicting the DMC movement (green arrows) during soil imaging: images are captured at all XZ positions before focusing deeper into the soil profile. (B) Soil profile images yielded at three different focus depths (white font) at an imaging resolution of 0.65 μm px^-1^ (39200 dpi). Red circles indicate hyphae in focus. Images were cropped, and originals are included in the S1 Dataset.

**Fig 3:**
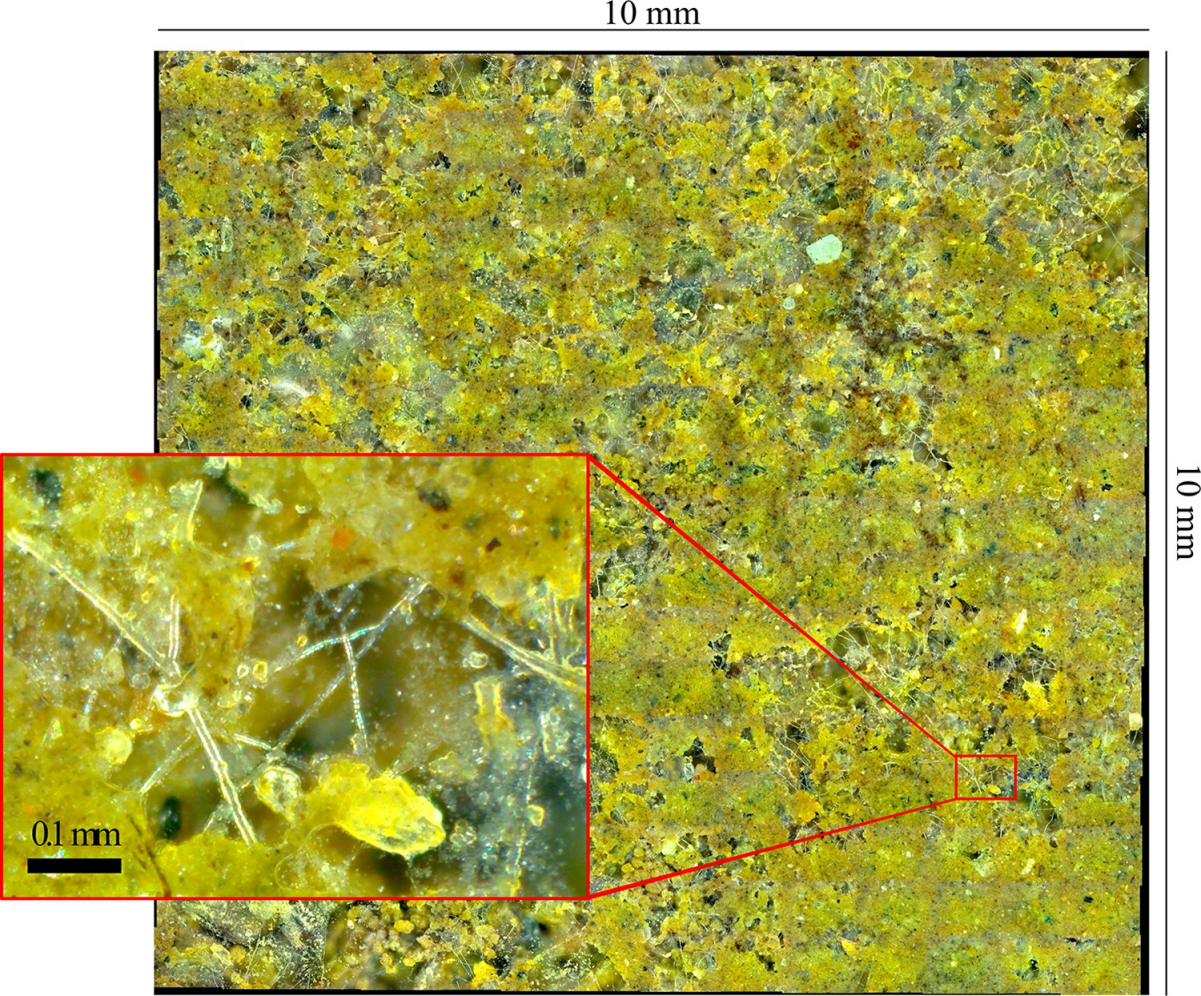
High-resolution soil profile image yielded following the protocol. Imaging was performed at 0.65 μm px^-1^ (39200 dpi) in a *Quercus serrata* grove in May 2023 and aligned with the *Grid/Collection stitching* plugin of *Fiji* [46]. Original images are available from the data repository Zenodo (see the *Materials & methods* section).

The application of the imaging device yields soil profile images at imaging resolutions of 1.30 μm px^-1^ (19600 dpi), 0.65 μm px^-1^ (39200 dpi), or 0.52 μm px^-1^ (49000 dpi), depending on the DMC settings. Soil imaging is automated, following the preferences set by the user in a short, easy-to-understand script file. The imaging is done in focus depth layers (Fig 2A), yielding sharp images of hyphae at different distances from the observation box (Fig 2B). Neighboring DMC images are taken with small overlaps (0.16 mm horizontally, 0.08 mm vertically) to enable the registration into a large continuous image of the soil profile at each focus depth (Fig 3). Three sets of high-resolution soil profile images yielded with the imaging device are provided (see the *Materials & methods* section). Imaging of a user-set volume of 10 × 10 × 0.05 mm results in 14 × 18 × 3 images and takes approximately 11, 22, and 40 min at imaging resolutions of 1.30, 0.65, and 0.52 μm px^-1^, respectively. Researchers are advised to divide the imaging of a soil profile into multiple user-set volumes that are approximately 10 mm wide for easier image registration and analysis.

The presented protocol is limited in scope to the assembly and application of the imaging device. Depending on the target soil or experimental design, modifications to the presented hard- and software may be necessary. Researchers are encouraged to make self-guided modifications to the open-source device using the provided resources which are shared under permissive licenses. Furthermore, the protocol does not address the analysis of soil profile images and the major challenges it presents to researchers, such as the large amount of time required for manual image analysis [20, 24, 47, 48], the difficulty to differentiate between different functional types or species of fungi [20, 23], and the conversion of pixel areas to biomass per soil volume or land area [1, 20]. Yet, addressing these challenges first requires sufficient, high-quality image data from a variety of soil environments. The presented protocol is believed to be an important step toward generating such image data and further advancing our understanding of soil fungi.

## Supporting information

S1 File Assembly and application of an imaging device for hyphae in soil. The protocol is also available on protocols.io.
(PDF)

S1 Dataset Set of soil profile images acquired at focus depths of 0, 0.025, and 0.05 mm. Original images in the JPG format taken at an imaging resolution of 0.65 μm px^-1^ (39200 dpi).
(ZIP)
