# Supplementary material for: Assembly and application of a low-cost high-resolution imaging device for hyphae in soil
Source: PLoS One. 2025 Jan 24;20(1):e0318083. doi: 10.1371/journal.pone.0318083 (PMC11760638; doi:10.1371/journal.pone.0318083)
Supplement: S1 File — The protocol is also available on protocols.io. (PDF) [file pone.0318083.s001.pdf]

Dec 27, 2024

Hyphascope: Do-it-yourself assembly and application of an imaging device for hyphae in soil

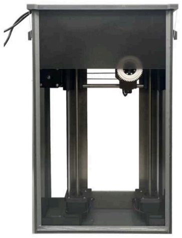

DOI
dx.doi.org/10.17504/protocols.io.bp2l6xo3zlqe/v1

Holger Schaefer[1]

[1]Kansai Research Center, Forestry and Forest Products Research Institute, Kyoto City, Kyoto Prefecture, Japan. Current address: Department of Forest Soils, Forestry and Forest Products Research Institute, Tsukuba City, Ibaraki Prefecture, Japan

Holger Schaefer
Forestry and Forest Products Research Institute (FFPRI), Jap...

OPEN ACCESS

DOI: dx.doi.org/10.17504/protocols.io.bp2l6xo3zlqe/v1

Protocol Citation: Holger Schaefer 2024. Hyphascope: Do-it-yourself assembly and application of an imaging device for hyphae in soil. **protocols.io https://dx.doi.org/10.17504/protocols.io.bp2l6xo3zlqe/v1**

License: This is an open access protocol distributed under the terms of the **Creative Commons Attribution License**, which permits unrestricted use, distribution, and reproduction in any medium, provided the original author and source are credited

Protocol status: Working
We use this protocol and it's working

Created: March 11, 2024

Last Modified: December 27, 2024

Protocol Integer ID: 96472

Keywords: mycorrhizal fungi, saprotrophic fungi, mycelium, hyphae, soil imaging, imaging device, minirhizotron, open-source, microscope, soil profile, soil microbes

Funders Acknowledgements:
Japan Society for the Promotion of Science
Grant ID: JP22K20595

Abstract

This protocol describes the do-it-yourself assembly and application of a low-cost high-resolution imaging device for hyphae in soil, called *Hyphascope*. The imaging device's design was adopted from a 3D printer, with a digital microscope camera (DMC) replacing the filament extruder. The application of the imaging device yields soil profile images at an imaging resolution of up to 0.52 μm px^{-1} (49000 dpi) within an observable volume of 70 × 210 × 1.5 mm. Repeated imaging of a soil profile following the protocol enables researchers to observe and quantify changes in the amount, distribution, and morphology of hyphae in soil.

Image Attribution

All images in this protocol were taken by the author.

Guidelines

- Experience in 3D printing is advantageous but not required.
- Attain all tools and materials (*Materials Section*) and prepare all device parts (*Section 1*) before starting to assemble the device.
- It is recommended to have the aluminum and acrylic parts order-cut by an online service of choice. Self-cutting requires additional equipment (see *Section 1*).
- The 3D printing of all parts takes about 32 hours, device assembly about 1 -2 days, and installation several hours.
- Step durations depend on the skill level of the protocol user and are not specified.
- Read all the substeps of a step, before executing the described procedures.

Materials

Tools

- Computer (Windows 10 or 11)
- 3D printer (e.g. *Prusa i3 MK4S* available from ***prusa3d.com***)
- Screwdriver for M2 screws (tip width 1 mm for Phillips or JIS)
- Screwdriver for M3 screws (tip width 2 mm for Phillips or JIS)
- Screwdriver for slotted screws (tip width 1.5 - 2 mm)
- Permanent marker
- Wrench (width across flats 5.5 mm)
- Hex key (width across flats 2 mm)
- Needle-nose pliers
- Wire flush cutter
- Fine plastic saw
- Box cutter
- Dust blower
- Soldering iron
- Wire stripper
- Measure
- MicroSD card reader
- Wi-Fi network with internet access
- *(observation box)* Silicon sealant extruder
- *(observation box)* Silicon sealant smoothing tool
- *(observation box)* Application device for solvent-based adhesive
- *(device installation)* Spade
- *(device installation)* Spirit level
- *(device installation)* Sturdy plastic bags

Design files

- STL files of 3D-printed and cut parts (available from the data repository ***Zenovo***)

Electronic parts 540 USD *(sum of listed prices as of 2024/12/23)*

- Z-axis stepper motor, right (*Stepper motor Z-axis Right* by Prusa Research; available from **prusa3d.com**), 1 pc.
- Z-axis stepper motor, left (*Stepper motor Z-axis Left* by Prusa Research, cable extended to 55 cm; available from **prusa3d.com**), 1 pc.
- X-axis stepper motor (model 17HM15-0904S by Oyostepper; available from **oyostepper.com**), 1 pc.
- F-axis stepper motor (model 8HS12-0506S by Stepperonline; available from **omc-stepperonline.com**), 1 pc.
- Digital microscope camera (DMC; model 3R-MSUSB601 by Three R Solution; available from **qtetech.com**), 1 pc.
- *Raspberry Pi Zero 2 W* (Raspberry Pi Foundation, with pre-soldered GPIO header; available from **pishop.us**), 1 pc.
- *DC & Stepper Motor Bonnets* (product 4280 by Adafruit Industries; available from **adafruit.com**), 2 pcs.
- microSD card (model *512GB Extreme microSDXC* by SanDisk; available from **amazon.com**), 1 pc.
- Raspberry Pi power supply (model UU318-0530 by KSY; alternative model available from **pishop.us**), 1 pc.
- Stepper motor power supply (model UC08U-SB by SoulBay; available from **amazon.com**), 1 pc.

- GPIO stacking headers (product 2223 by Adafruit Industries; available from **adafruit.com**), 2 pcs.
- USB adapter (product 2910 by Adafruit Industries; available from **adafruit.com**), 1 pc.

Mechanical parts 110 USD *(sum of listed prices as of 2024/12/23)*

- Stainless-steel rods, length 320 mm (*Smooth rod Z-axis* by Prusa Research; available from **prusa3d.com**), 2 pcs.
- Stainless-steel rods, length 187 mm (*Smooth rod Z-axis* by Prusa Research, cut to length; available from **prusa3d.com**), 2 pcs.
- Linear bearings (*Linear bearing LM8UU* by Prusa Research; available from **prusa3d.com**), 7 pcs.
- Bearing housing (*Bearing housing 623h* by Prusa Research; available from **prusa3d.com**), 1 pc.
- Timing pulley (*Timing pulley T16-2GT* by Prusa Research; available from **prusa3d.com**), 1 pc.
- Timing belt (model 760-2GT-6 by Gates Unitta Asia; available from **misumi-ec.com**), 1 pc.
- Compression spring (model T-070-01 by Shinsei Hatsujo; available from **misumi-ec.com**), 1 pc.
- *(observation box)* Protective vent (model PMF-12HAB by Takachi; available from **misumi-ec.com**), 1 pc.

3D printing filament 60 USD *(sum of listed prices as of 2024/12/23)*

- PETG filament, dark (*Prusament PETG Galaxy Black* by Prusa Research; available from **prusa3d.com**), ≥ 300 g
- PETG filament, white (*Prusament PETG Signal White* by Prusa Research; available from **prusa3d.com**), ≥ 20 g

Screws, nuts, and washers 65 USD (*assuming 5 USD per item)*

Available from hardware stores or **mcmaster.com**. All stainless-steel.

- M2×5 mm screw (pan-head, thread pitch 0.5 mm), 4 pcs.
- M2.5×6 mm screw, 4 pcs.
- M3×7 mm screw, 9 pcs.
- M3×10 mm screw, 18 pcs.
- M3×18 mm screw, 8 pcs.
- M3×20 mm screw, 7 pcs.
- M3×65 mm screw, 4 pcs.
- M2.5 nuts, 4 pcs.
- M3 nuts, 33 pcs.
- M3 nyloc nut, 1 pc.
- M3 square nuts (width 5.4 mm, height 2.1 mm), 9 pcs.
- M3 washers, 16 pcs.
- M3 split washers, 4 pcs.

Spacers 15 USD (*assuming 5 USD per item)*

Available from local hardware stores or **mcmaster.com**.

- M2.5×12 mm hexagonal spacer (brass, male to female, screw length 6 mm), 4 pcs.
- M2.5×15 mm hexagonal spacer (brass, male to female, screw length 6 mm), 4 pcs.
- M3×10 mm round spacer (aluminum, diameter 5 mm, threaded through-hole), 16 pcs.

Sheet materials 80 USD (*assuming 20 USD per item)*

Available from local hardware stores or **amazon.com**.

- Aluminum sheet (alloy A6061, 400 × 250 mm, 3 mm or 0.125 inch thick), 1 pc. (only if self-cut, see *Section 1*)
- *(observation box)* Acrylic sheet (cast, opaque/dark, 600 × 450 mm, 5 mm thick), 1 pc. (only if self-cut, see *Section 1*)
- *(observation box)* Glass sheets (transparent, 420 × 252 mm, 3 mm or 0.125 inch thick), 2 pcs.

Consumables 60 USD (*assuming 5 USD per item)*

Available from local hardware stores or **amazon.com**.

- Cable ties (length 100 mm, width 2.5 mm, black), 18 pcs.
- Masking tape
- Insulation tape
- Clear tape
- Solder
- Paper towels
- Wire AWG22, black (length ≥ 10 cm)
- Wire AWG22, red (length ≥ 10 cm)
- *(observation box)* Silicone sealant (type 8060 in gray by Cemedine, Tokyo, Japan)
- *(observation box)* Solvent-based adhesive for acrylic (type dichloromethane by Acrysunday, Tokyo, Japan)
- *(observation box)* String
- *(observation box)* Opaque tape or foil for glass sheets

Safety warnings

The assembly and installation of the imaging device involves the risk of injury from e.g. mishandling sharp tools or erroneous wiring. The protocol user is advised to carry out the described procedures with care and complement steps that are not immediately understood with related online resources. No responsibility is taken by the author for injury or damages incurred from the use of the protocol.

Before start

- Prepare a clean and well-lit workspace.
- Keep all tools and materials close to the workspace while assembling the imaging device.

Section 1: Preparation of the device parts

1 **Gather tools and materials.**

Tools:

- Computer
- 3D printer
- Slotted screw driver
- Needle-nose pliers
- Hex key

Materials:

- STL files of 3D-printed and cut parts
- PETG printing filament, dark
- PETG printing filament, white
- Aluminum sheet *(optional, only if self-cut)*
- Acrylic sheet *(optional, only if self-cut)*

2 **Prepare the G-code for 3D printing.**

2.1 Download, install, and launch a 3D slicer software such as **PrusaSlicer** (Prusa Research; version 2.5.2 used here).

2.2 Import the stereolithography (STL) files of the 3D-printed parts into the 3D slicer software. Several parts may be printed at the same time (Fig 1.1). However, keep the parts attaching to the front of the digital microscope camera (DMC), *dmc-attachment* and *dmc-attachment-gear*, separate from all other 3D-printed parts, since the former are printed in white and the latter in any dark color.

Note

The parts *dmc-attachment* and *dmc-attachment-gear* are printed in white for better reflection of the DMC's LED light during imaging. All other parts are printed in a dark color to reduce the light that is reflected on them and, thus, limit the exposure of roots and hyphae to reflected light outside of imaging sessions.

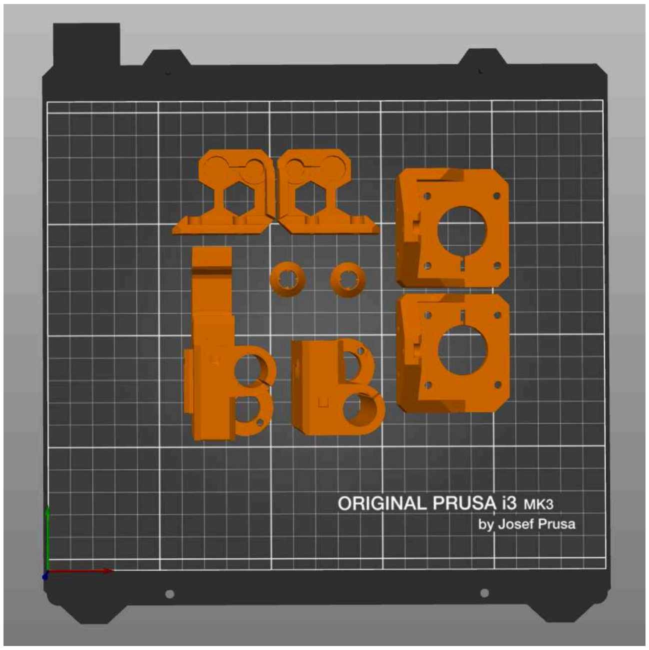

Fig 1.1. X- and Z-axis parts imported into *PrusaSlicer*.

2.3 Set the infill to 20% and the layer height to 0.2 mm (Fig 1.2). A nozzle size of 0.4 mm is recommended.

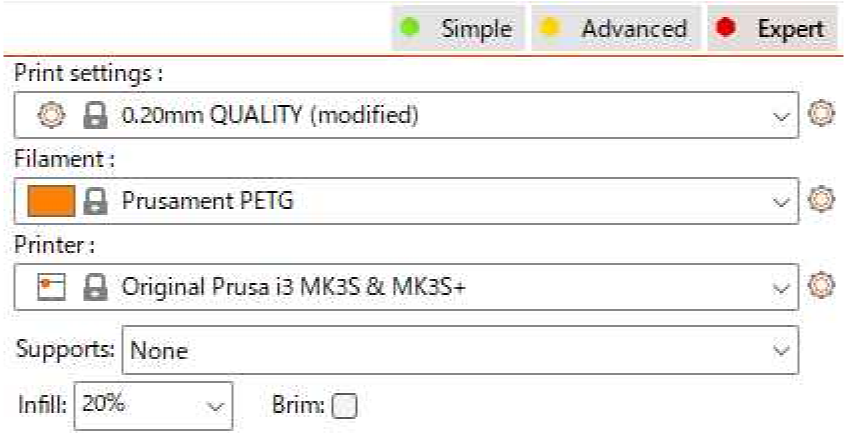

Fig 1.2. Print settings in *PrusaSlicer*.

2.4 For the parts *dmc-carriage-front*, *dmc-carriage-back*, and *dmc-holder-back*, supports from the heatbed need to be added in the print settings (Fig 1.2). In *PrusaSlicer* support locations are set automatically (Fig 1.3).

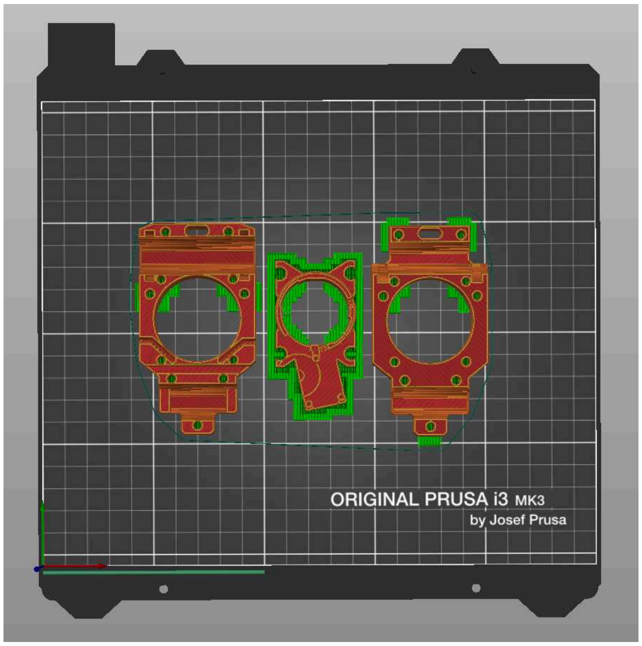

Fig 1.3. Supports from the heatbed automatically set by *PrusaSlicer.*

2.5 For the part *dmc-holder-front*, supports should be added to the elevated head (Fig 1.4).

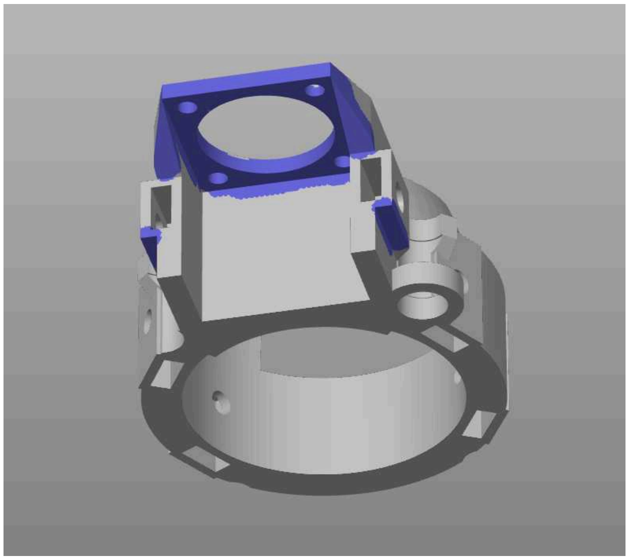

Fig 1.4. Locations for supports (in blue) set with the *Paint-on supports* feature in *PrusaSlicer*.

2.6 Export the G-code for all 3D prints.

3 **Print the device parts.**
For further information on the 3D printing process, consult the PDF file ***HowToPrintParts*** by Prusa Research or other guides.

3.1 To gain a cleaner finish of the 3D-printed parts, all PETG filament may be dried in an oven before use. A clean finish is especially important for the fine threads of the parts printed in white, *dmc-attachment* and *dmc-attachment-gear*. See **this guide** for details on filament drying.

3.2 Print parts from the generated G-code.
Approximate printing time and filament use (g):

1d 7h 20m

Frame parts: 10:00:00 105 g

X-axis parts: 08:30:00 85 g

Z-axis parts: 05:00:00 50 g

F-axis/DMC parts: 07:00:00 60 g

White parts: 00:50:00 8 g

3.3 Remove the supports from the parts *dmc-carriage-front* and *dmc-carriage-back* with a hex key (Fig 1.5). Remove the supports from the part *dmc-holder-front* using needle-nose pliers.

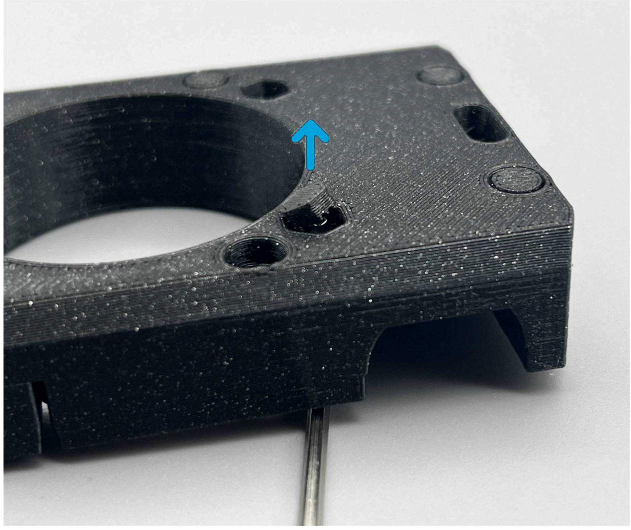

Fig 1.5. Support removal from the part *dmc-carriage-front*

3.4 Check, if the threads of the two white parts of the DMC attachment have a clean finish (Fig 1.6) and test, if the part *dmc-attachment-gear* can be screwed on the other part easily. If not, use the slotted screwdriver or another thin tool to remove impurities in the threads, or reprint parts. You may also test a version of the part *dmc-attachment-gear* with a 0.1 mm-larger inner diameter (see *Materials*).

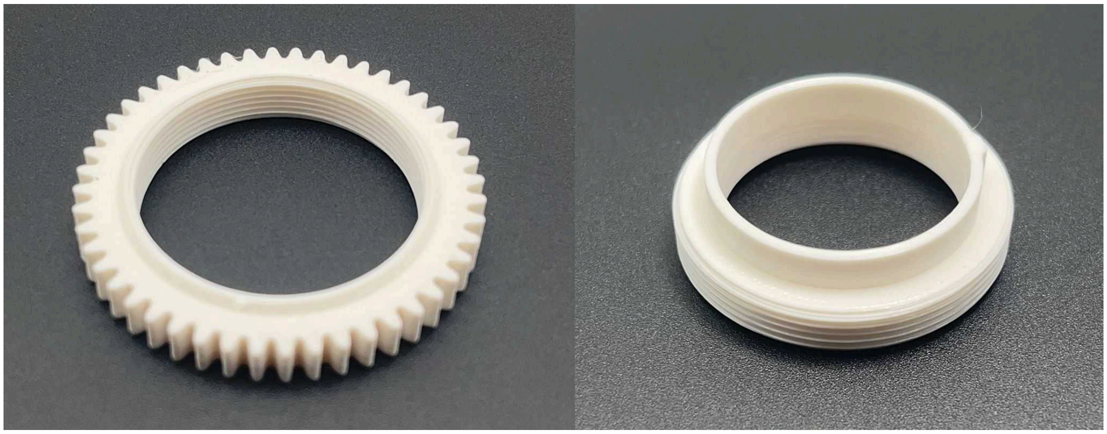

Fig 1.6. Cleanly printed threads on the parts of the DMC attachment.

4 **Prepare the cut parts.**

4.1 Have the part *frame* order-cut from 3 mm-thick aluminum sheet.

4.2 Have the parts starting with *box* order-cut from 5 mm-thick, dark and opaque cast acrylic sheet. Part *box-hook* must be cut four times.

4.3 *(optional)* Aluminum and acrylic parts may be self-cut with a computer numerical control (CNC) router.
Here, the aluminum part was cut using a *Shapeoko 3* (XL size; Carbide 3D) equipped with a *Carbide Compact Router* (Carbide 3D), a touch probe (*BitZero V2*, Carbide 3D), a bit setter (*Shapeoko BitSetter*, Carbide 3D), and a single-flute, flat-nose endmill with a shaft diameter of 3.175 mm (*#274Z*; Carbide 3D). The G-code for the cut was produced with the workbench *Path* of the software *FreeCAD* (version 0.2). Horizontal and vertical feeds were set to 8 mm s^{-1} and 4 mm s^{-1}, respectively. Spindle speed was set to 10000 rpm. No coolant was used. Edges were filed smooth.
The acrylic parts were cut using the same CNC router and equipment. Horizontal and vertical feeds were set to 18 mm s^{-1} and 6 mm s^{-1}, respectively. Spindle speed was set to 12000 rpm. No coolant was used.

Section 2: Assembly of the X-axis

5 **Gather tools and materials.**
Tools:
- Screwdriver (M3)
- Permanent marker

Materials (quantity):

- Part *x-end-motor-mod* (1)
- Part *x-end-idler-mod* (1)
- X-axis stepper motor (1)
- Stainless-steel rods, 187 mm (2)
- Linear bearings (7)
- Timing pulley (1)
- Bearing housing (1)
- M3×18 mm screws (4)
- M3 nyloc nut (1)
- Paper towels

6 **Assemble the X-axis.**

Follow steps 4 and 6 - 12 in the section ***3. X-axis assembly*** of the manual *Original Prusa i3 MK3S+ kit assembly v3.26*. Note the following changes to the original manual:

- Step 4: The rod mounts (*x-end-motor-mod* and *x-end-idler-mod*) are 20 mm higher.
- Steps 4, 6, 12: The screws may be of any type such as hex-, Phillips-, or JIS-head.
- Step 5: (This step is skipped) No tensioner is added to the part *x-end-motor*.
- Steps 7, 9: 187 mm-long stainless-steel rods are used.
- Step 12: The X-axis stepper motor is placed on the front of the part *x-end-motor-mod* with its wires pointing upwards.

The assembled X-axis should resemble the one in Figs 2.1 and 2.2.

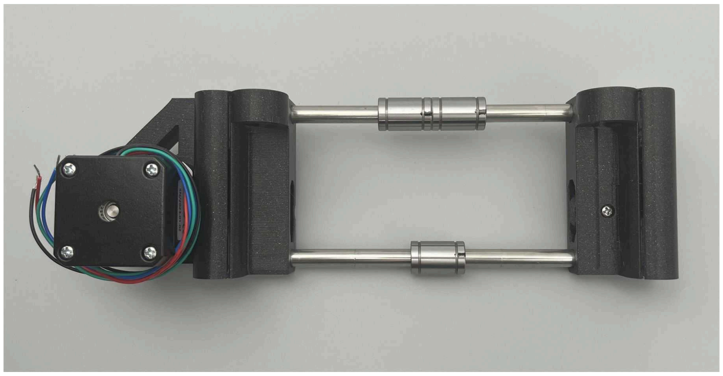

Fig 2.1. Front of the assembled X-axis.

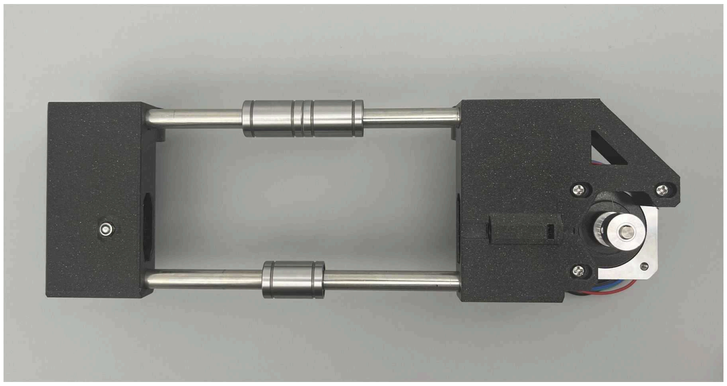

Fig 2.2. Back of the assembled X-axis.

Section 3: Assembly of the Z-axis

7 **Gather tools and materials.**

Tools:

- Screwdriver (M3)
- Wrench

Materials (quantity):

- Part *frame-foot-left* (1)
- Part *frame-foot-right* (1)
- Parts *frame-foot-inserts* (1 left, 1 right)
- Parts *z-axis-bottom* (1 left, 1 right)
- Parts *z-axis-top-mod* (1 left, 1 right)
- Parts *z-screw-cover* (1 left, 1 right)
- Aluminum frame (1)
- Z-axis stepper motor, right (1)
- Z-axis stepper motor, left (1)
- Stainless-steel rods, 320 mm (2)
- M3×7 mm screws (2)
- M3×10 mm screws (18)
- M3×18 mm screws (4)
- M3 nuts (14)
- M3 square nuts (2)
- Clear tape

8 **Insert the aluminum frame into the parts *frame-foot-left* and *frame-foot-right* for easier assembly of the Z-axis (Fig 3.1).**

Note

If the sheet to produce the aluminum frame was 3 mm (rather than 0.125 inch) thick, use 2 - 3 layers of clear tape to thicken the bottom corners of the aluminum frame, so that they fit tightly into the slots of the feet.

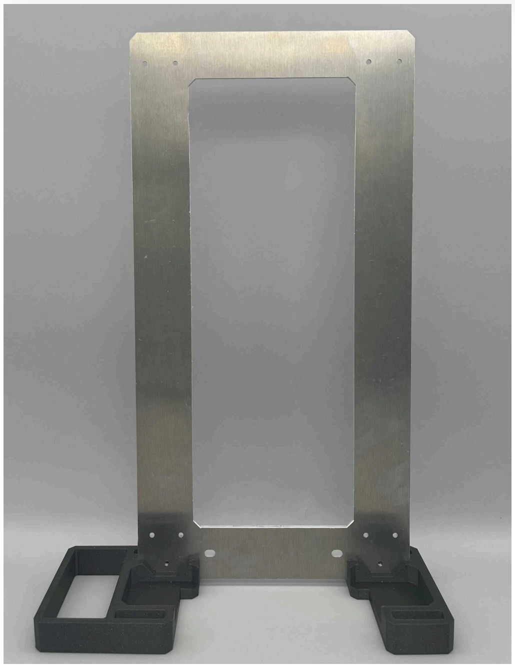

Fig 3.1. Front view of the aluminum frame inserted into the 3D-printed feet.

9 **Assemble the Z-axis.**

Follow all steps 2 - 9 in the section ***4. Z-axis assembly*** of the manual *Original Prusa i3 MK3S+ kit assembly v3.26*. Note the following changes to the original manual:

- Steps 2, 4 - 6, 8, 9: The screws may be of any type such as hex-head, Phillips-head, or JIS-head.
- Steps 2, 8, 9: The aluminum frame is 3 mm thick. Since its holes are not threaded, all screws going through the frame are fastened with an M3 nut from the back of the frame. Hold the M3 nuts with the wrench while fastening.
- Step 4: The cable lengths of both Z-axis stepper motors should be about 55 cm. The Z-axis stepper motors are oriented so that their cables point towards each other. For now the cables are not put through the frame.

The assembled Z-axis should resemble the one in Fig 3.2.

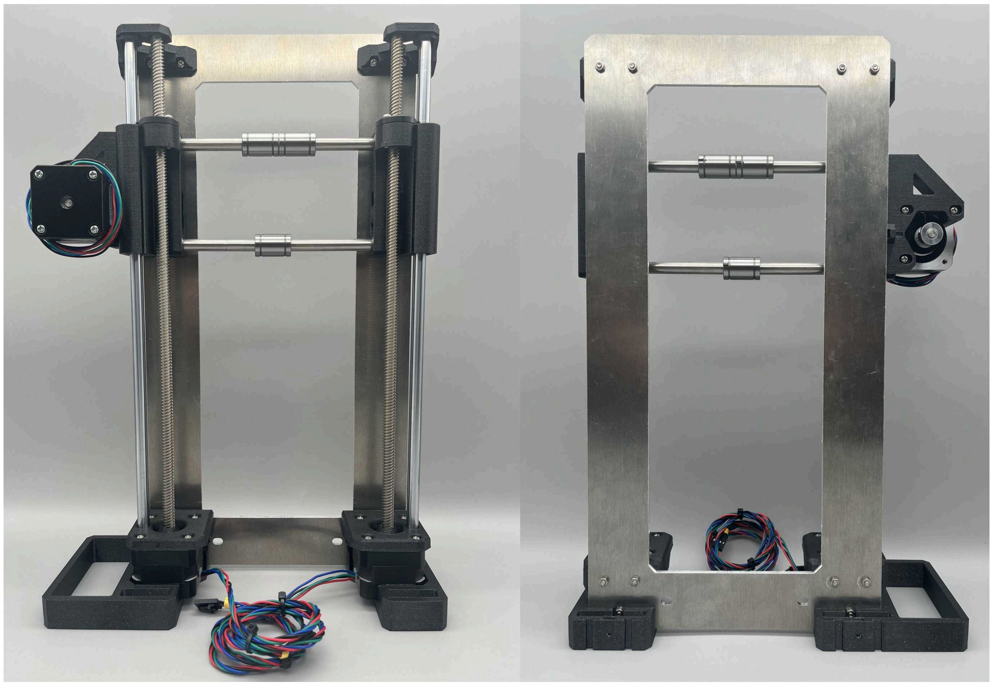

Fig 3.2. Front and back view of the assembled Z-axis

10 **Lock the aluminum frame to the 3D-printed feet.**

10.1 Add an M3 square nut to each of the openings in the back of the feet (Fig 3.3). Push them all the way down with the hex key.

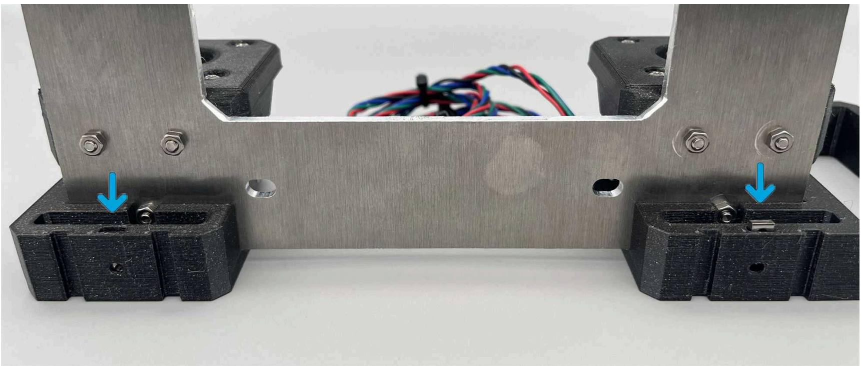

Fig 3.3. Feet with M3 square nuts added.

10.2 Push the parts *frame-foot-inserts* into the feet, add a M3×7 mm screw to each of the inserts, and fasten (Fig 3.4).

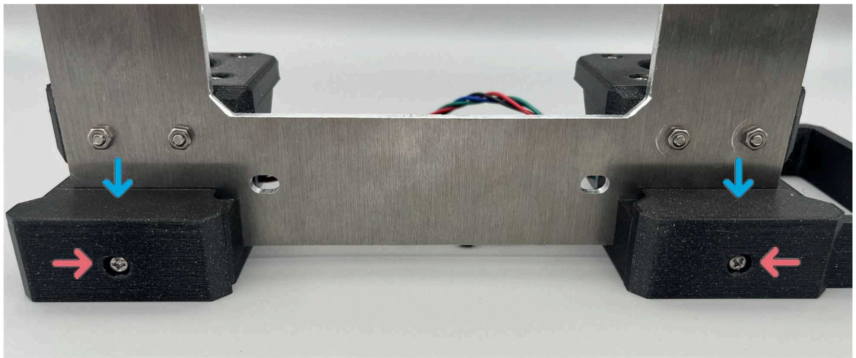

Fig 3.4. Feet with inserts pushed in (blue arrows) and fastened (red arrows)

Section 4: Assembly of the DMC carriage

11 **Gather tools and materials.**

Tools:

- Screwdriver (M3)
- Wire flush cutter
- Needle-nose pliers
- Hex key
- Wrench

Materials (quantity):

- Part *dmc-carriage-front* (1)
- Part *dmc-carriage-back* (1)
- Timing belt (1)
- Cable ties (2)
- M3×20 mm screws (7)
- M3×65 mm screws (4)
- M3 nuts (15)

12 **Attach the front of the DMC carriage to the X-axis.**

Follow steps 49 and 52 - 59 in the section ***5. E-axis assembly*** of the manual *Original Prusa i3 MK3S+ kit assembly v3.26*. Note the following changes to the original manual:

- Step 49: The part *dmc-carriage-front* is used instead of the X-carriage. The inserts for the cable ties (zip ties) are at the same locations.
- Step 52: The timing belt may be as short as 500 mm. The inserts for the timing belt are shorter.
- Step 54, 55: Since the X-axis stepper motor is attached to the front, it does not move freely towards the carriage. Sufficient belt tension can still be achieved without rotating the motor. Simply pull at the end of the timing belt and insert it into the part *dmc-carriage-front*.
- Step 59: There is no tightening screw for the X-axis stepper motor. But the belt can be sufficiently tensioned by refastening the motor on the part *x-end-motor-mod* while pushing it away from the carriage.

The X-axis should resemble the one in Fig 4.1.

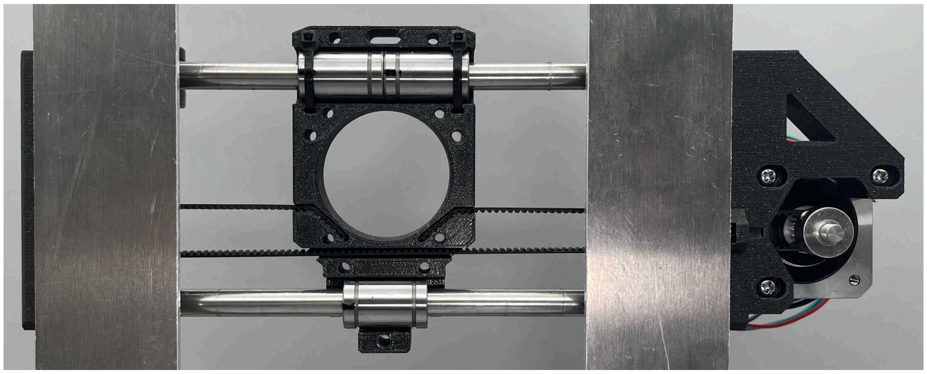

Fig 4.1. X-axis with the part *dmc-carriage-front* attached.

13 **Align the front and back part of the DMC carriage.**

13.1 Add M3 nuts to the hexagonal holes of the parts *dmc-carriage-front* and *dmc-carriage-back* (Fig 4.2). Each M3 nut has to be pulled all the way into the hole. To do that, insert a M3×20 mm screw into the M3 nut from the opposite side of the printed part and fasten the screw. Once the M3 nut is pulled all the way in, remove the screw.

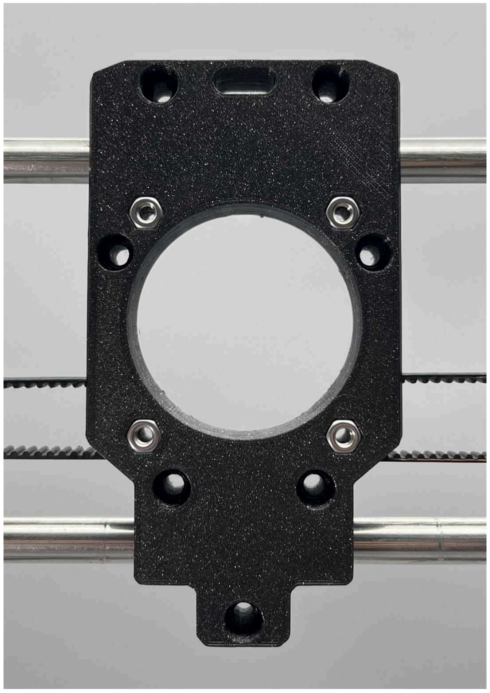

Fig 4.2. Part *dmc-carriage-front* with M3 nuts inserted.

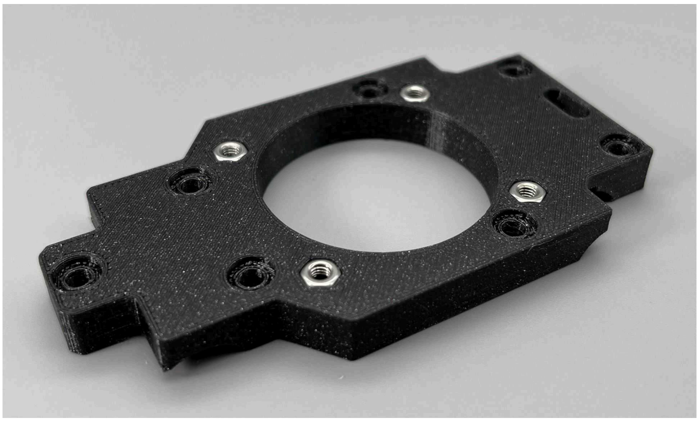

Fig 4.3. Part *dmc-carriage-back* with M3 nuts inserted.

13.2 While holding the part *dmc-carriage-back* tightly against the backside of the part *dmc-carriage-front*, insert four M3×65 mm screws into the front of the part *dmc-carriage-front* (Fig 4.4). In this step, the screws are only used to align the two parts. They are not fastened.

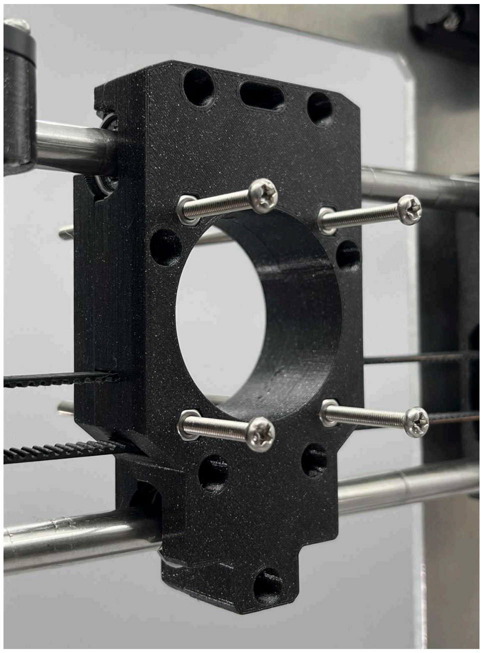

Fig 4.4. Front of the DMC carriage with screws added for alignment.

14 **Join the front and back part of the DMC carriage.**

14.1 Insert three M3×20 mm screws into the top and bottom holes of the part *dmc-carriage-front* (Fig 4.5). On the backside of the DMC carriage, add M3 nuts to the screws and hold them with the wrench to fasten tightly.

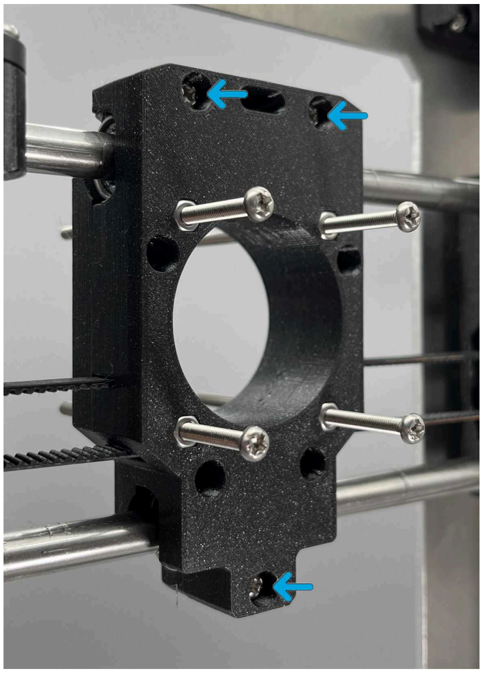

Fig 4.5. The DMC carriage with the first three screws fastened.

14.2 Now that the two parts of the DMC carriage are tightly joined, remove the four M3×65 mm screws used for alignment. Then, add M3×20 mm screws to the remaining four round holes, and tighten them with M3 nuts in the back of the DMC carriage (Figs 4.6, 4.7).

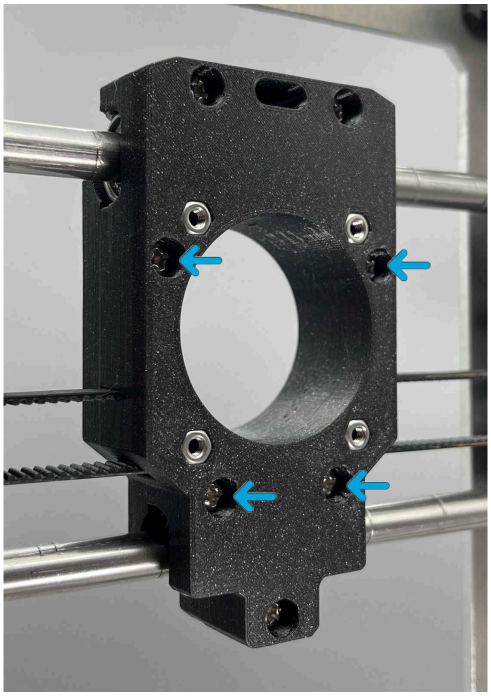

Fig 4.6. Front of the DMC carriage with four more screws fastened.

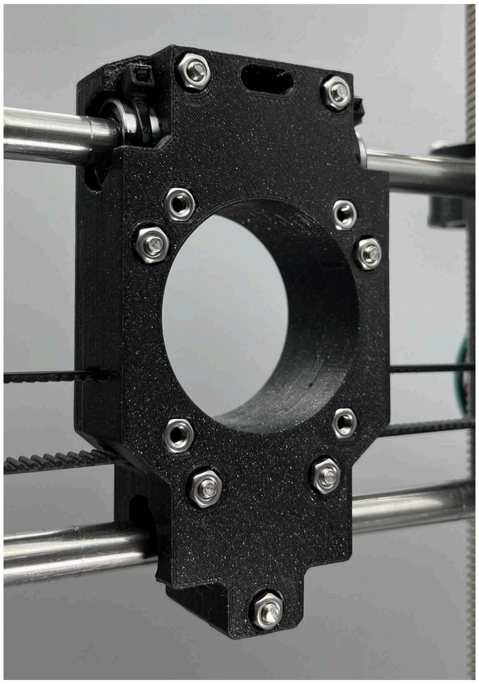

Fig 4.7. Back of the DMC carriage with all screws fastened.

14.3 If any of the M3 nuts in the hexagonal holes of the DMC carriage have been pushed out, push them back in.

Section 5: Mounting of the DMC

15 **Gather tools and materials.**

Tools:

- Screwdriver (M2)
- Screwdriver (M3)
- Needle-nose pliers
- Wire flush cutter
- Wrench
- Hex key
- Fine plastic saw
- Dust blower
- Box cutter

Materials (quantity):

- Part *dmc-holder-front* (1)
- Part *dmc-holder-back* (1)
- Part *dmc-attachment* (1)
- Part *dmc-attachment-gear* (1)
- Part *f-axis-motor-gear* (1)
- Part *f-axis-tighteners* (1 left, 1 right)

- Part *f-axis-spring-end* (1)
- DMC (1)
- F-axis stepper motor (1)
- Compression spring (1)
- Clear tape
- M2×5 mm screws (4)
- M3×7 mm screws (6)
- M3×65 mm screws (4)
- M3×10 mm round spacers (16)
- M3 nuts (4)
- M3 square nuts (6)
- M3 washers (8)
- M3 split washers (4)
- Paper towels

16 **Prepare the DMC housing.**

16.1 Make a 2 mm-deep cut along the whole circumference of DMC's plastic housing with a fine plastic saw to cut off the back part of the housing (Fig 5.1). Be careful, deeper cuts may cause damage to the DMC interior. Clean the housing after cutting with a dust blower.

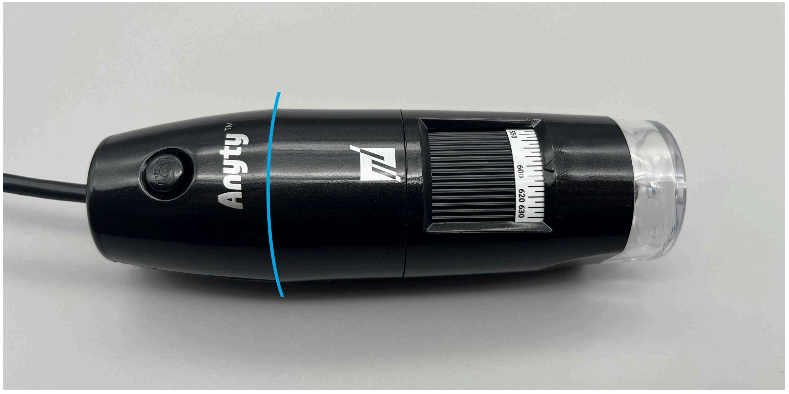

Fig 5.1. Location of the cut.

16.2 Pull the two halves of the housing's back part apart with your thumbs or needle-nose pliers (Fig 5.2). The half with no circuit board attached may be disposed of.

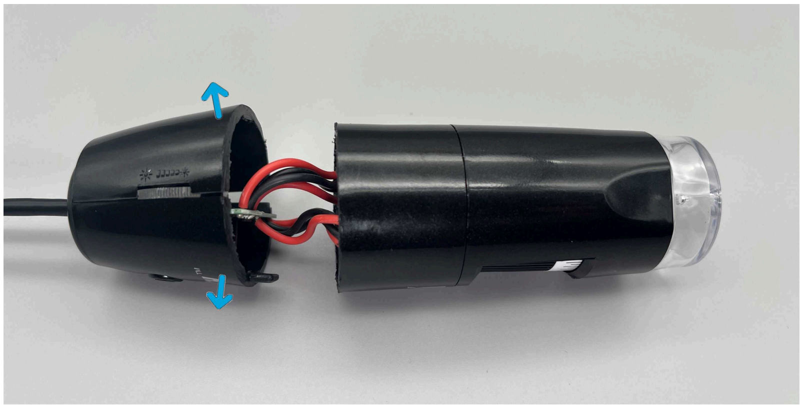

Fig 5.2. Location where the two halves of the housing's back part are pulled apart.

16.3 Unscrew the circuit board with the M2 screwdriver (Fig 5.3). Save the screws for a later step in this section.

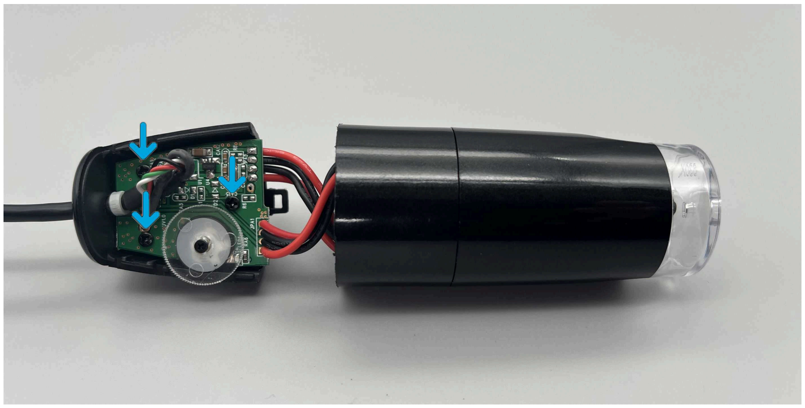

Fig 5.3. Screws to be removed from the circuit board.

16.4 Make two cuts into the back wall of the housing part with the fine plastic saw (Fig 5.4) and remove the housing part from the cable. The separated housing part may be disposed of.

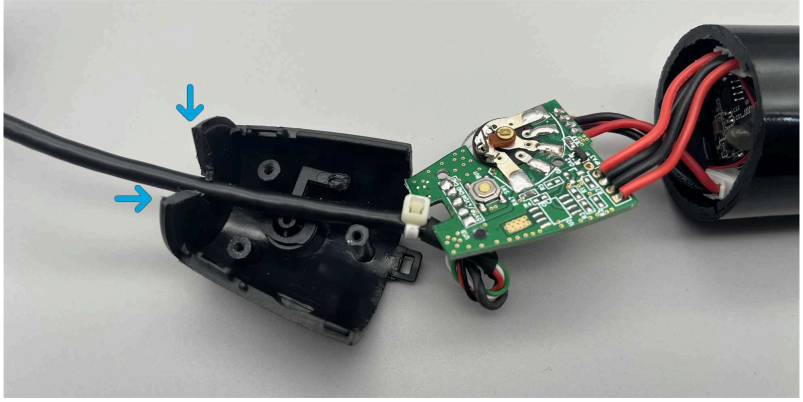

Fig 5.4. Cuts in the back wall of the housing part for separation from the cable

16.5 Remove residual plastic pieces from the back part of the DMC housing by pushing the blade of a box cutter in between the front part of the housing and the pieces from the back part (Figs 5.5, 5.6).

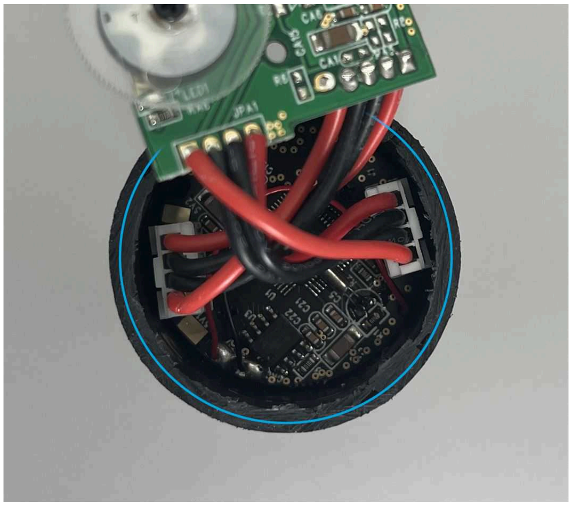

Fig 5.5. Border between the front part of the DMC housing and pieces from the back part.

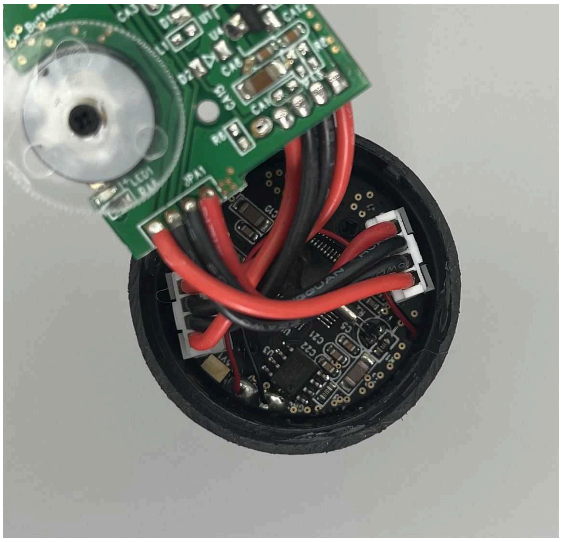

Fig 5.6. Front part of the DMC housing with all pieces from the back part removed.

16.6 Set the magnification wheel of the DMC to 600 and fixate the wheel using a piece of clear tape (Fig 5.7).

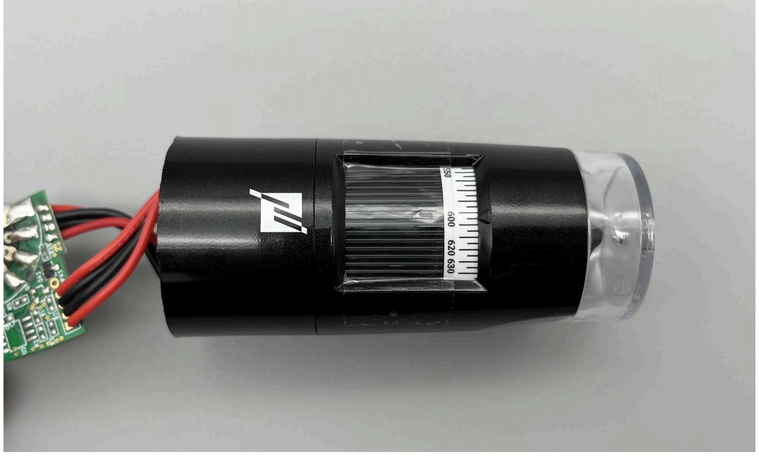

Fig 5.7. The magnification wheel fixated with clear tape.

16.7 *(optional)* For additional fixation, a drop of hot glue may be added to the front of the DMC (Fig 5.8).

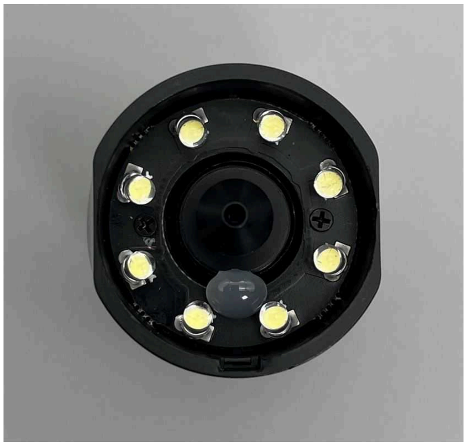

Fig 5.8. DMC lens fixated with hot glue.

17 **Fixate the DMC holder (front) and the F-axis stepper motor to the DMC.**

17.1 Slide the part *dmc-holder-front* onto the front of the DMC. There is some resistance in the beginning. Next, attach the part *dmc-attachment* to the front of the DMC (Fig 5.9).

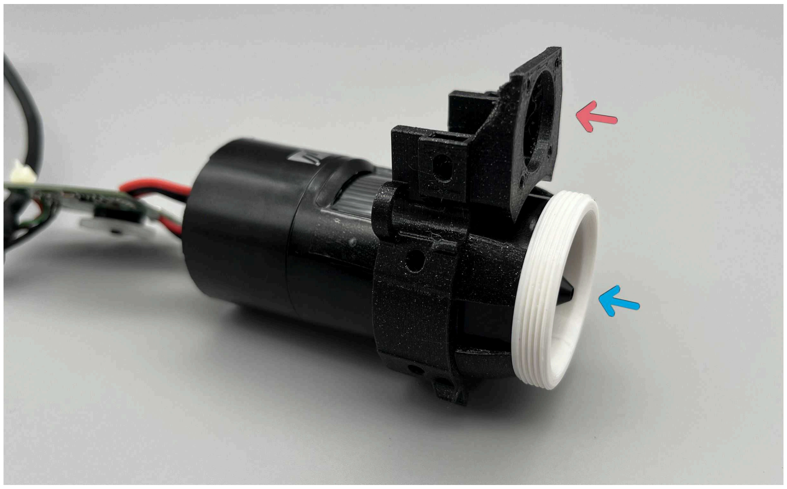

Fig 5.9. DMC with the parts *dmc-holder-front* (blue arrow) and *dmc-attachment* (red arrow) attached.

17.2 Put the DMC on its front. Insert M3 square nuts into the four holes at the back of the part *dmc-holder-front* and push them all the way in with the hex key (Fig 5.10).

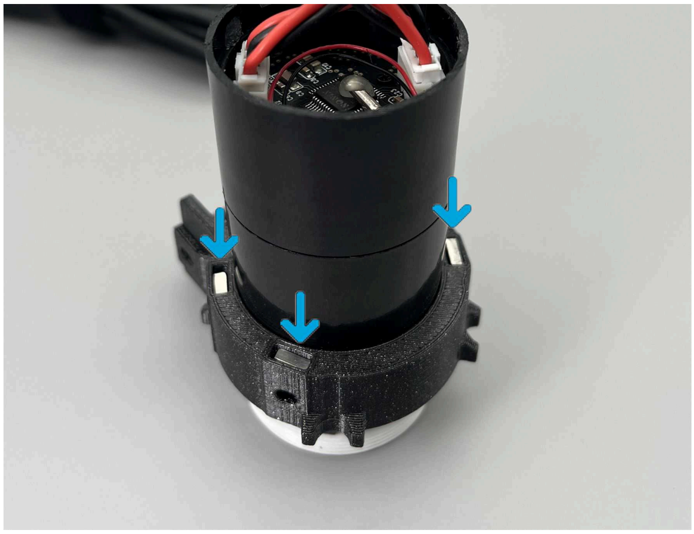

Fig 5.10. Locations for insertion of the M3 square nuts.

17.3 Insert an M3×7 mm screw with an M3 split washer in each of the four holes on the side of the part *dmc-holder-front*. Tighten the screws evenly while keeping the front edges of the part *dmc-holder-front* completely flush with the front edge of the DMC. To achieve this, push both the DMC and the part against the white DMC attachment while tightening (Fig 5.11). The part should be firmly attached to the DMC. The screws may go all the way in.

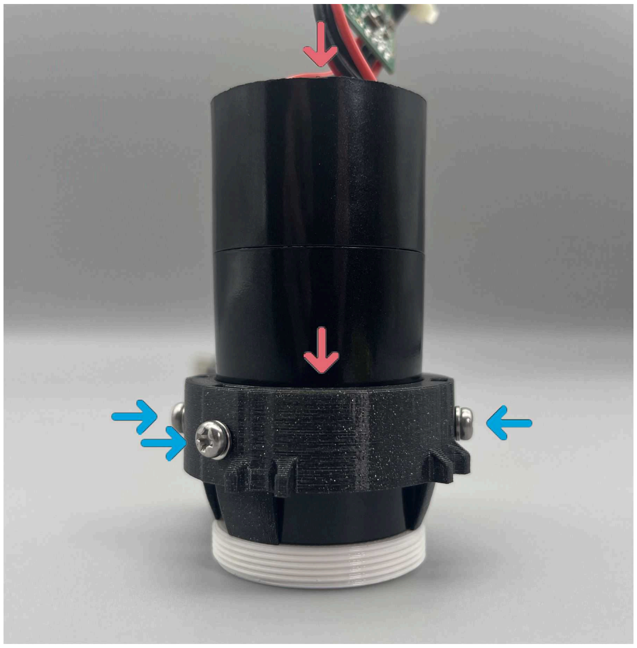

Fig 5.11. Screws fastened to the part *dmc-holder-front* (blue arrows) while applying downward pressure (red arrows).

17.4 Screw the F-axis stepper motor to the top of the part *dmc-holder-front* with four M2×5 mm screws. Then, add the part *f-axis-motor-gear* to the D-shaped shaft of the motor (Fig 5.12).

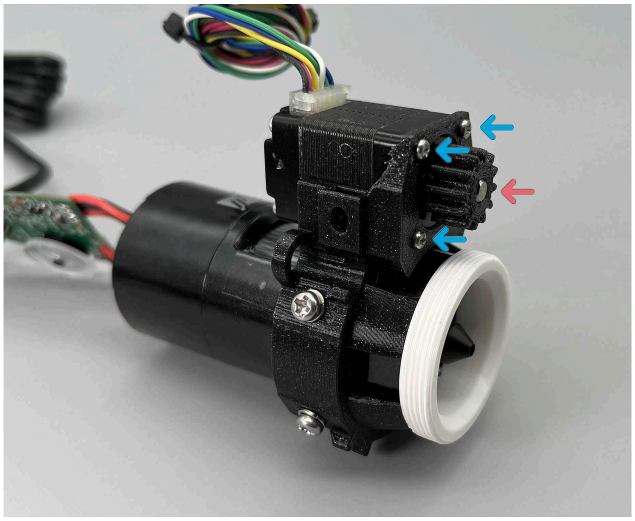

Fig 5.12. DMC with the motor (blue arrows) and the part *f-axis-motor-gear* (red arrow) attached.

18 **Mount the DMC onto the carriage.**

18.1 Cut the compression spring to 20 mm length with a wire flush cutter. There should be eight active coils and one closed end (Fig 5.13).

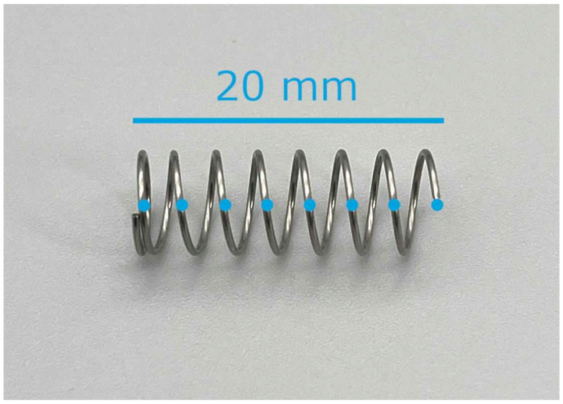

Fig 5.13. Cut compression spring with eight active coils (indicted with dots) and a closed end (on the left).

18.2 Add an M3 washer and three M3×10 mm round spacers to each of the four M3×65 mm screws, to create the four rods that attach to the DMC carriage (Fig 5.14).

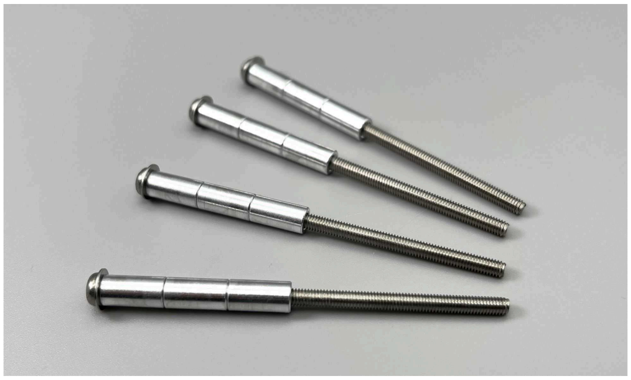

Fig 5.14. M3×65 mm screws with washers and round spacers added.

18.3 Place the DMC into the central opening of the DMC carriage starting with the USB cable (Fig 5.15).

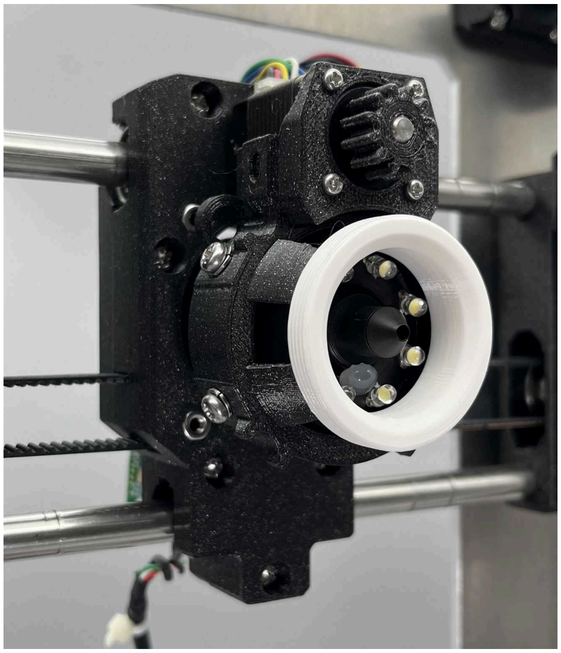

Fig 5.15. DMC inserted into the DMC carriage.

18.4 Insert three of the M3×65 mm screws into the front of the DMC carriage (Fig 5.16). Screw them all the way in, but be careful not to rotate the M3 nuts inside their hexagonal holes.

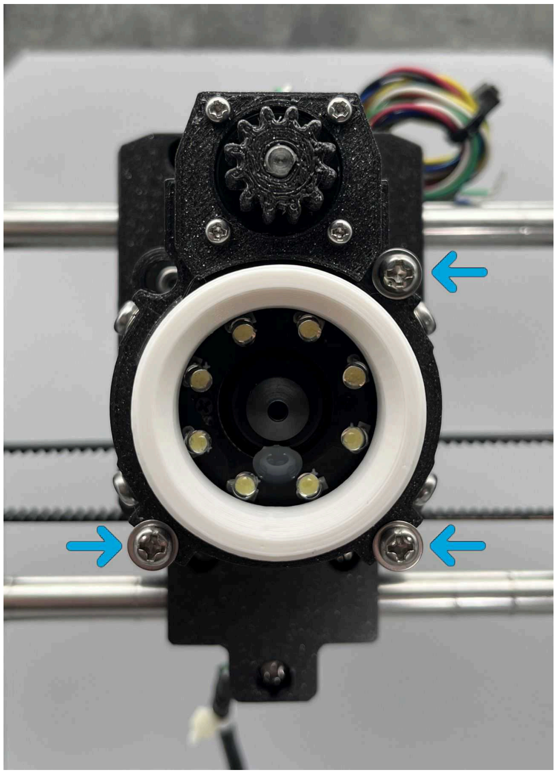

Fig 5.16. DMC carriage with three M3×65 mm screws inserted.

18.5 Insert the fourth M3×65 mm screw into the front of the DMC carriage. Include the compression spring and the part *f-axis-spring-end* between the DMC carriage and the ring of the part *dmc-holder-front* (Fig 5.17). The closed end of the compression spring should face the part *f-axis-spring-end*.

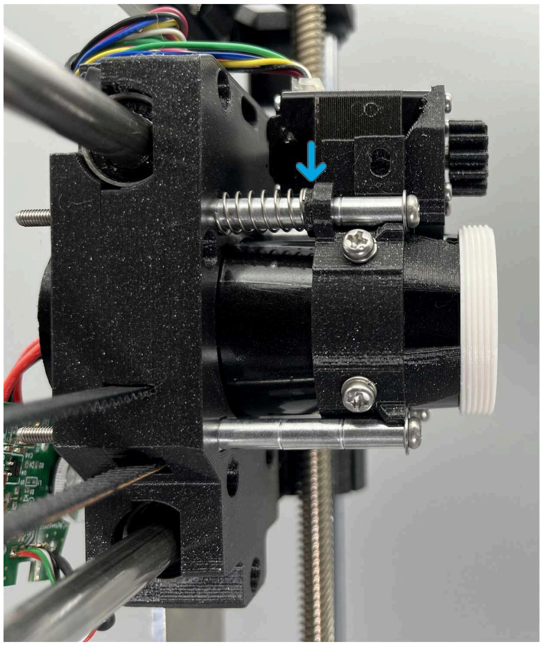

Fig 5.17. The fourth M3×65 mm screw with the compression spring and the part *f-axis-spring-end* (blue arrow)

19 **Attach the DMC holder (back).**

19.1 Add an M3 washer, an M3 nut, and an M3×10 mm round spacer to each of the four M3×65 mm screws in the back of the DMC carriage (Fig 5.18). The M3 nuts are fastened with the wrench. The round spacers are fastened either by hand or using the needle-nose pliers with a paper towel covering their jaws, to not scratch the surfaces of the round spacers.

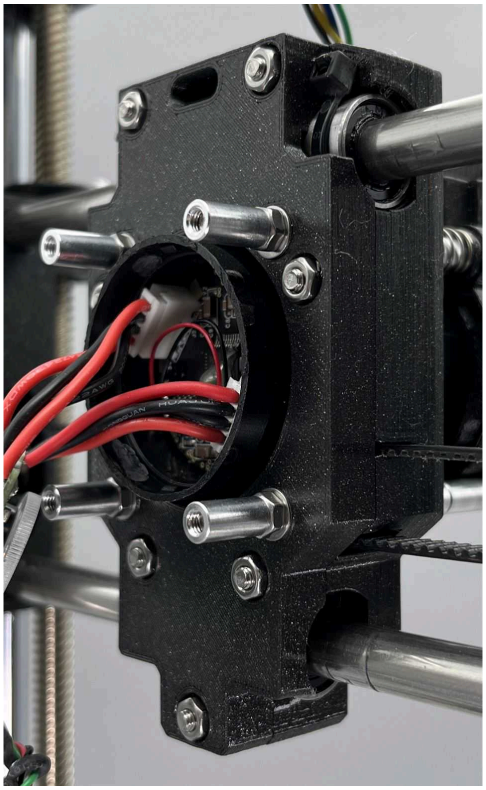

Fig 5.18. M3×65 mm screws with washers, nuts, and round spacers added.

19.2 Guide the USB cable and the circuit board of the DMC through the part *dmc-holder-back*, starting from the side without the three screw holes. The circuit board fits through where the hole diameter is slighter enlarged (Fig 5.19).

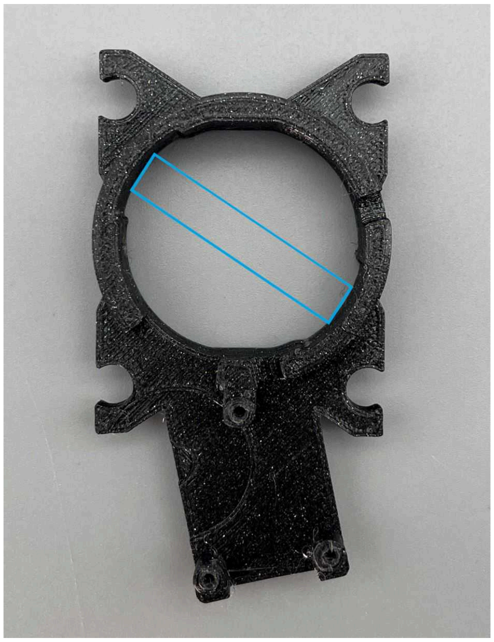

Fig 5.19. Section with wider diameter to fit the DMC's circuit board.

19.3 Attach the part *dmc-holder-back* to the back of the DMC. The DMC housing should thrust into indentations at the top and bottom of the part (Fig 5.20).

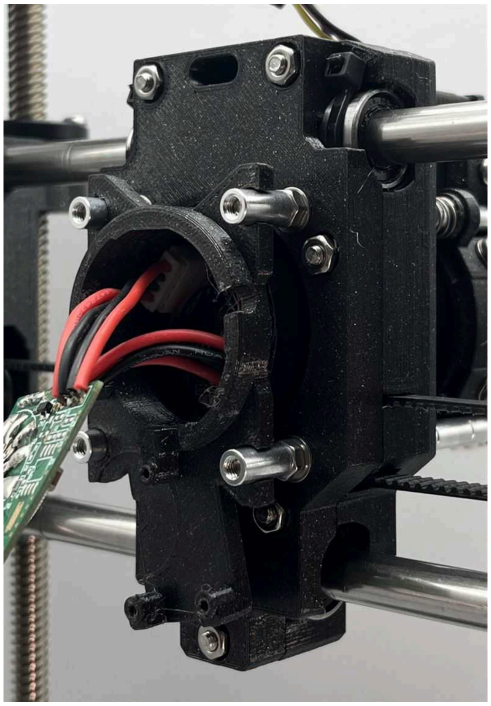

Fig 5.20. DMC with the part *dmc-holder-back* attached.

19.4 Guide the USB cable between the circuit board and the part *dmc-holder-back* and press it into the two available openings (Fig 5.21).

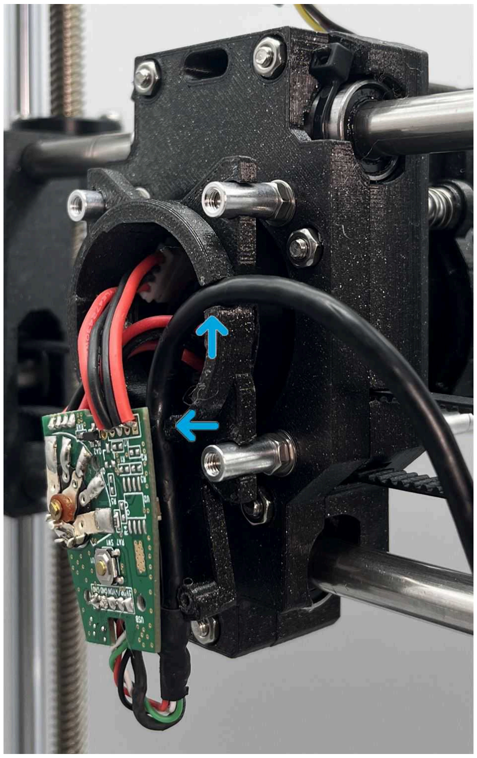

Fig 5.21. Part *dmc-holder-back* with the DMC's USB cable pressed into it.

19.5 Fasten the circuit board to the part *dmc-holder-back* with the three screws originally removed from the circuit board (Fig 5.22).

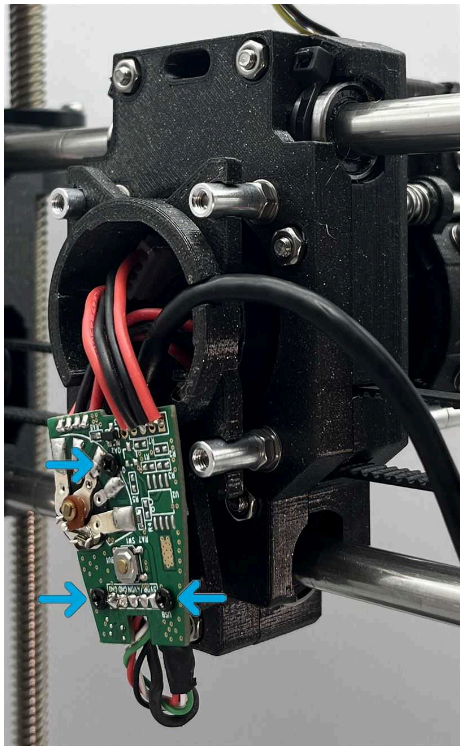

Fig 5.22. Part *dmc-holder-back* with the DMC's circuit board screwed on.

20 **Check, if the DMC slides back and forth easily.**

20.1 Push the DMC backwards. When letting go, the compression spring should push the DMC all the way to the front.

20.2 If the DMC does not slide easily, remove one of the M3×10 mm round spacers in the back of the DMC carriage and try again. The imaging device works perfectly fine with only three round spacers in the back.

20.3 If the DMC still does not slide easily, repeat the assembly of the DMC carriage.

20.4 Adjust how tightly the DMC attaches to the rods of the DMC carriage by using the parts *f-axis-tighteners*. To add the parts, insert an M3 square nut on either side of the F-axis stepper motor (Fig 5.23) and attach the parts with M3×7 mm screws (Fig 5.24). The DMC should barely shift sidewards while still being able to slide back and forth easily.

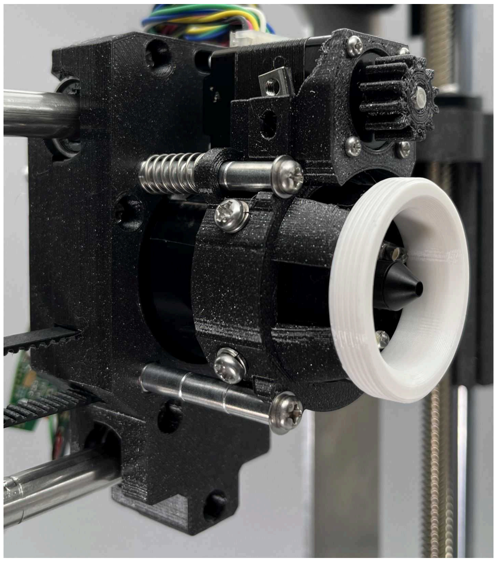

Fig 5.23. Part *dmc-holder-front* with an M3 square nut added.

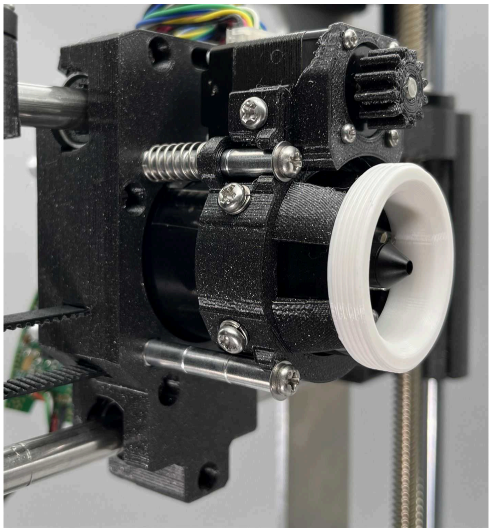

Fig 5.24. Part *dmc-holder-front* with the parts *f-axis-tighteners* attached.

21 **Screw the part *dmc-attachment-gear* onto the front of the DMC attachment (Fig 5.25).**

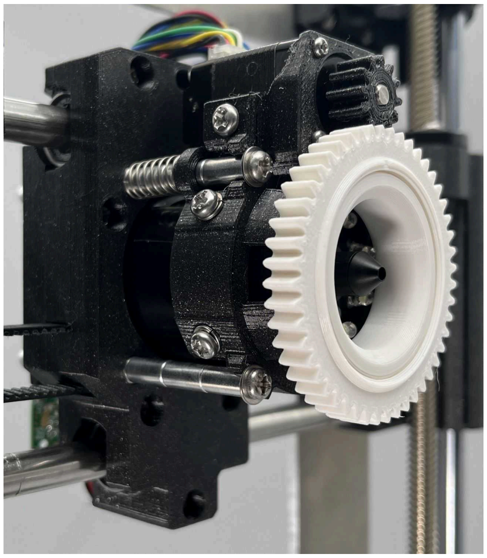

Fig 5.25. Completed DMC carriage with the DMC fully mounted.

Section 6: Assembly and wiring of the control unit

22 **Gather tools and materials.**

Tools:

- Screwdriver (M2)
- Screwdriver (M3)
- Screwdriver (slotted)
- Needle-nose pliers
- Wire flush cutter
- Soldering iron

- Hex key
- Wire stripper
- Clear tape

Materials (quantity):

- Part *frame-hat* (1)
- Part *frame-hat-insert* (1)
- *Raspberry Pi Zero 2 W* (1)
- *DC & Stepper Motor Bonnets* (2)
- Raspberry Pi power supply (1)
- Stepper motor power supply (1)
- USB adapter (1)
- GPIO stacking headers (2)
- Wire AWG22, black and red
- Cable ties (16)
- Solder
- Insulation tape
- M2.5×6 mm screws (4)
- M2.5×12 mm hexagonal spacers (4)
- M2.5×15 mm hexagonal spacers (4)
- M2.5 nuts (4)
- M3×7 mm screw (1)
- M3 square nut (1)
- M3 washers (8)

23 **Attach the part *frame-hat* to the aluminum frame.**

23.1 Add an M3 square nut to the opening in the back of the part *frame-hat* (Fig 6.1). Push it all the way down with the hex key.

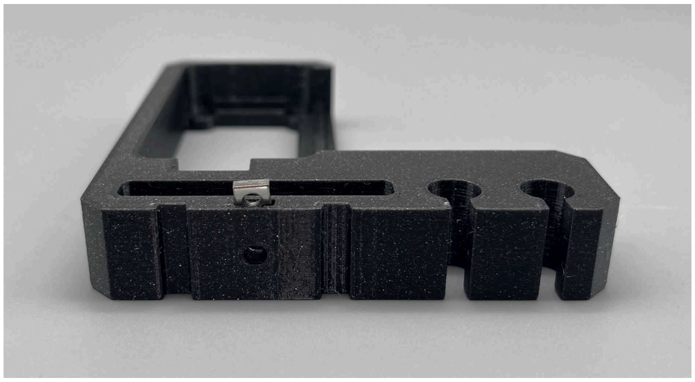

Fig 6.1. Part *frame-hat* with an M3 square nut added.

23.2 Push the part *frame-hat-insert* into the part *frame-hat*, add a M3×7 mm screw, and fasten (Fig 6.2).

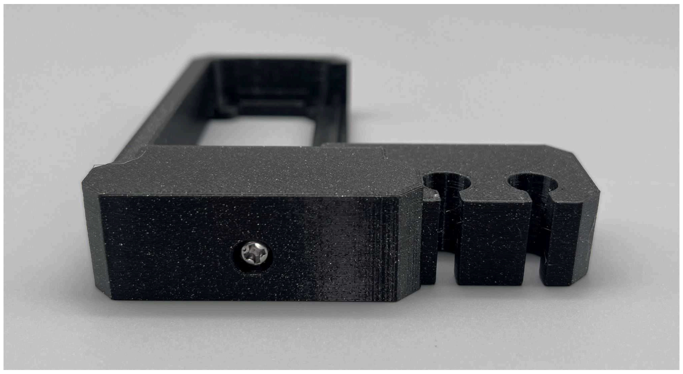

Fig 6.2. Part *frame-hat* with the part *frame-hat-insert* added.

23.3 Push the upper right corner of the aluminum frame into the opening on the bottom of the part *frame-hat* (Fig 6.3).

Note

If the sheet to produce the aluminum frame was 3 mm (rather than 0.125 inch) thick, use 2 - 3 layers of clear tape to thicken the upper right corner of the aluminum frame, so that it fits tightly into the slot of the part *frame-hat*.

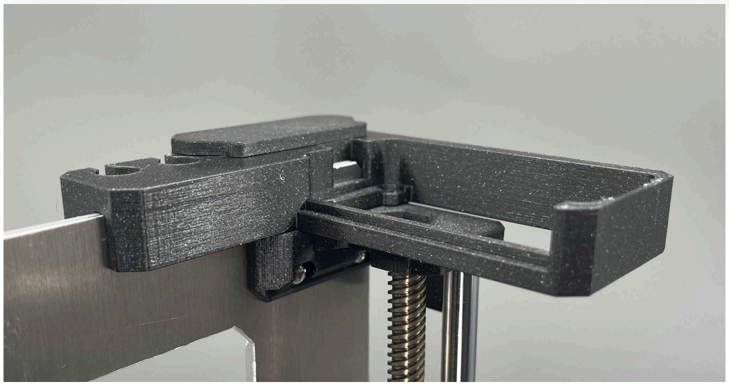

Fig 6.3. Aluminum frame with the part *frame-hat* added.

24 **Prepare and add the control unit.**

24.1 Since two *DC & Stepper Motor Bonnets* are used at the same time, the Inter-Integrated Circuit (I2C) address of one of them has to be changed. For that, connect the two A0 patches on the bottom of one bonnet with solder (Fig 6.4). For details, see the section ***Stacking hats*** of the tutorial *Adafruit DC and Stepper Motor HAT for Raspberry Pi*.

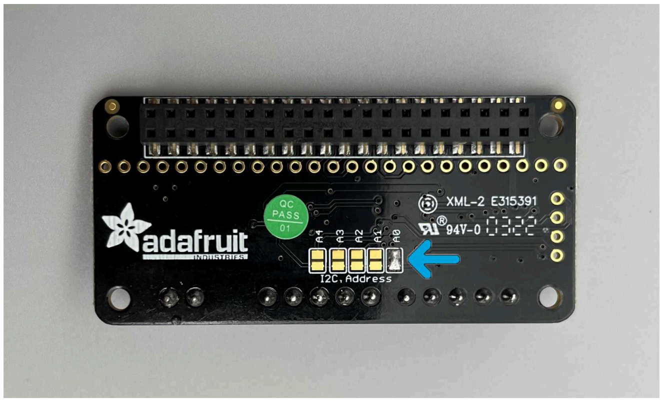

Fig 6.4. One of the *DC & Stepper Motor Bonnets* with its I2C address changed.

24.2 Stack the two *DC & Stepper Motor Bonnets* onto the *Raspberry Pi Zero 2 W* using the GPIO stacking headers, the hexagonal spacers, the M2.5×6 mm screws, and the M2.5 nuts (Fig 6.5). Fasten with the M3 screwdriver and the needle-nose pliers. The bonnet with the modified I2C address is put on top. The boards of the *Raspberry Pi Zero 2 W* and the first bonnet are about 15 mm apart, the boards of the first and second bonnet about 12 mm. Add M3 (or M2.5) washers to fill any height gaps.

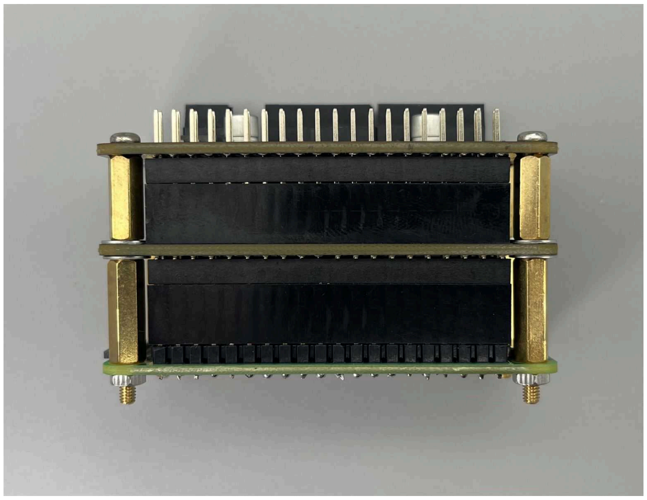

Fig 6.5. Back view of the stacked control unit.

24.3 Insert the control unit into the part *frame-hat* at the top of the aluminum frame (Fig 6.6).

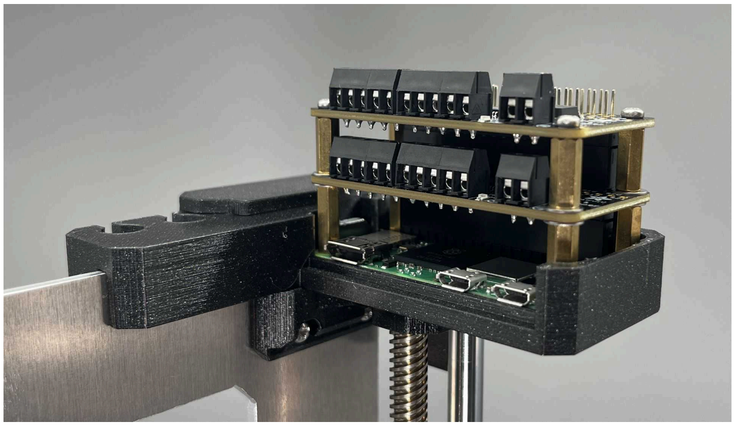

Fig 6.6. Control unit inserted in the part *frame-hat*.

25 **Guide the Z-axis and X-axis motor wires to the control unit.**

Note

This step requires wire lengths of over 55 cm; extend the wires, if necessary.

25.1 Cut off the plugs at the end of the Z-axis motor wires (Fig 6.7).

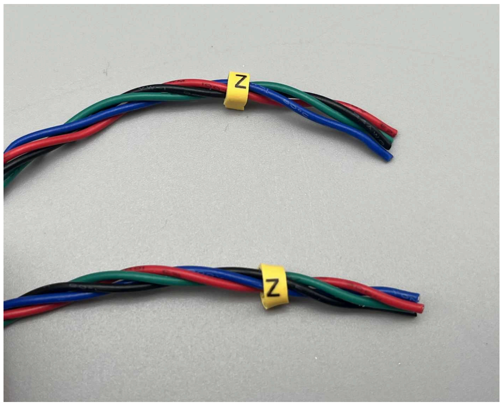

Fig 6.7. Z-axis motor wires with their plugs cut off.

25.2 Guide the Z-axis motor wires through the holes in the bottom of the aluminum frame (Fig 6.8). For this, the yellow label rings need to be removed temporarily. Be careful not to scratch the wire insulation at the edges of the holes. You may need to smooth the edges, if they are sharp or rough.

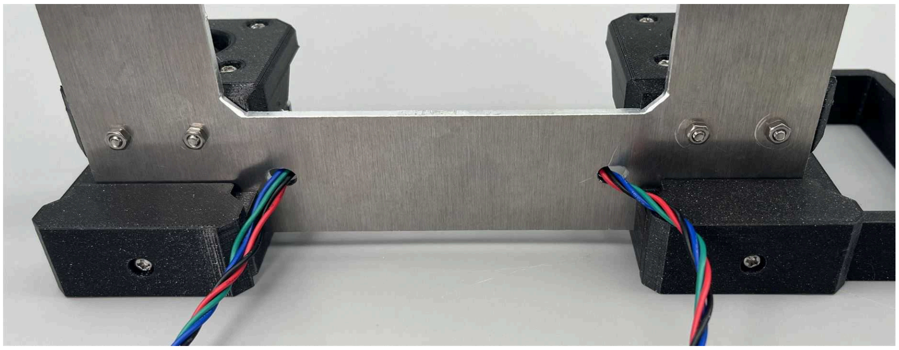

Fig 6.8. Z-axis motor wires guided through the two holes at the bottom of the aluminum frame.

25.3 To prevent any loss of wire insulation during operation of the device, add a piece of insulation tape to each wire bundle where they go through the holes of the aluminum frame (Fig 6.9).

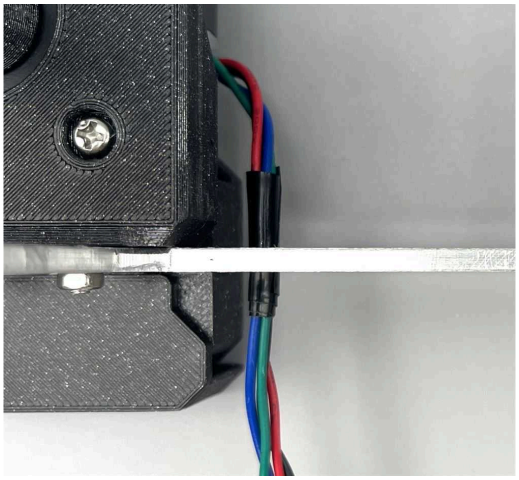

Fig 6.9. Z-axis motor wires with insulation tape added.

25.4 Tie the two bundles of Z-axis motor wires together at the bottom of the frame (Fig 6.10).

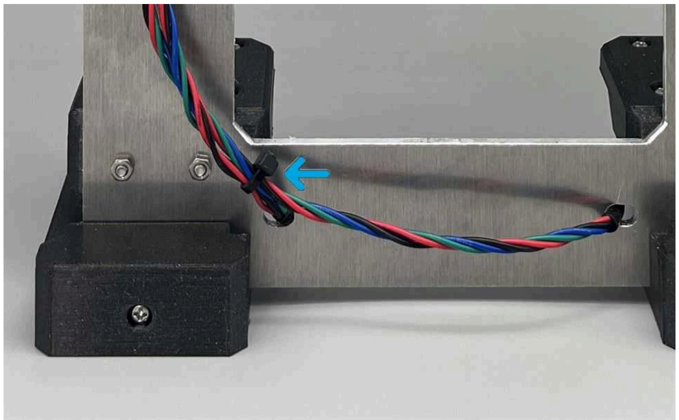

Fig 6.10. Z-axis motor wires tied together at the bottom of the frame.

25.5 Twist the two bundles of Z-axis motor wires together (lightly), guide them through the left hole in the *frame-hat*, and add two more cable ties (Fig 6.11). You may also fixate the wires to the back of the aluminum frame with a piece of clear tape.

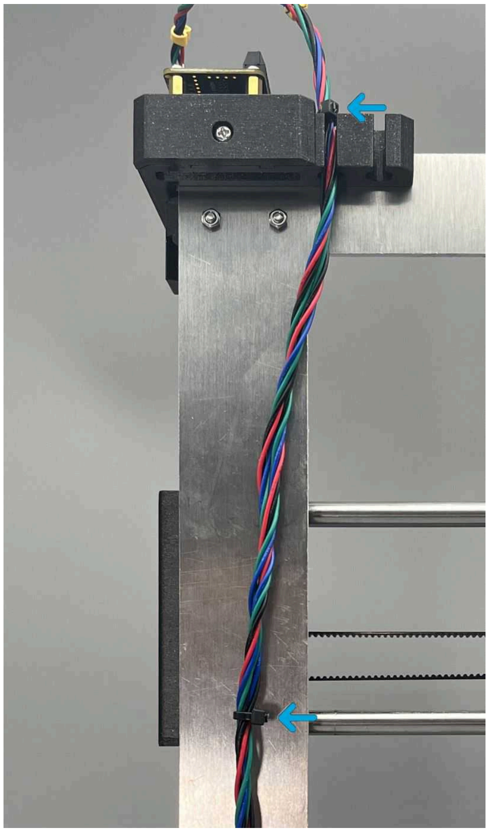

Fig 6.11. Z-axis motor wires tied together at the center and top of the frame.

25.6 Attach the X-axis motor wires to the part *z-axis-top-mod* above the motor with a cable tie (Fig 6.12). Leave about 28 cm of wire between the cable tie and the X-axis motor. Protect the cable with insulation tape at the edge of the frame.

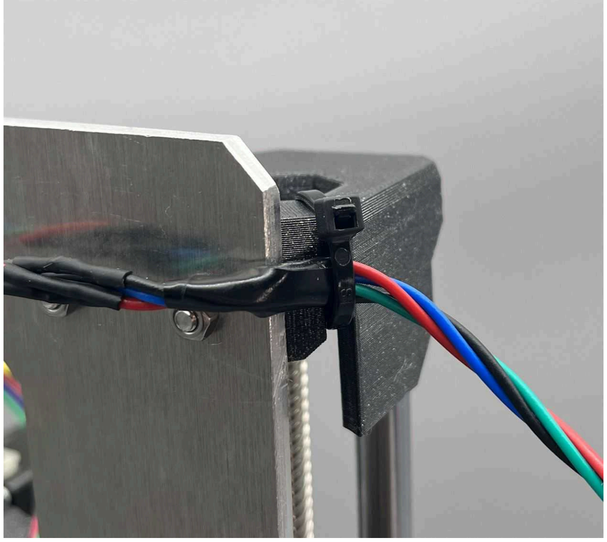

Fig 6.12. X-axis motor wires attached to the part *z-axis-top-mod*.

25.7 Guide the X-axis motor wires through the same hole in part *frame-hat* as the Z-axis motor wires and tie them together just below the part *frame-hat* (Fig 6.13).

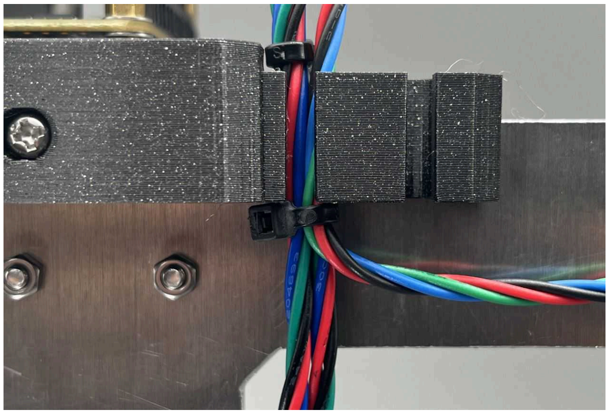

Fig 6.13. X-axis motor wires tied to the Z-axis motor wires.

26 **Guide the F-axis motor wires to the control unit.**

Note

This step requires wire lengths of over 60 cm; extend the wires, if necessary.

26.1 Wind the F-axis motor wires twice, secure them with two cable ties (Fig 6.14).

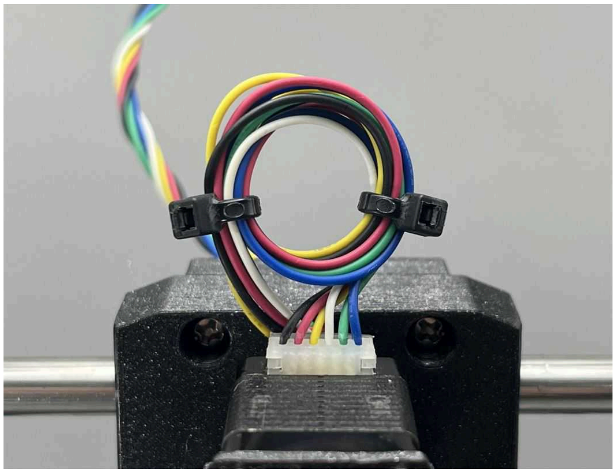

Fig 6.14. Winded F-axis motor wires.

26.2 Guide the F-axis motor wires through the hole at the top of the DMC carriage and secure them in place with a cable tie at the back (Fig 6.15). The wire bundle should be flat when entering the hole. You may use a piece of clear tape to keep it flat.

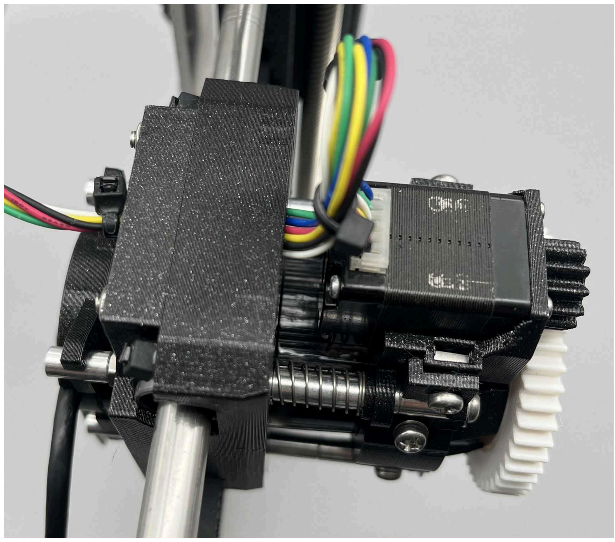

Fig 6.15. F-axis motor wires going through the DMC carriage.

26.3 Tie the F-axis motor wires and the USB cable of the DMC together near the DMC carriage with a cable tie (Fig 6.16). There should be about 4 cm of USB cable and 5 cm of F-axis motor wire between the DMC carriage and the cable tie. (Lightly) twist the wires and the cable together, and guide them towards the part *frame-hat*.

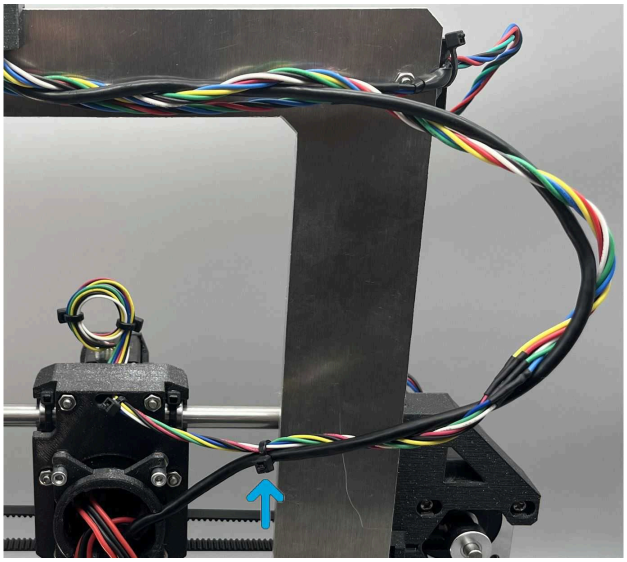

Fig 6.16. Twisted F-axis motor wires and USB cable with the location for the cable tie marked.

26.4 Add the twisted wires and cable to the empty hole in the back of the part *frame-hat* and add cable ties below and above (Fig 6.17). There should be about 30 cm of twisted wires/cable between the cable tie near the DMC carriage and the part *frame-hat*. The twisted wires and cable should be bent as in Fig 6.16 during operation of the imaging device.

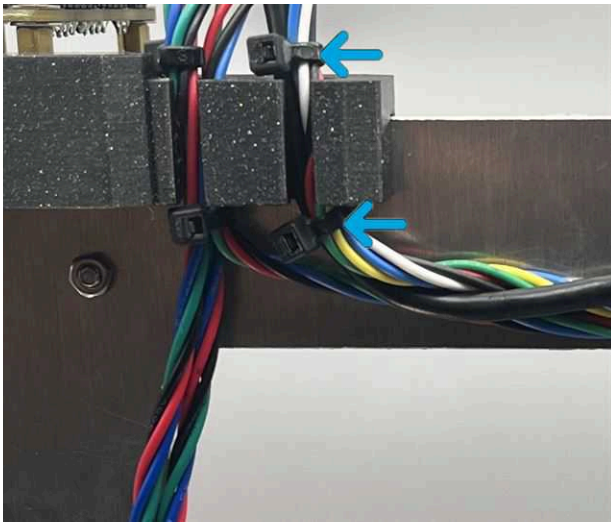

Fig 6.17. F-axis motor wires and USB cable with the locations for the cable ties marked.

27 **Connect the motor wires to the control unit.**

27.1 Shorten the motor wire bundles to about 10 cm above the part *frame-hat*. Then, strip about 5 mm of insulation from the end of all motor wires (Fig 6.18).

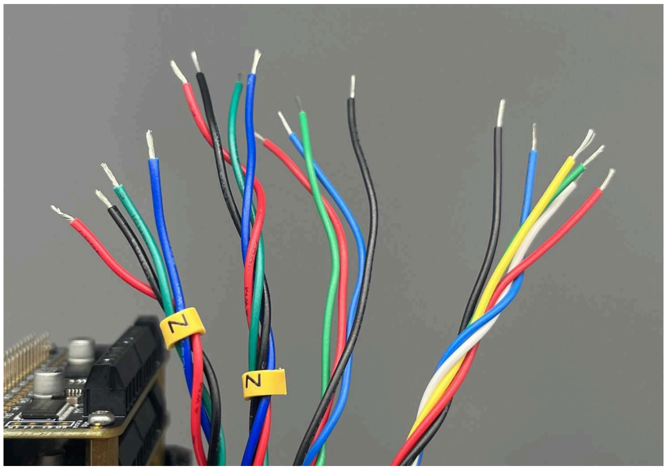

Fig 6.18. Motor wires stripped.

27.2 Additionally, cut two 5 cm-long pieces of each black and red AWG22 wire and strip 5 mm of insulation from all wire ends (Fig 6.19). The wire pieces are needed to connect the stepper motor power supply to the control unit.

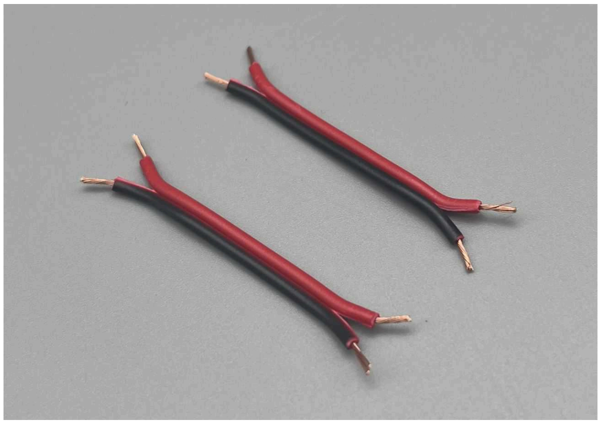

Fig 6.19. Wires for the stepper motor power supply cut and stripped.

27.3 Remove the top *DC & Stepper Motor Bonnet* from the control unit (Fig 6.20).

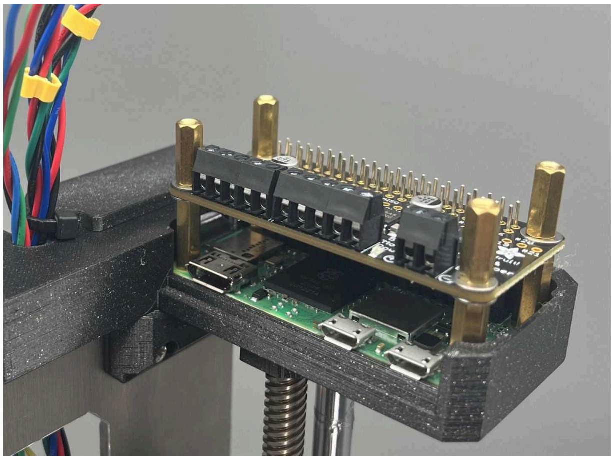

Fig 6.20. Control unit with the top *DC & stepper Motor Bonnet* removed.

27.4 Connect the Z-axis motor wires to the bottom *DC & Stepper Motor Bonnet* with the M2 screwdriver as in Fig 6.21. One motor attaches to the terminals M1 and M2, the other one to M3 and M4. The order does not matter, as both Z-axis motors are always moved simultaneously.

Note

If stepper motors other than the Z-axis motors sold by Prusa Research are used, wires may connect differently. For further details, consult the section ***Using Stepper Motors*** of the tutorial *Adafruit DC and Stepper Motor HAT for Raspberry Pi*.

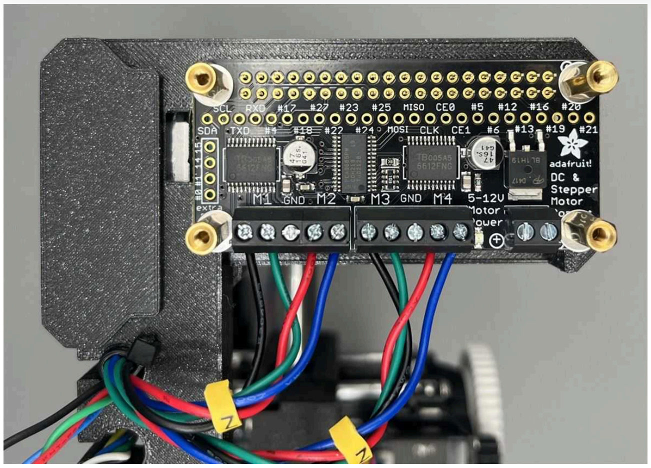

Fig 6.21. Z-axis motor wires connected to the bottom *DC & Stepper Motor Bonnet*

27.5 Connect a pair of black and red AWG22 wire to the power terminal with the slotted screwdriver (Fig 6.22). The red wire is attached to the plus terminal, the black one to the minus terminal.

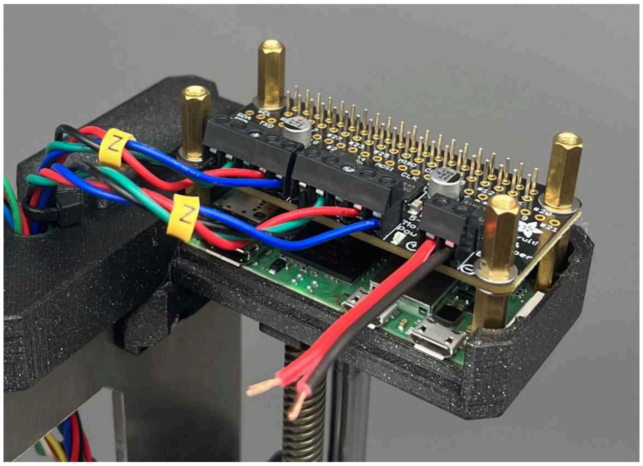

Fig 6.22. AWG22 wire connected to the bottom *DC & Stepper Motor Bonnet.*

27.6 Reattach the top *DC & Stepper Motor Bonnet* to the control unit and connect the X-axis and F-axis motor wires as well as the second pair of black and red AWG22 wire (Fig 6.23). The X-axis motor wires attach to the terminals M1 and M2, the F-axis motor wires to M3 and M4. If unipolar motors are used, they also attach to the GND terminal (see the F-axis motor wires in Fig 6.23).
For further details, consult the section *Using* ***Stepper Motors*** of the tutorial *Adafruit DC and Stepper Motor HAT for Raspberry Pi.*

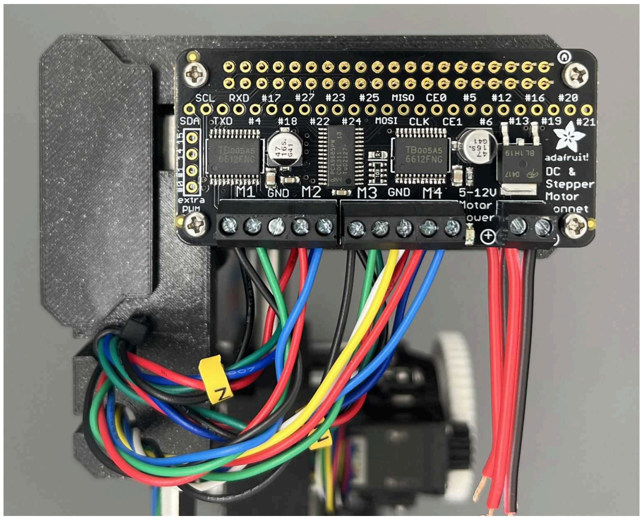

Fig 6.23. X-axis and F-axis motor wires connected to the top *DC & Stepper Motor Bonnet.*

28 **Connect the DMC to the control unit.**

28.1 Connect the USB cable of the DMC to the control unit using the USB-A to micro-USB adapter (Fig 6.24). In Fig 6.24, a barely visible adapter without a plastic housing was used.

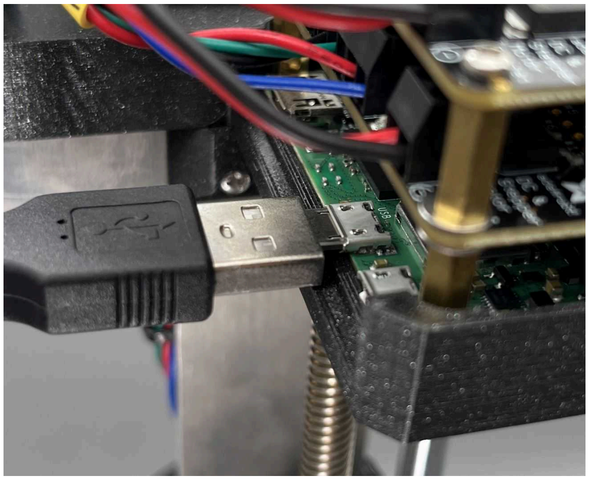

Fig 6.24. USB cable attached to the control unit with an USB-A to micro-USB adapter.

28.2 Secure the USB cable of the DMC to the part *z-axis-top-mod* above the X-axis motor with a cable tie. Also tie the USB cable to itself just behind the USB plug (Fig 6.25).

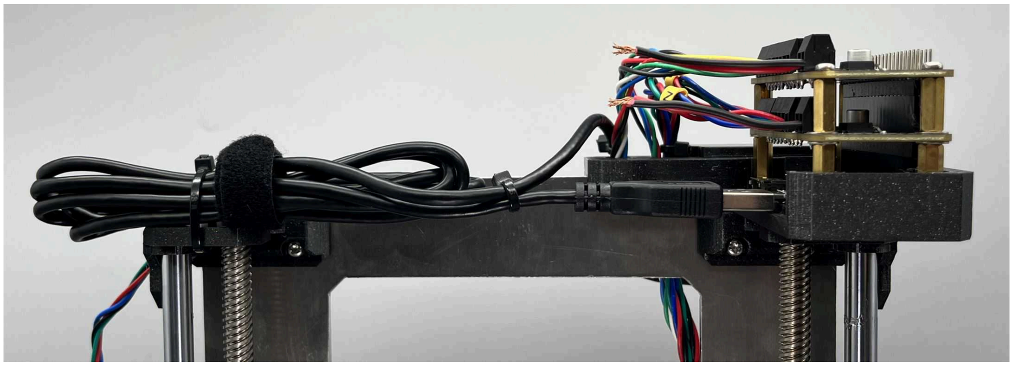

Fig 6.25. USB cable secured to the top of the device.

29 **Connect the power supplies to the control unit.**

29.1 Connect the two pieces of black and red AWG22 wire to the terminal connector of the stepper motor power supply (Fig 6.26). Red wire ends are attached to the plus terminal, black wire ends to the minus terminal. Tie the bottom and top wires together.

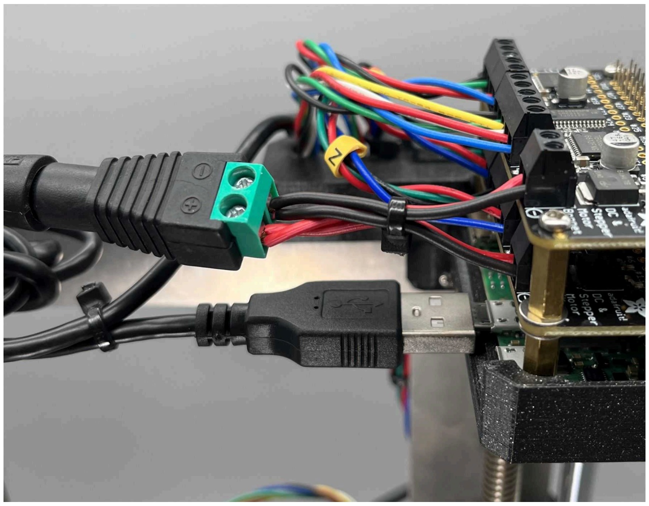

Fig 6.26. AWG22 wire connected to the terminal plug of the power adapter for the motors.

29.2 Connect the micro-USB plug of the Raspberry Pi power supply to the control unit (Fig 6.27).

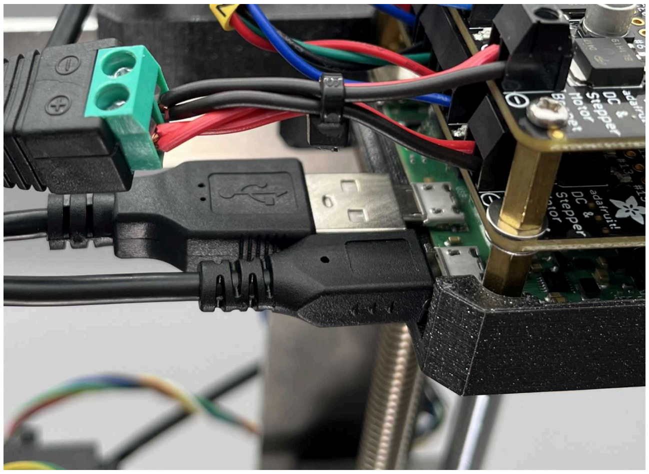

Fig 6.27. Micro-USB plug of the Raspberry Pi power supply connected to the control unit.

29.3 Secure the cables of both power supplies to the USB cable of the DMC and the part *z-axis-top-mod* above the X-axis motor (Fig 6.28).

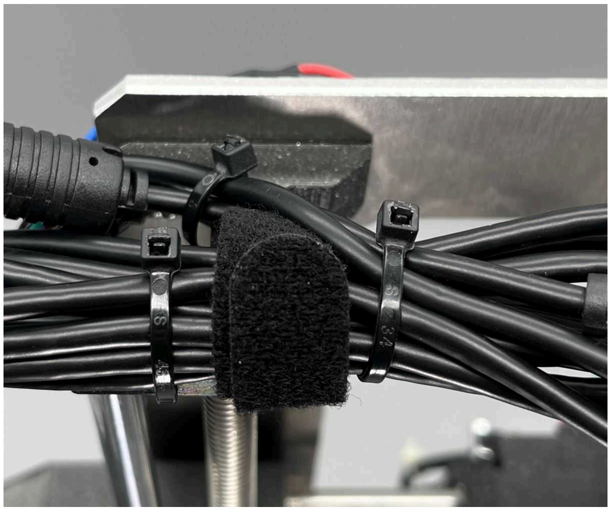

Fig 6.28. Cables of both power supplies secured with two cable ties.

30 **The completed imaging device should resemble the one in Fig 6.29.**

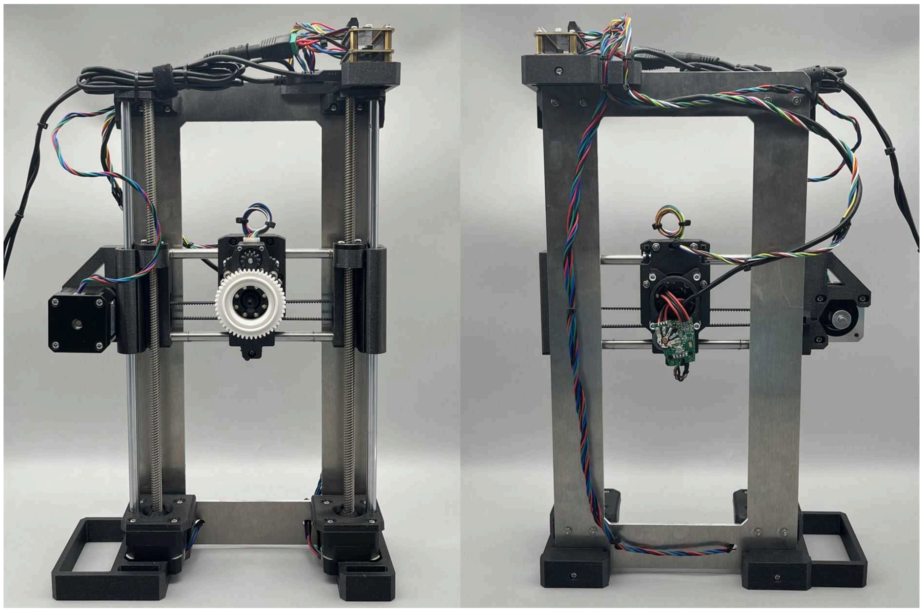

Fig 6.29. Front and back view of the completed imaging device

Section 7: Assembly of the observation box

31 **Gather tools and materials.**

Tools:

- Silicone sealant extruder
- Silicone sealant smoothing tool
- Measure
- Permanent marker
- Needle-nose pliers

- Application device for solvent-based adhesive

Materials (quantity):

- Part *box-wall* (1)
- Part *box-wall-cable* (1)
- Part *box-bottom* (1)
- Part *box-lid* (1)
- Part *box-lid-frame* (1)
- Parts *box-hook* (4)
- Part *box-lid-valve-base* (1)
- Glass sheets (2)
- Protective vent (1)
- Silicone sealant
- Solvent-based adhesive for acrylic
- Masking tape
- String
- Opaque tape or foil

Note

This section does not go into detail on how to work with silicone sealant and solvent-based adhesives for acrylic. For more information, please, refer to the numerous tutorials published online.

32 **Join the box walls with the box bottom.**

32.1 Put the parts *box-bottom, box-wall, and box-wall-cable* on their sides and connect their short edges with masking tape. The short edges of the parts *box-wall and box-wall-cable* should sit on the top flat side of the part *box-bottom* as in Fig 7.1. The slots cut in each of the parts should point towards each other.

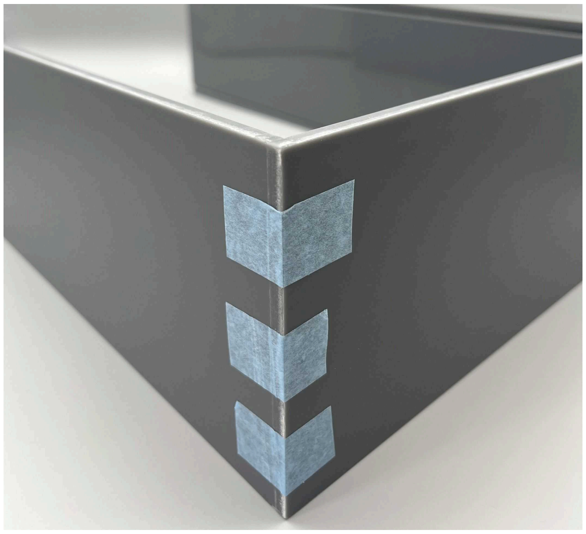

Fig 7.1. Parts *box-wall-cable* (front left) and *box-bottom* (front right) joined with masking tape.

32.2 Make sure that the two cable holes of the part *box-wall-cable* point away from the part *box-bottom* (Fig 7.2).

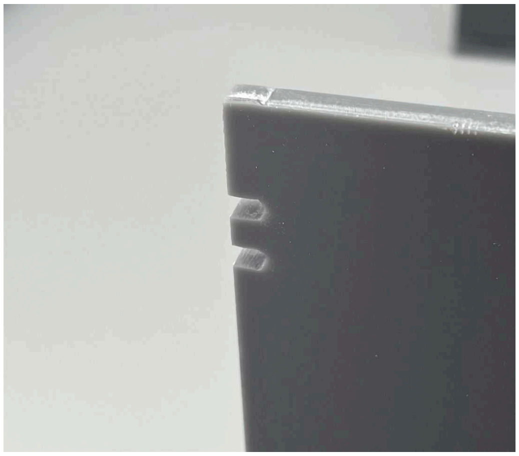

Fig 7.2. Cable holes of the part *box-wall-cable*.

32.3 Make sure that the slots of all the parts are aligned (Fig 7.3).

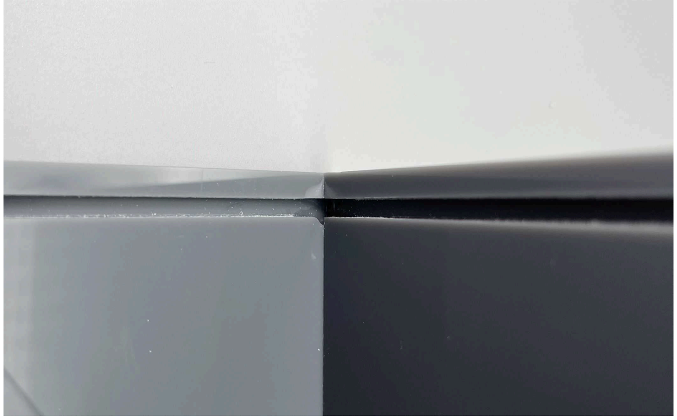

Fig 7.3. Aligned slots of the parts *box-wall-cable* and *box-bottom*.

32.4 To fix alignment, raise parts relative to each other by adding layers of masking tape to their long edges (Fig 7.4). The masking tape holding the parts together will have to be temporarily removed.

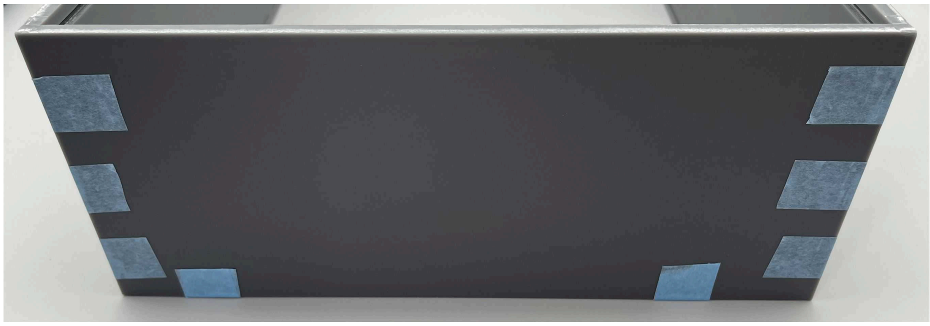

Fig 7.4. Part *box-bottom* raised to align the slots of all three parts.

32.5 Make sure that the parts are in a 90°-angle towards each other.

32.6 Apply the solvent-based adhesive along the joints between the three acrylic parts (Fig 7.5).

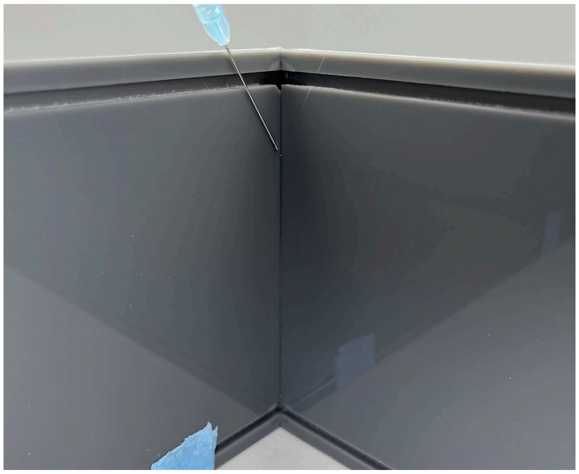

Fig 7.5. Application of the solvent-based adhesive.

32.7 Wait for 20 minutes to let the parts bond. Then, remove all masking tape.

33 **Add the glass sheets.**

33.1 Put the joined acrylic parts upright and slide the glass sheets into their slots (Fig 7.6). The glass sheets should go all the way into the slots of the part *box-bottom* and be flush with the top edges of both box walls.

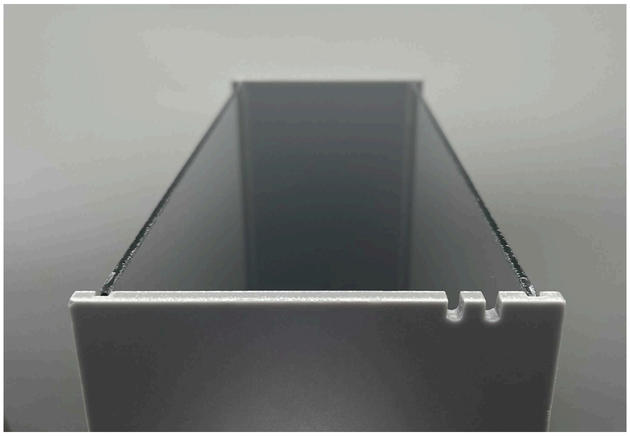

Fig 7.6. Top of observation box with glass sheets inserted.

33.2 Secure the glass sheets to the box walls with two pieces of masking tape (Fig 7.7)

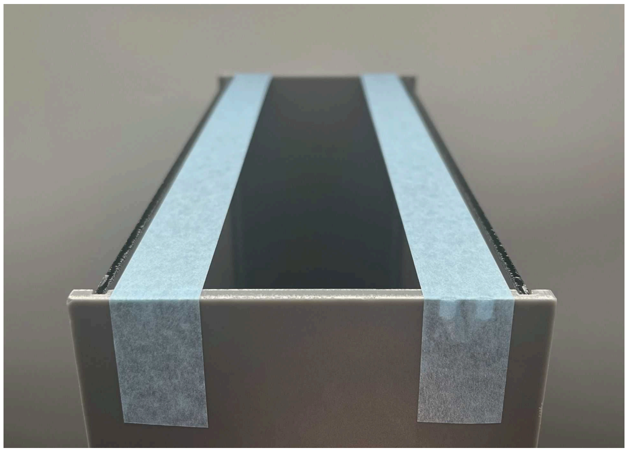

Fig 7.7. Top of observation box with glass sheets secured.

34 **Apply the sealant.**

34.1 Put the acrylic box back on its side and add masking tape to the upward-facing glass sheet in a distance of 7 mm from the acrylic parts (Fig 7.8).

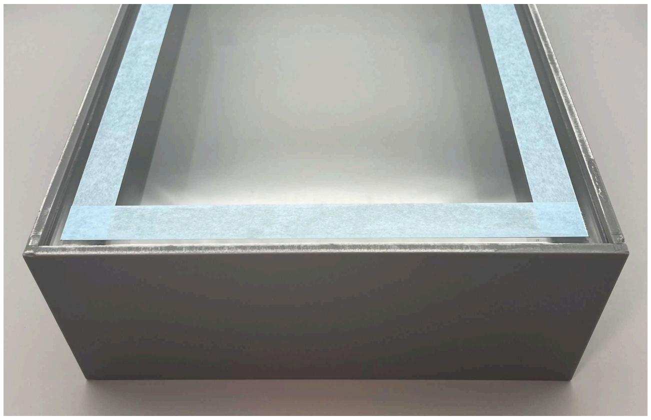

Fig 7.8. Upward-facing glass sheet with masking tape added.

34.2 With the silicone sealant extruder, apply sealant to the space between the masking tape and the acrylic parts (Fig 7.9). During the sealant application, point the extruder towards the slots to fill all gaps between the glass sheet and the surrounding acrylic parts.

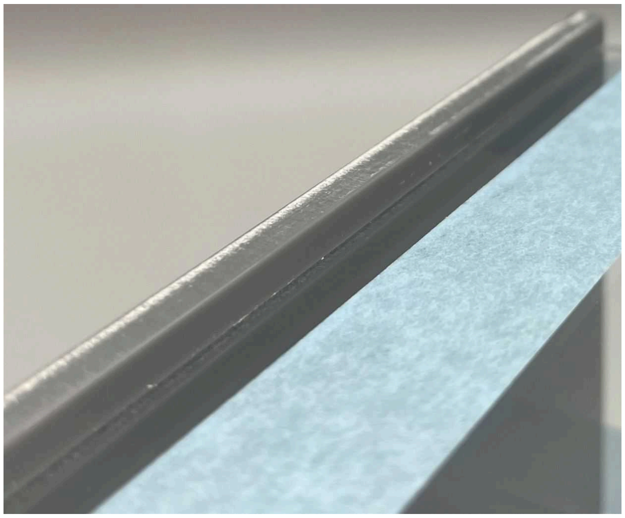

Fig 7.9. Space between the masking tape and the acrylic parts.

34.3 After the sealant application, use the smoothing tool to remove excess sealant (Fig 7.10).

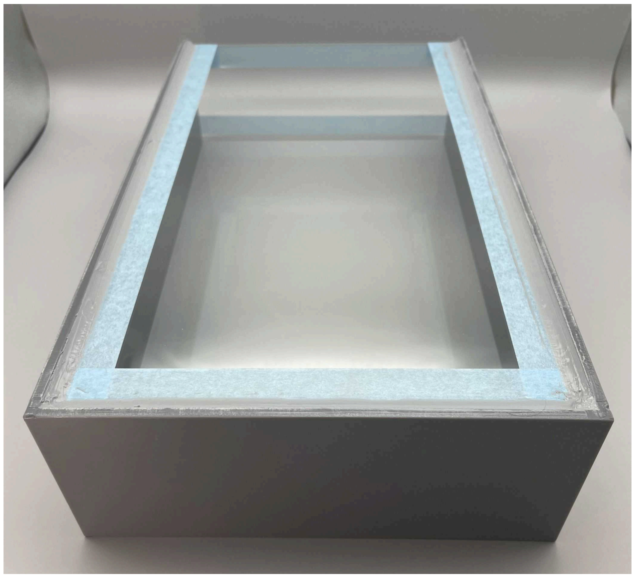

Fig 7.10. Observation box with sealant applied.

34.4 Remove the masking tape after about 10 minutes (Fig 7.11).

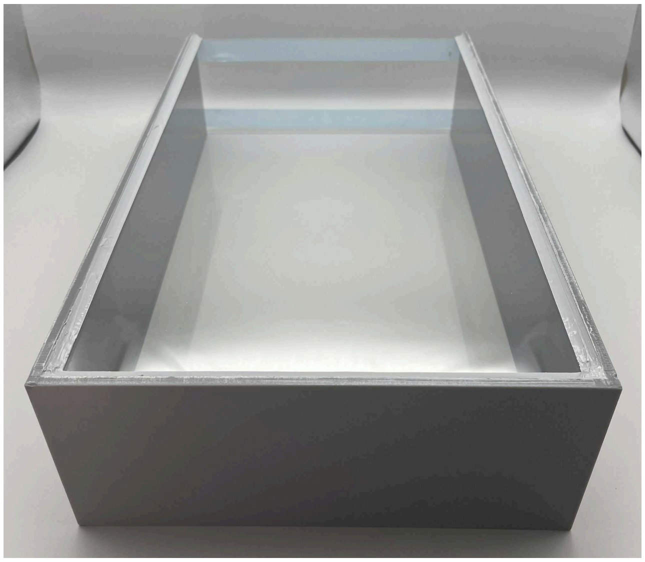

Fig 7.11. Observation box with the masking tape removed.

34.5 Let the silicone sealant dry for at least three hours before turning the box. After that, repeat this step for the opposite side of the observation box.

35 **Add the hooks.**

35.1 Mark the locations for the attachment of the parts *box-hook*, 4 cm away from the top and 1 cm away from each side of each box wall (Fig 7.12).

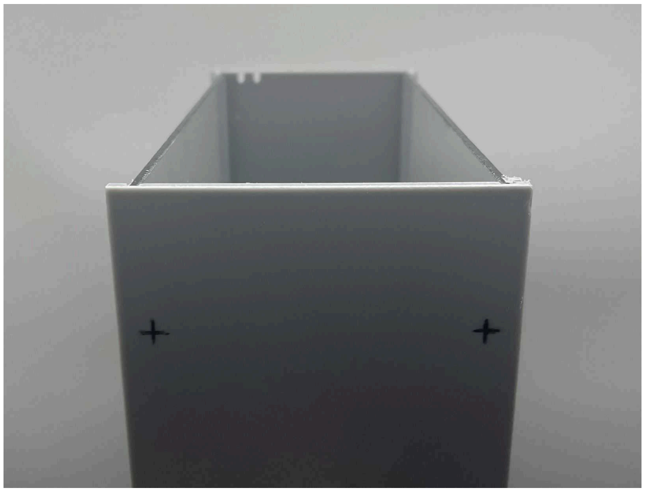

Fig 7.12. Marked locations for the hook attachment.

35.2 Hold the parts *box-hook* to the marked locations and apply solvent-based adhesive (Fig 7.13).

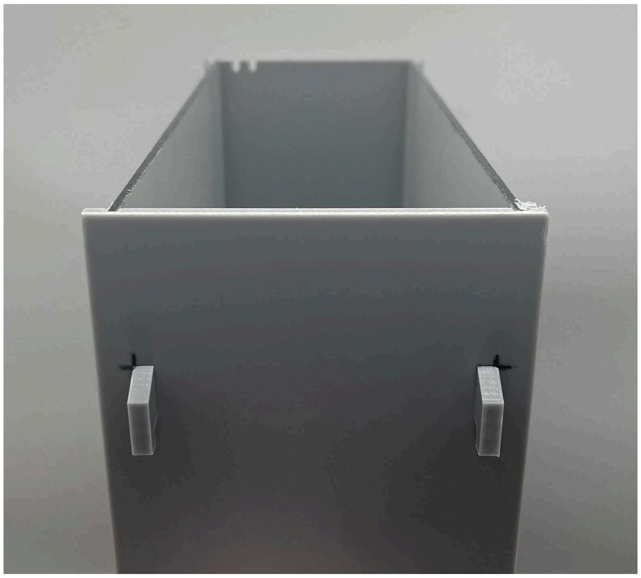

Fig 7.13. Hooks attached to the observation box.

36 **Assemble the lid of the observation box.**

36.1 Put the part *box-lid* on its flat side, so that its pockets face upwards. Fixate the part *box-lid-frame* on top of it with masking tape, so that the two indentations point upwards (Fig 7.14).

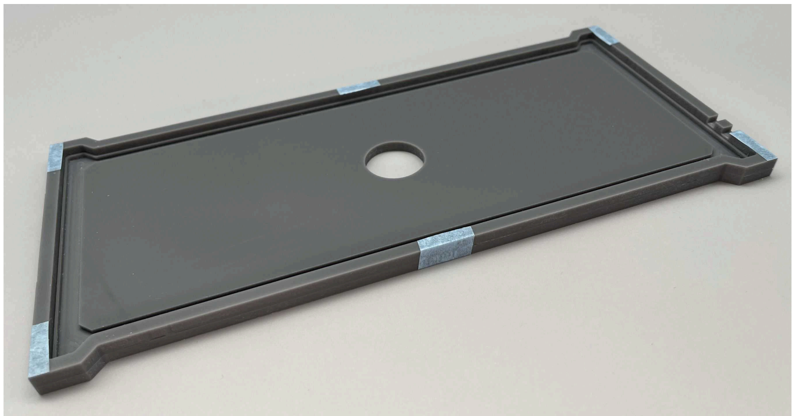

Fig 7.14. The part *box-lid-frame* fixated on top of the part *box-lid*.

36.2 Apply solvent-based adhesive to combine the two parts, wait for 10 min, and remove the masking tape.

36.3 Turn the combined parts upside down, put the part *box-lid-valve-base* right above the central hole, and fixate the part with masking tape (Fig 7.15).

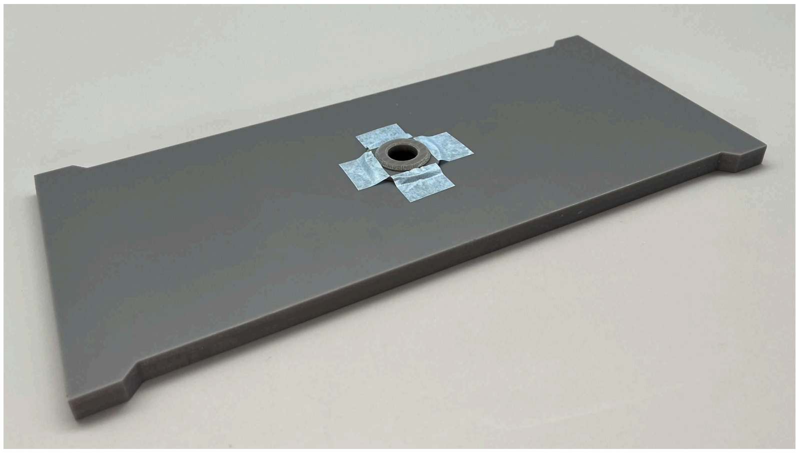

Fig 7.15. Lid with the part *box-valve-base* fixated.

36.4 Turn the combined parts back to their position in Fig 7.14 and fixate the part *box-lid-valve-base* with solvent-based adhesive. Wait for 10 min and remove the masking tape.

36.5 Add the protective vent to the part *box-lid-valve-base* and screw it tight from the bottom with the needle nose pliers (Fig 7.16).

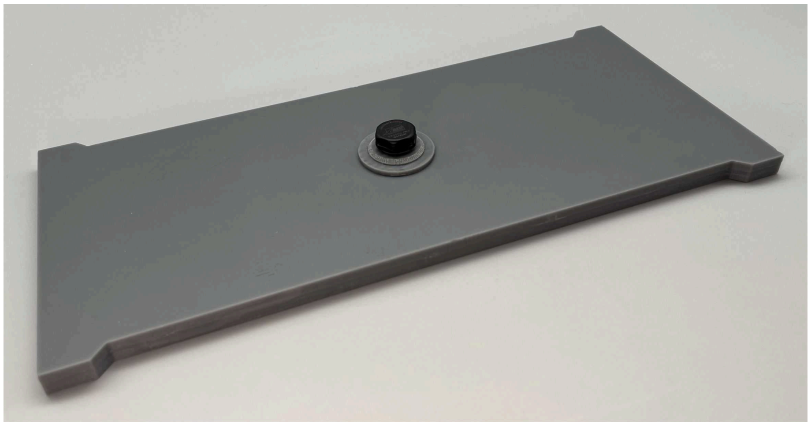

Fig 7.16. Lid with the protective vent attached.

37 **Finish the observation box.**

37.1 Put the lid on top of the box and fixate it with a piece of string around the hooks (Fig 7.17).

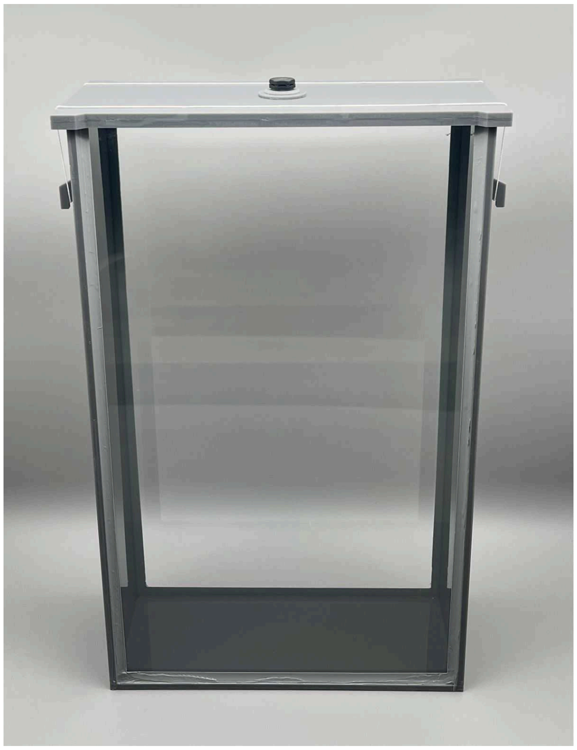

Fig 7.17. Completed observation box with lid.

37.2 Insert the imaging device, guide the two power cables through the openings at the top of the box, and close the lid. Check, if everything fits together (Fig 7.18).

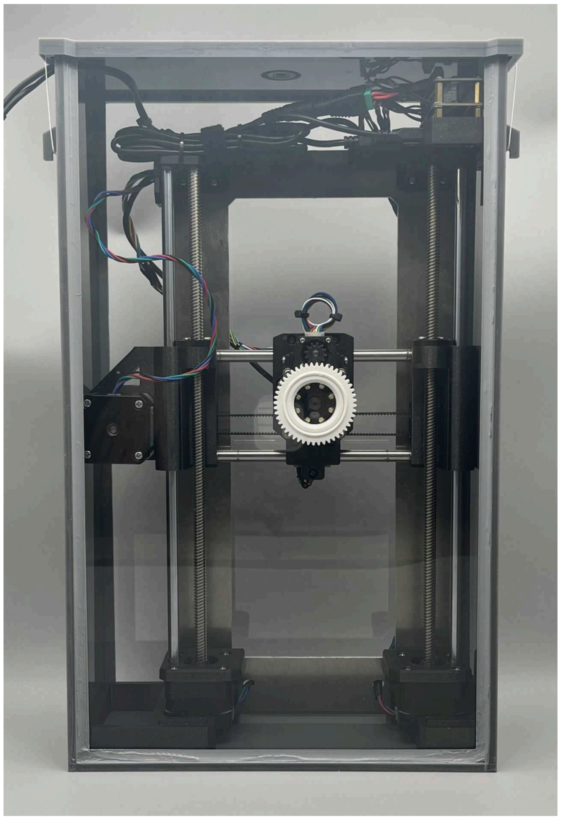

Fig 7.18. Observation box with imaging device inserted.

37.3 A portion of the observation box sticks out of the soil after installation. To limit the exposure of roots and hyphae to ambient light reaching into the observation box, opaque tape or foil is added to both glass windows above the highest position of the DMC (Fig 7.19).

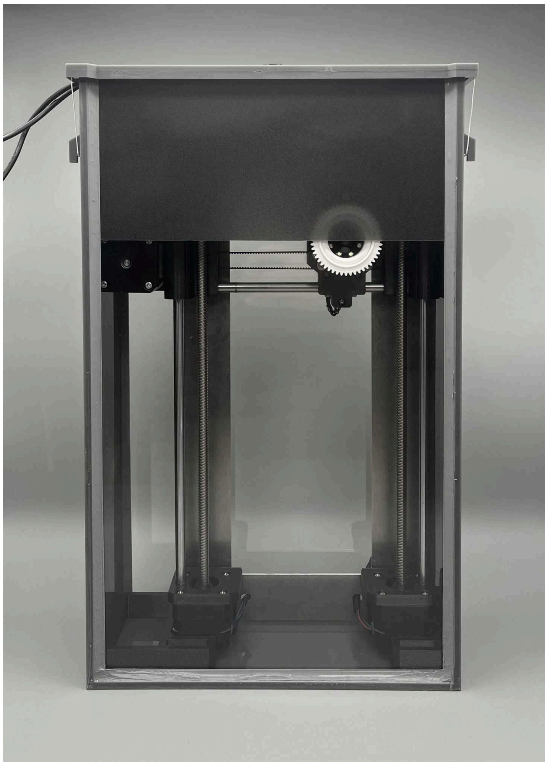

Fig 7.19. Observation box with opaque foil attached.

Section 8: Installation of the software

38 **Gather tools and materials.**

Tools:
Computer
MicroSD card reader
Wi-Fi network with internet access

Materials (quantity):
MicroSD card (1)

> **Note**
>
> Most procedures in this section are already widely documented in online tutorials. This section is just a summary of essential steps. The versions of all software used in this section are included. Compatibility issues may occur with newer versions.

39 **Install the operating system on the control unit of the imaging device.**

39.1 Download, install, and launch [***Raspberry Pi Imager***](#) (Raspberry Pi Foundation; version 1.8.5 used here) on the *Windows* computer.

39.2 Put the microSD card in the microSD card reader and connect it to the *Windows* computer.

39.3 In the user interface of the *Raspberry Pi Imager* (Fig 8.1), click *CHOOSE DEVICE* and select *Raspberry Pi Zero 2 W*. Then, click CHOOSE OS and select the recommended *Raspberry Pi OS* option at the top (Raspberry Pi OS, Legacy, 32-bit, released on 2023/12/05 used here). Finally, click *CHOOSE STORAGE* and select the microSD card inserted earlier.

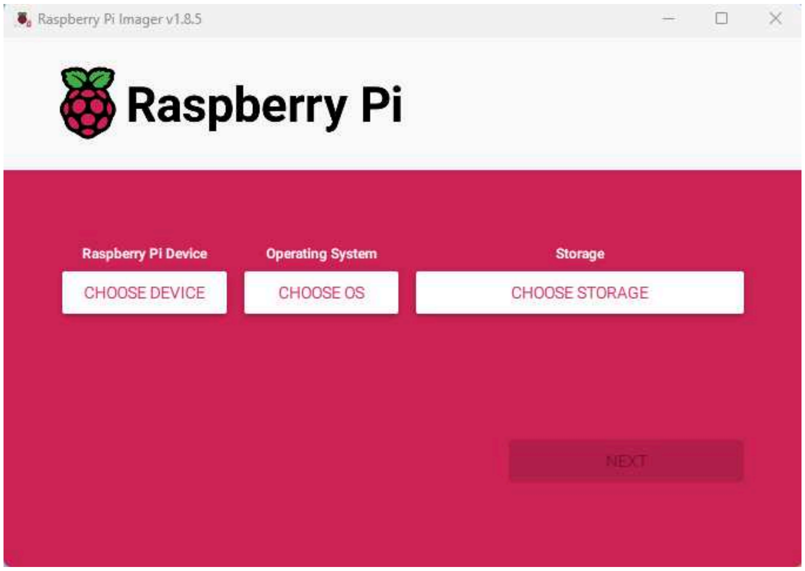

Fig 8.1. User interface of *Raspberry Pi Imager.*

39.4 Click *NEXT* at the bottom right to open the *Advanced options* menu.

39.5 In the *Use OS customisation?* menu that pops up click *EDIT SETTINGS* (Fig. 8.2).

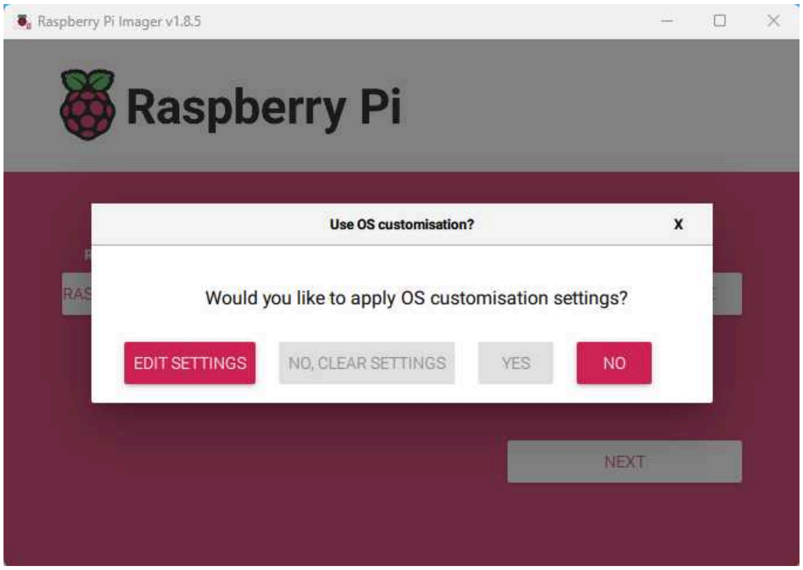

Fig 8.2. *Use OS customisation?* menu.

39.6 In the *OS Customisation* menu (Fig 8.3), check *Set hostname* and do accordingly. Then, check *Set username and password* and do accordingly. The entered information needs to be remembered to connect to the imaging device later on.

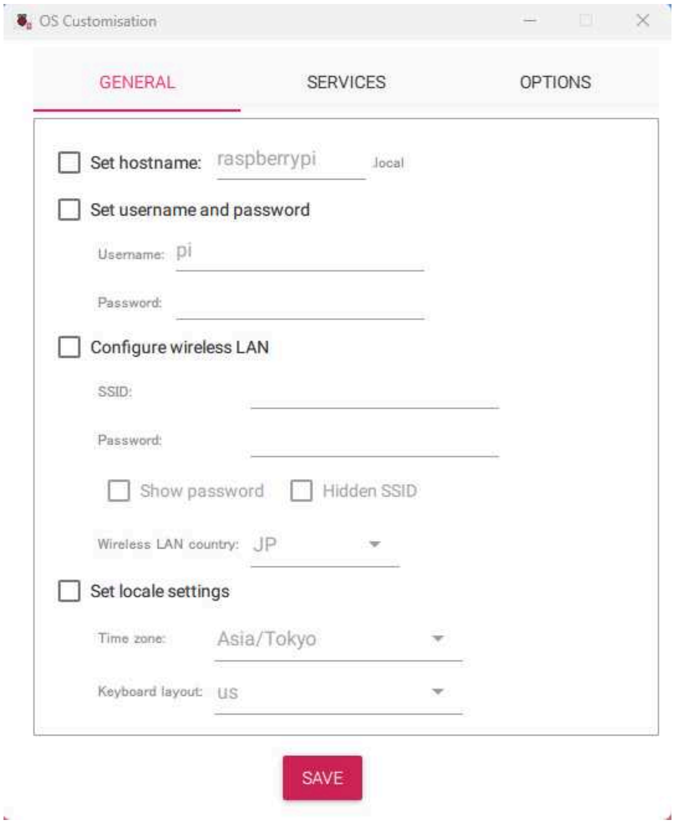

Fig 8.3. *OS Customisation* menu of *Raspberry Pi Imager*.

39.7 Check *Configure wireless LAN*, and set the SSID and password of the Wi-Fi network used communicate with the imaging device.

39.8 Click the *SERVICES* tab, check *Enable SSH*, and select *Use password authentication*.

39.9 Click *SAVE*. Back in the *Use OS customisation?* menu, click *YES*. You will be warned that all existing data on the microSD card will be erased. Click *YES* again to continue.

39.10 Wait until the writing process has finished (Fig 8.4).

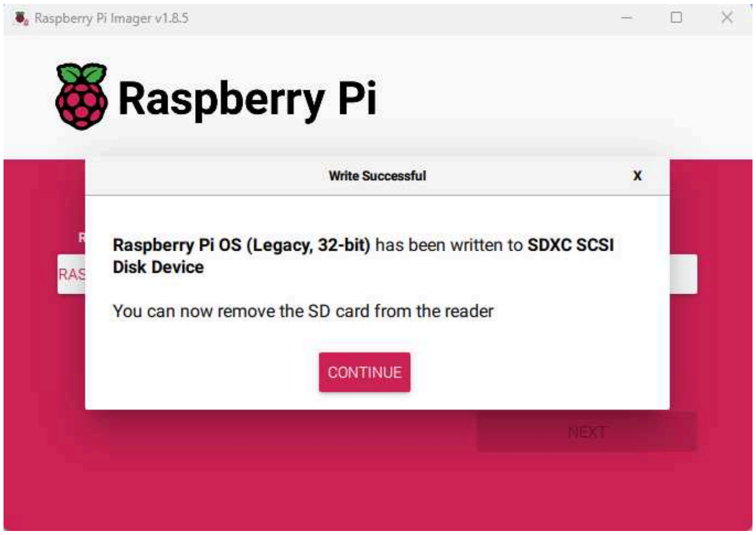

Fig 8.4. Notification that the writing process has finished.

39.11 Eject the microSD card from the card reader and insert it into the control unit. To access its microSD card slot, the control unit has to be pulled up temporarily (Fig 8.5).

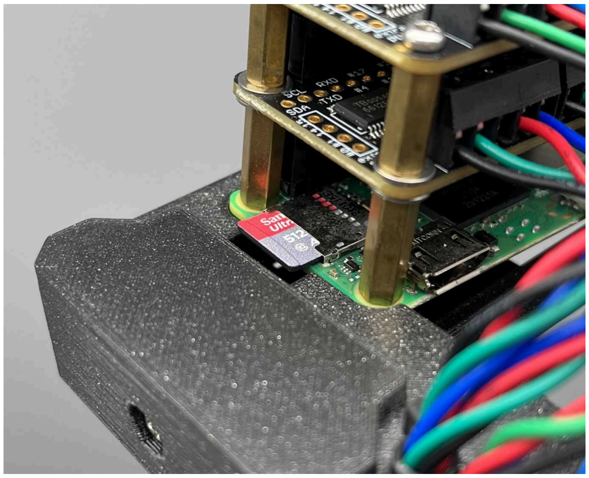

Fig 8.5. Insertion of the microSD card into the control unit.

40 **Connect to the control unit.**

40.1 Plug in both power supplies of the imaging device. The control unit will automatically turn on and start booting.

40.2 Wait about 15 min. When booted for the first time, the filesystem of the *Raspberry Pi OS* is resized. The larger the storage capacity of the microSD card is, the longer the resizing will take. When fully booted the control unit will automatically access the Wi-Fi network using the SSID and password provided earlier.

40.3 Open a *Command Prompt* in *Windows* and type and execute

```
ssh username@hostname.local
```

using the host- and username entered into *Raspberry Pi Imager* before (Fig 8.6).

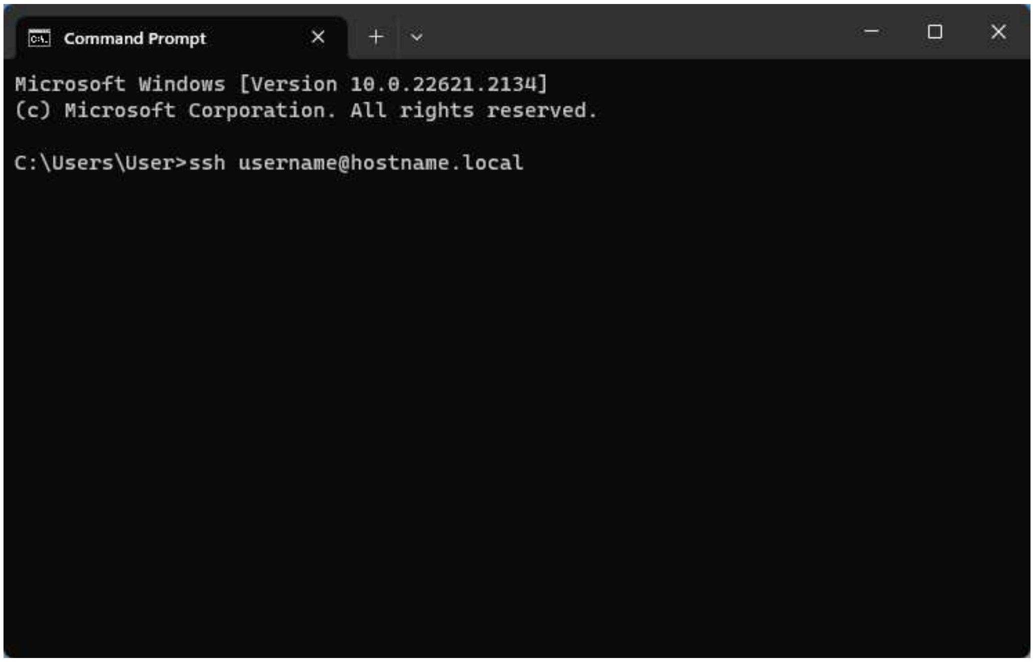

Fig 8.6. *Command Prompt* in *Windows*.

40.4 Entering the password to establish a Secure Shell Protocol (SSH) connection to the control unit.

41 **Enable remote desktop connections to the control unit.**

41.1 In the *Command Prompt*, type and execute.

```
sudo raspi-config
```

41.2 In the *Raspberry Pi Software Configuration Tool*, select *Interface Options* (Fig 8.7).

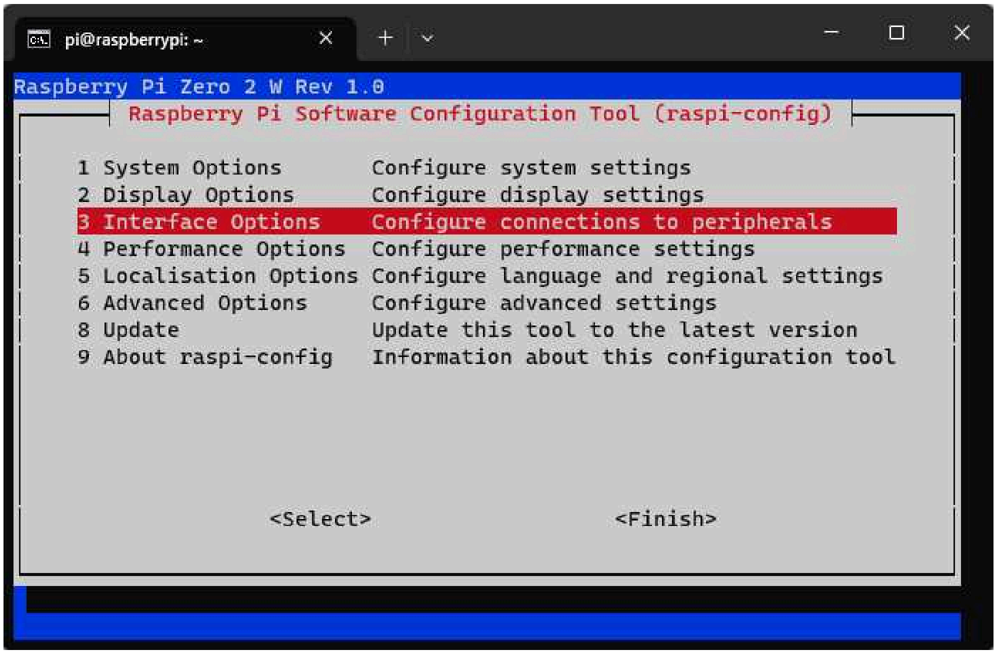

Fig 8.7. User interface of the *Raspberry Pi Software Configuration Tool*.

41.3 Select *VNC* in the new menu (Fig 8.8). When asked *Would you like the VNC Server to be enabled?*, select *Yes*. Select *Ok* right after that.

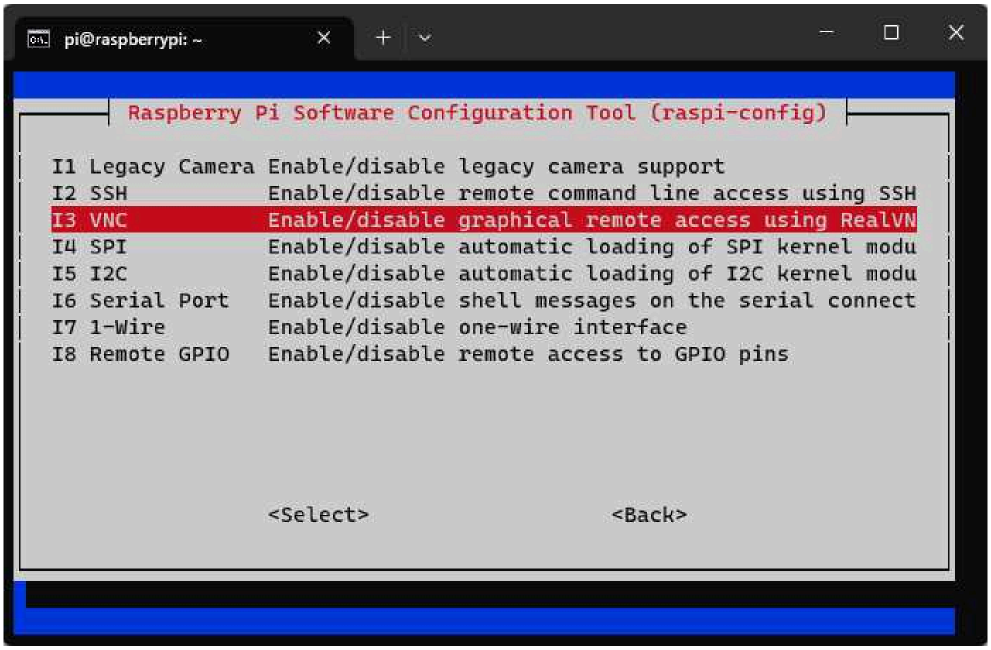

Fig 8.8. *Interface Options* menu of the *Raspberry Pi Software Configuration Tool*.

41.4 Select *Finish* to exit the *Raspberry Pi Software Configuration Tool*.

42 **Open a remote desktop environment.**

42.1 Download, install, and launch ***VNC Viewer*** (RealVNC; version 7.9.0 used here) on the *Windows* computer.

42.2 Select *New Connection...* in the *File* menu.

42.3 Enter the hostname (with or without *.local*) into the *VNC Server* field and press *OK* (Fig 8.9).

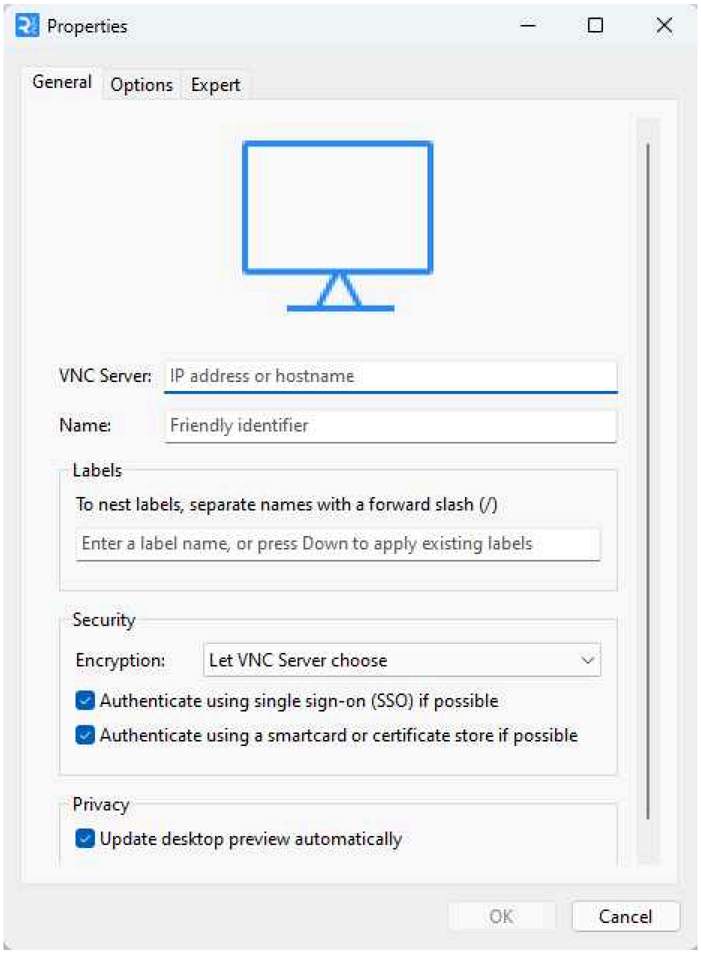

Fig 8.9. *Properties* menu of *VNC Viewer.*

42.4 Double-click on the icon with your hostname in the user interface of *VNC Viewer*. In the *Authentication* menu, enter the username and password and click *OK* to start a remote desktop connection (Fig 8.10).

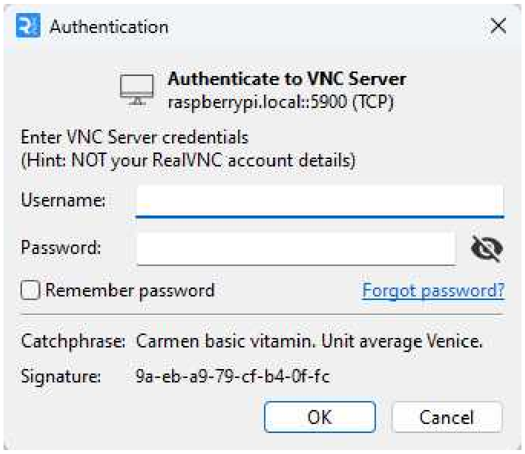

Fig 8.10. *Authentication* menu of *VNC Viewer.*

42.5 If the desktop cannot be shown, reboot the control unit by typing and executing

```
sudo reboot now
```

in the *Command Prompt*.

42.6 Wait for three minutes. The remote desktop connection will be reestablished automatically once rebooted. The remote desktop environment should now be visible. The closed SSH connection does not have to be reestablished for now.

43 **Increase the screen resolution of the remote desktop environment.**

43.1 Click the Raspberry Pi icon in the upper left corner of the remote desktop environment. Then select *Raspberry Pi Configuration* under *Preferences*.

43.2 Click the *Display* tab and change the value of *Headless Resolution* to *1600x1200* (Fig 8.11).

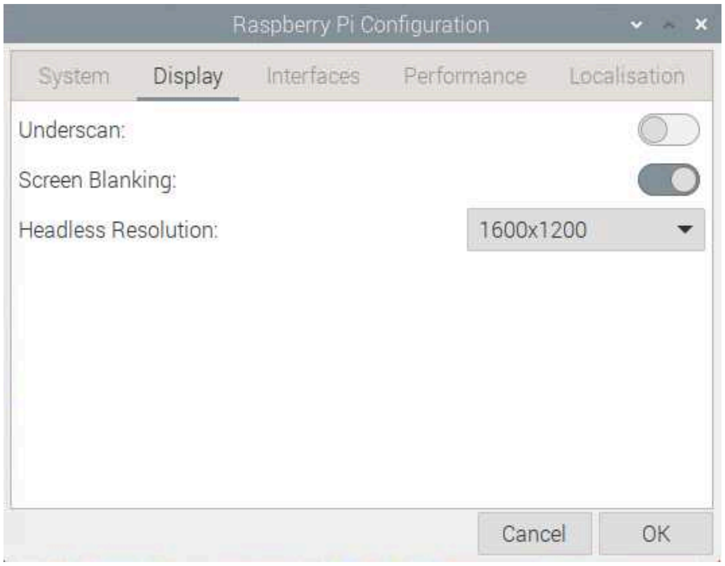

Fig 8.11. *Display* tab of the *Raspberry Pi Configuration* menu.

43.3 Click *OK* and confirm to reboot. The remote desktop connection will be reestablished automatically once rebooted.

43.4 Close *VNC Viewer* and the remote desktop environment.

44 **Set up the *DC & Stepper Motor Bonnets* of the control unit.**

Note

Further details are available in the section ***Installing Software*** of the tutorial *Adafruit DC and Stepper Motor HAT for Raspberry Pi*.

44.1 Reestablish the SSH connection in the *Command Prompt*.

44.2 Type and execute

```
sudo raspi-config
```

to open the *Raspberry Pi Software Configuration Tool*.

44.3 In the configuration tool's menu, select *Interface Options, then I2C* (Fig 8.12). When asked *Would you like the ARM I2C interface to be enabled?*, select *Yes*. Select *Ok* right after that. Finally, select *Finish*.

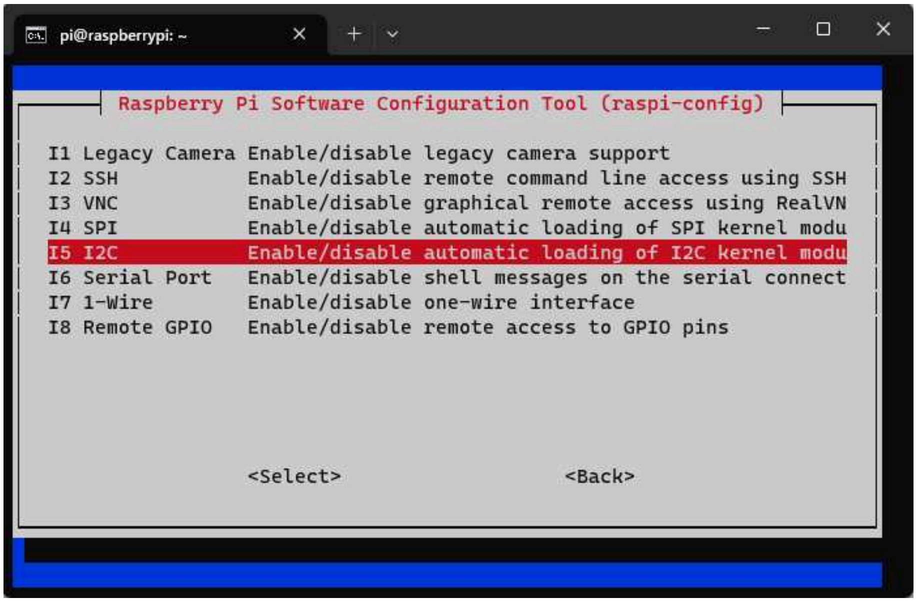

Fig 8.12. *Interface Options* menu with *I2C* highlighted.

44.4 In the *Command Prompt*, paste and execute

```
sudo apt-get update
sudo apt-get upgrade
```

to update the *Raspberry Pi OS*.

44.5 Paste and execute

```
sudo apt install --upgrade python3-pip
sudo apt install --upgrade python3-setuptools
```

to upgrade the *Python* packages ***pip*** and ***setuptools*** (upgraded versions 20.3.4 and 52.0.0 used here, respectively).

44.6 Paste and execute

```
sudo pip install adafruit-python-shell
wget https://raw.githubusercontent.com/adafruit/Raspberry-Pi-Installer-Scripts/master/raspi-blinka.py
sudo python raspi-blinka.py
```

to install the *Python* library ***Adafruit Blinka*** (version 8.32.0 used here). For further details, refer to the instructions in the tutorial ***Installing Blinka on Raspberry Pi***.

44.7 Confirm to reboot the control unit. After rebooting, reestablish the SSH connection.

44.8 Paste and execute

```
sudo pip install adafruit-circuitpython-motorkit
```

to install the *Python* package **Adafruit CircuitPython MotorKit** (version 1.6.14 used here).

45 **Install the *Python* package *OpenCV* on the control unit.**

Note

To make sure the DMC functions properly, the right release and version of the *Python* package *OpenCV* must be installed. It is, hence, recommended to install *OpenCV* as shown below.

45.1 Type and execute

```
cat /etc/os-release
```

to display the *Raspberry Pi OS* version (Fig 8.13)

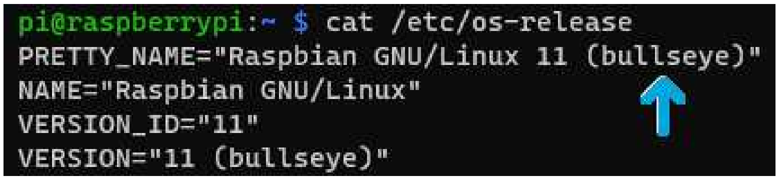

Fig 8.13. Version of the *Raspberry Pi OS.*

45.2 Type and execute

```
cat /proc/cpuinfo
```

to display the CPU version (Fig 8.14).

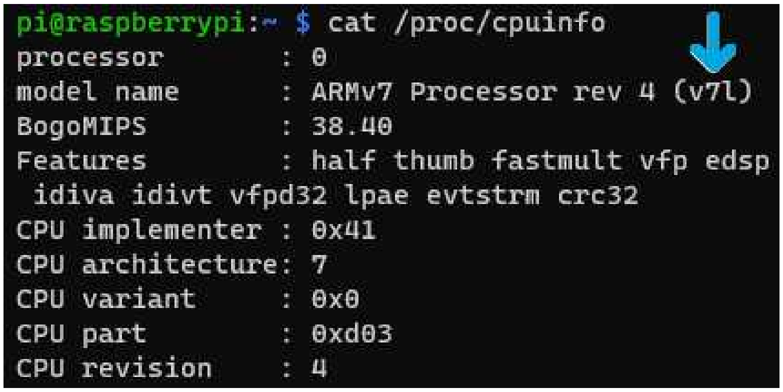

Fig 8.14. Version of the CPU.

45.3 Type and execute

```
python --version
```

to display the *Python* version (Fig 8.15).

```
pi@raspberrypi:~ $ python --version
Python 3.9.2
```

Fig 8.15. Version of *Python.*

45.4 Go to the **[*project page of OpenCV*]** in the package repository *piwheels*.

45.5 Look up the latest release of *OpenCV* that was built successfully under the OS and *Python* version displayed earlier (Fig 8.16).

Releases

Version	Released	Buster Python 3.7	Bullseye Python 3.9	Bookworm Python 3.11	Files
4.9.0.80	2023-12-31	✗	✗	✗	
4.8.1.78	2023-09-28	✗	✗	✗	
4.8.0.76	2023-08-09	✗	✗	✗	
4.8.0.74	2023-06-30	✗	✗	✗	
4.7.0.72	2023-02-22	✓	✗	✓	+
4.7.0.68	2022-12-30	✓	✗	✗	+
4.6.0.66	2022-06-08	✓	✓	✗	+
4.5.5.64	2022-03-09	✓	✓	✗	+
4.5.5.62	2021-12-29	✓	✓	✗	+
4.5.4.60	2021-11-22	✓	✓	✗	+

Fig 8.16. Latest releases of *OpenCV.*

45.6 Click on the *How to install this version* button next to the link with the correct *Python* and CPU version (Fig 8.17). The *Python* version is the number after *cp*: *Python* version 3.9 corresponds to *cp39*. The CPU version is shown after *arm*.

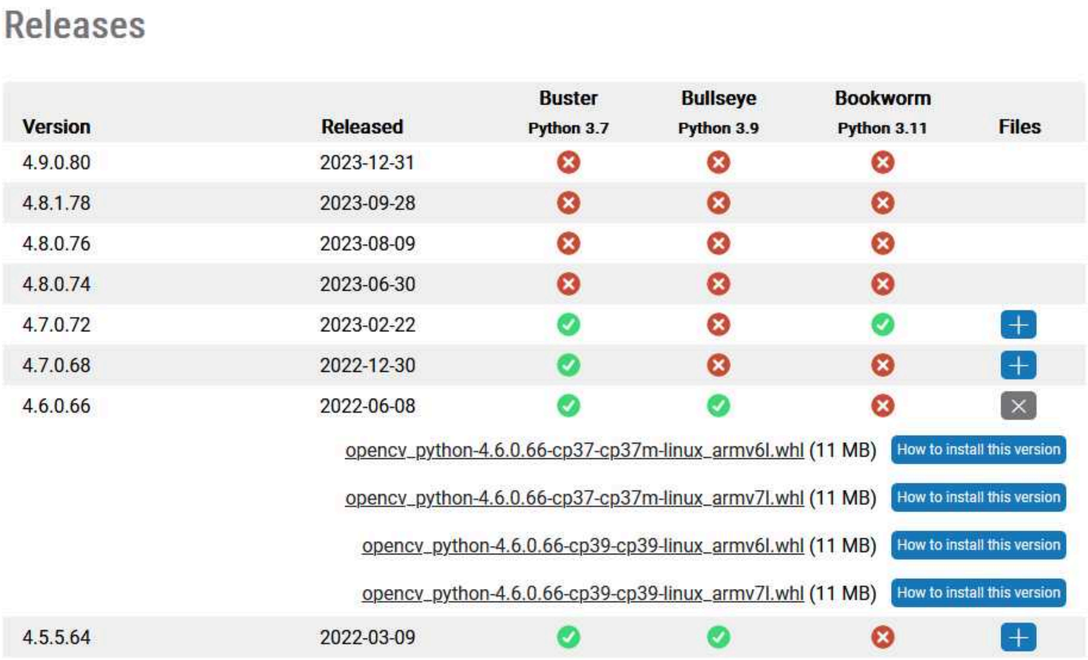

Releases

Version	Released	Buster Python 3.7	Bullseye Python 3.9	Bookworm Python 3.11	Files
4.9.0.80	2023-12-31	✗	✗	✗	
4.8.1.78	2023-09-28	✗	✗	✗	
4.8.0.76	2023-08-09	✗	✗	✗	
4.8.0.74	2023-06-30	✗	✗	✗	
4.7.0.72	2023-02-22	✓	✗	✓	+
4.7.0.68	2022-12-30	✓	✗	✗	+
4.6.0.66	2022-06-08	✓	✓	✗	×
	opencv_python-4.6.0.66-cp37-cp37m-linux_armv6l.whl (11 MB)				How to install this version
	opencv_python-4.6.0.66-cp37-cp37m-linux_armv7l.whl (11 MB)				How to install this version
	opencv_python-4.6.0.66-cp39-cp39-linux_armv6l.whl (11 MB)				How to install this version
	opencv_python-4.6.0.66-cp39-cp39-linux_armv7l.whl (11 MB)				How to install this version
4.5.5.64	2022-03-09	✓	✓	✗	+

Fig 8.17. Versions of the selected *OpenCV* release.

45.7 Copy the code from the yellow box (Fig 8.18) into the *Command Prompt* and execute to install *OpenCV* (version 4.6.0.66 used here).

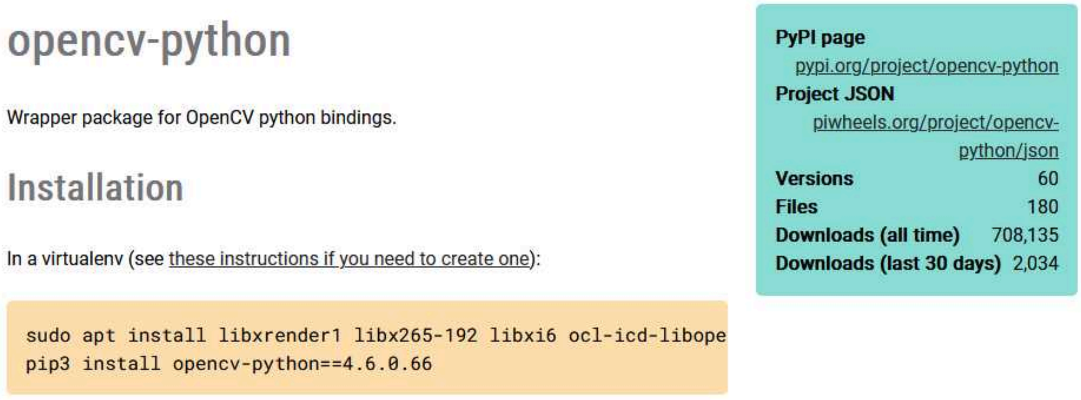

Fig 8.18. Code to install the selected release and version of *OpenCV.*

45.8 Paste and execute

```
sudo echo "" >> ~/.bashrc
sudo echo "# Export display to run OpenCV through SSH" >>
~/.bashrc
sudo echo "export DISPLAY=:0.0" >> ~/.bashrc
```

to enable access to the graphical output of *OpenCV* over the SSH connection.

46 **Install other dependencies of the soil imaging script on the control unit.**

46.1 Open the **project page of the *Python* package *pandas*** in the *piwheels* repository and install the newest compatible version of ***pandas*** (version 2.2.0 used here) in the same way as for *OpenCV*.

46.2 Open the **project page of the *Python* package *numpy*** in the *piwheels* repository and install the newest compatible version of ***numpy*** (version 1.26.4 used here) in the same way as for *OpenCV*.

> Note
>
> *numpy* is already installed on the system but must be reinstalled here using the *piwheels* repository for proper functioning of the imaging device.

46.3 Type and execute

```
sudo apt install screen
```

to install ***screen*** (version 4.8.0 used here). The package enables automated imaging without being connected to the imaging device.

46.4 Paste and execute

```
wget https://github.com/h-
schaefer/hyphascope/raw/main/soil_imager_kit.py
```

to download the *Python* functions needed to control the imaging device (version 0.1.0-alpha used here).

46.5 Type and execute

```
sudo halt
```

to turn off the imaging device until operation.

Section 9: Application of the device

47 **Gather tools and materials.**

Tools:

Computer

MicroSD card reader

Wi-Fi network with internet access

Spade

Measure

Spirit level
Sturdy plastic bags

48 **Install the imaging device in the target soil.**

Note

For natural soils with distinct layers, the soil excavated during the device installation may be collected separately for each layer and put back in order when filling the gaps around the observation box. Furthermore, roots and rocks may be removed from the excavated soil with a sieve or by manual picking.

48.1 Using the spade and the measure, dig a hole that is 30 cm long, 20 cm wide, and 31 cm deep in the target soil. Collect the removed litter and excavated soil in sturdy plastic bags.

48.2 Place the observation box in the center of the hole (Fig 9.1). Make sure the observation box is leveled. The lower edge of the opaque foil on each of the box's windows should be at ground surface height.

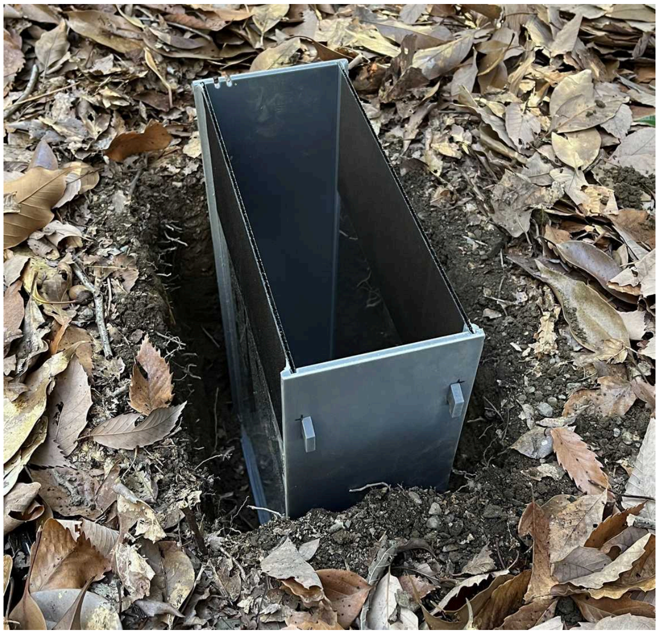

Fig 9.1. Observation box placed in the center of the hole.

48.3 Fill the gaps to either side with excavated soil. Then, place the imaging device into the observation box (Fig 9.2). Make sure that the DMC is in the top corner below the control unit.

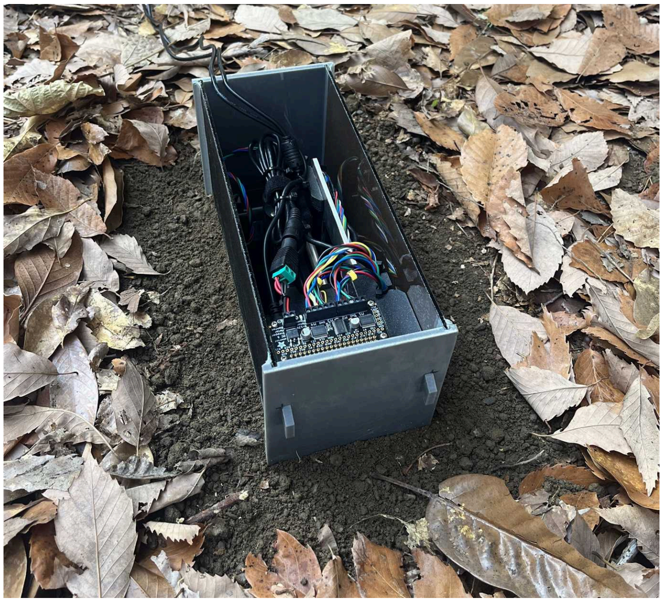

Fig 9.2. Installed observation box with imaging device inserted.

48.4 Close the lid and secure it with string. Add some of the collected litter back (Fig 9.3).

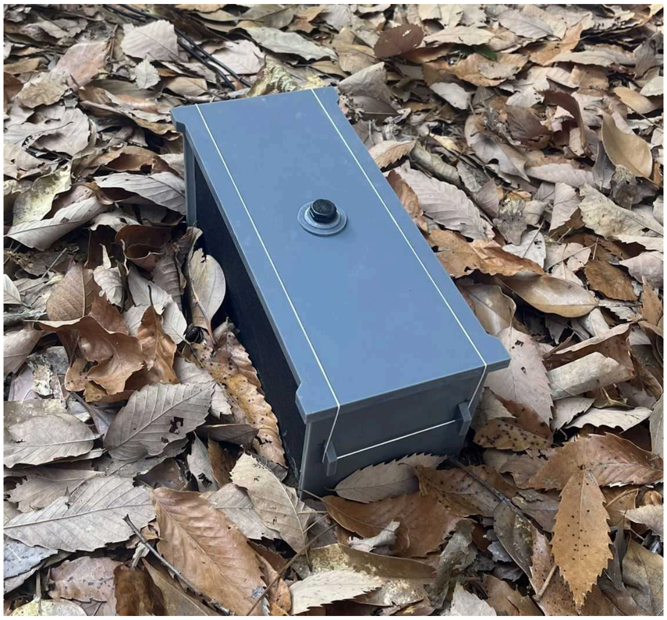

Fig 9.3. Installed soil imaging device

48.5 Connect the two power adapters of the imaging device to a power outlet. Ideally, there is a power switch to easily turn the imaging device on and off. In the field, additional equipment is needed to protect the cables and power supplies from rain and wildlife.

49 **For small soil volumes: Attach a soil container to the front and/or back of the observation box (Fig 9.4).**

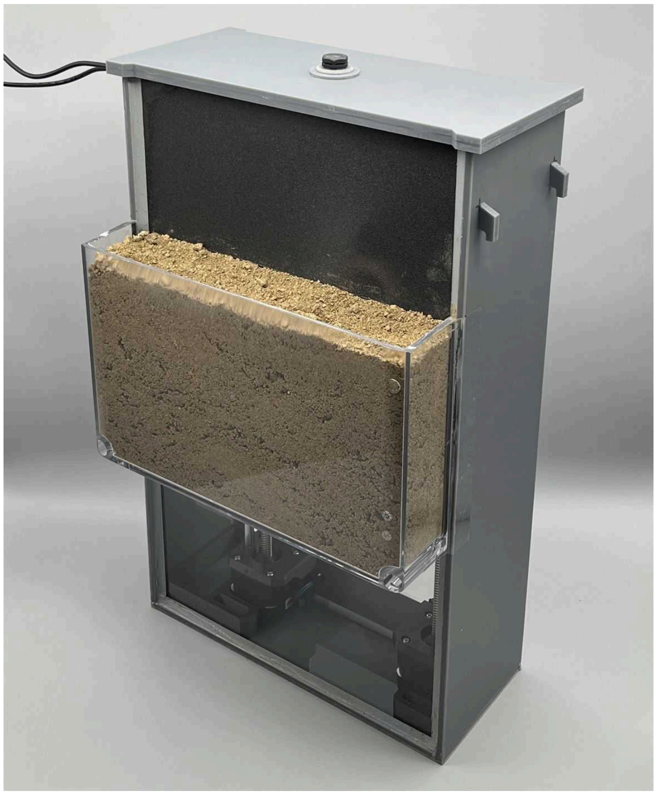

Fig 9.4. Soil container attached to the imaging device.

Note

The container should be fixated to the acrylic box walls rather than the glass sheet itself. Furthermore, the side of the container that faces the observation box should be open, so that the soil sits right against the glass sheet of the observation box. To limit the exposure of roots and hyphae to ambient light, opaque tape or foil should be added to any exposed transparent surface.

50 **Compile an imaging session.**

50.1 Download, install, and launch ***Visual Studio Code*** (version 1.86.1 used here) on the *Windows* computer.

50.2 Download the file *imaging_session.py* from the **GitHub repository for the imaging device**.

50.3 Open the file *imaging_session.py* in *Visual Studio Code* and edit it according to the instructions in the **GitHub repository for the imaging device** (Fig 9.5).

Usage

- Step 1. Open the file *imaging_session.py* in a code editor.
- Step 2. Compose an imaging session from the five functions below:
  - *Imager.move_dmc()*: Move the DMC to a specified position along three axes.
  - *Imager.set_dmc()*: Set the image size and type to be stored.
  - *Imager.adjust_focus()*: Manually adjust the focus of the DMC.
  - *Imager.image_soil()*: Perform the automated imaging of the soil profile within a given volume.
  - *Imager.end_session()*: End the imaging session.
- Step 3. Save and close the file *imaging_session.py*.
- Step 4. Transfer the file to the imaging device and carry out the imaging session following the section *Application of the device* in the protocol on protocols.io.
- Step 5. Transfer the soil profile images to the host computer and process them following the same section.

Example

```
# Initialize the soil imager
imager1 = soil_imager_kit.Imager()
# Move the DMC to position 1 mm on the X axis and 0.5 mm on the Z axis
```

Fig 9.5. Instructions for imaging in the *GitHub* repository.

Note

If the imaging device is used for the first time or a problem occurred in the previous imaging session, conduct the manual focus adjustment (function *adjust_focus*) before the automated imaging, as shown in the example code. Minor focus adjustments are done automatically during an imaging session.

50.4 Save and close the file.

51 **Carry out the soil imaging session.**

51.1 Turn on the imaging device.

51.2 Download, install, and launch ***FileZilla*** (version 3.66.5 used here) on the *Windows* computer.

51.3 In the *Site Manager* menu under the *File* tab, add the imaging device as a new site and connect to it (Fig 9.6).

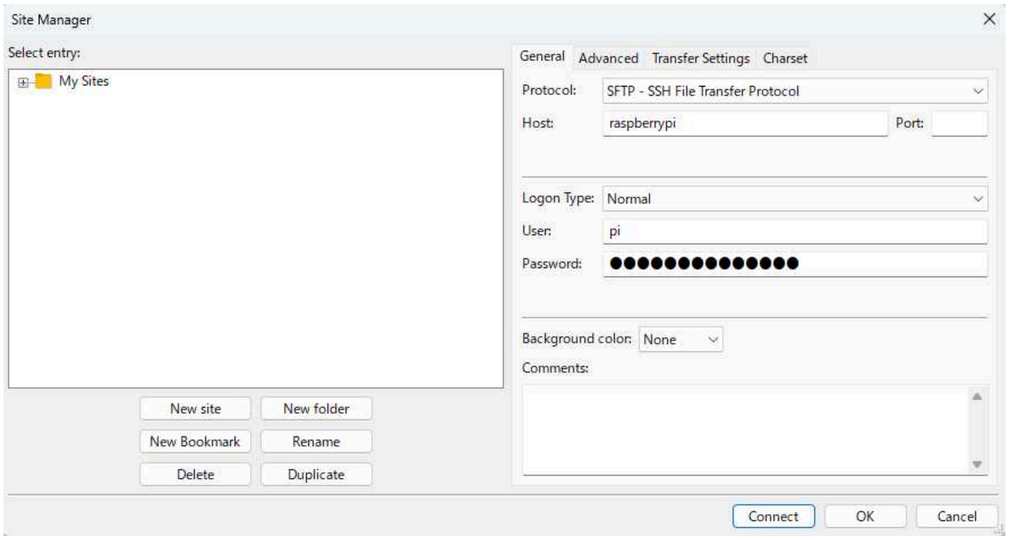

Fig 9.6. *Site Manager* menu of *FileZilla.*

51.4 Copy the edited file *imaging_session.py* from the *Windows* computer to *home/username/* on the imaging device (Fig 9.7).

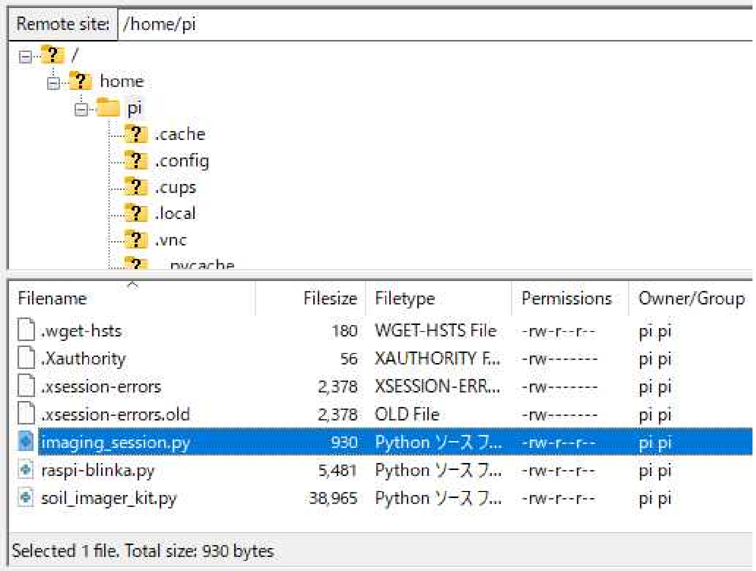

Fig 9.7. File *imaging_session.py* copied to the imaging device.

51.5 Open a *Command Prompt* on the *Windows* computer, and type and execute

```
ssh username@hostname.local
```

establish an SSH connection to the imaging device.

51.6 Type and execute

```
screen
```

to open a *screen* window. Press *Space* or *Return* to end the information screen.

51.7 In the *screen* window, type and execute

```
python imaging_session.py
```

to run the imaging session script.

51.8 If the imaging session starts with a manual focus adjustment, follow the instructions in the *Command Prompt* (Fig 9.8). Otherwise, the imaging of the soil profile starts automatically.

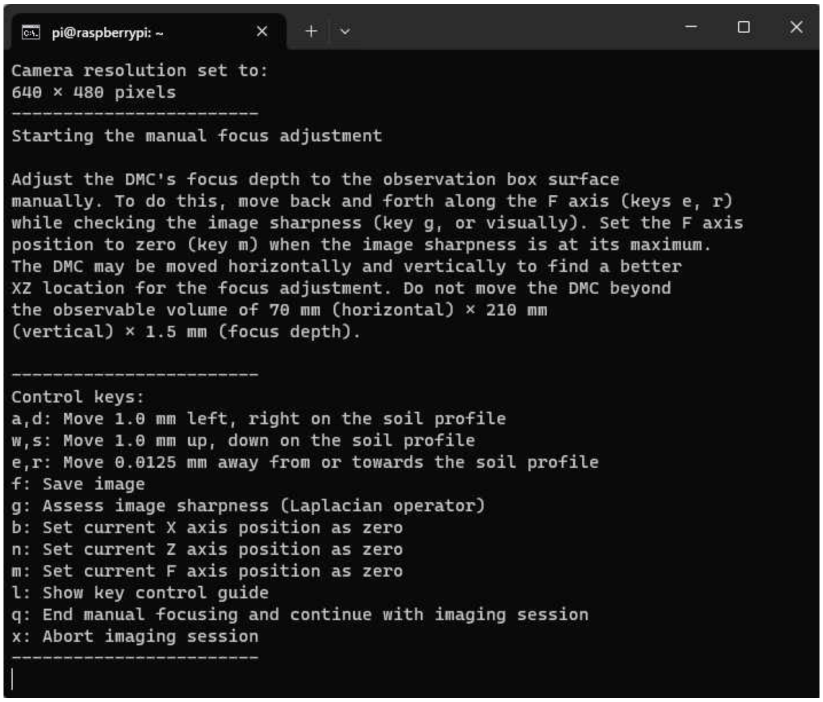

Fig 9.8. Instructions for the manual focus adjustment.

51.9 Press ***Ctrl* + *a*** and then ***d*** to return to the main window of the *Command Prompt*.

51.10 You may now close the *Command Prompt*. The imaging process will continue to run.

51.11 After the imaging session has ended, the control unit of the imaging device can be turned off by typing

```
sudo halt
```

in the *Command Prompt*. The DMC's LED lights will stay on until the imaging device is powered off.

52 **Check on the progress during a soil imaging session.**

52.1 *(option 1)* In a Command Prompt with an SSH connection to the imaging device, type and execute

```
screen -r
```

to enter the *screen* window that runs the imaging session script (Fig 9.9). Exit the window by pressing ***Ctrl*** + ***a*** and then ***d***.

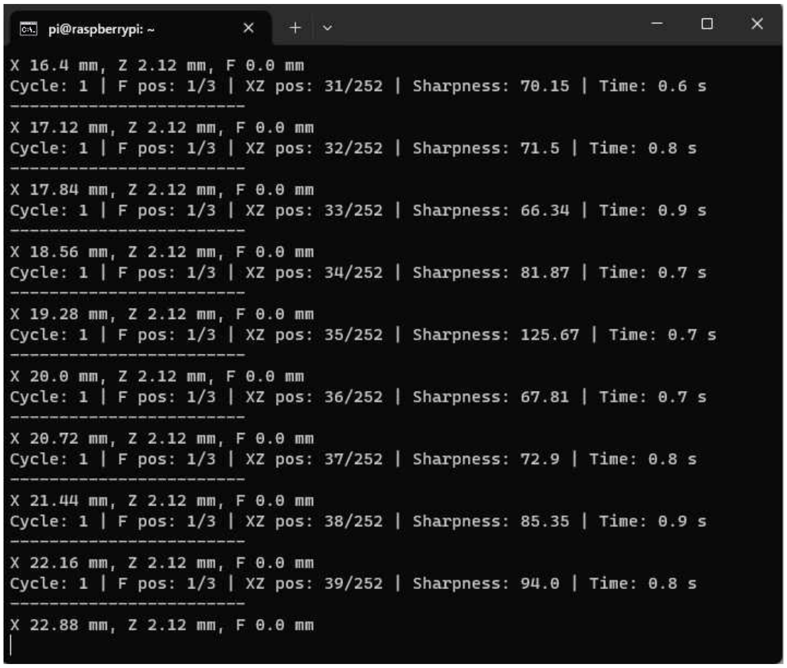

Fig 9.9. Progress of the automated soil imaging shown in the *Command Prompt*.

52.2 *(option 2)* Use *FileZilla* to locate the log file (*log_YYYYMMDDhhmmss.csv*) in the session folder *home/username/session_YYYYMMDDhhmmss*, copy it to the *Windows* computer, and inspect it in a program that can read comma-separated values (CSV) files (Fig 9.10).

	A	B	C	D	E	F
1	datetime	cycle_no	f_depth	xz_pos_no	sharp	
2	2024/3/12 8:23	1	0	1	93.22	
3	2024/3/12 8:23	1	0	2	108.76	
4	2024/3/12 8:23	1	0	3	106.41	
5	2024/3/12 8:23	1	0	4	68.31	
6	2024/3/12 8:23	1	0	5	56.97	
7	2024/3/12 8:23	1	0	6	96.95	
8	2024/3/12 8:23	1	0	7	99.57	
9	2024/3/12 8:23	1	0	8	105.36	
10	2024/3/12 8:23	1	0	9	99.3	
11	2024/3/12 8:23	1	0	10	81.43	
12	2024/3/12 8:23	1	0	11	73.89	
13	2024/3/12 8:23	1	0	12	76.95	

Fig 9.10. Contents of the session log file.

53 **Retrieve the images from the imaging device.**

53.1 *(option 1)* Use *FileZilla* to locate the session folder *home/username/session_YYYYMMDDhhmmss* on the imaging device and copy it to the *Windows* computer.

53.2 *(option 2)* Download, install, and launch ***Linux Reader*** (DiskInternals; version 4.19.2 used here).

53.3 *(option 2)* Remove the microSD card from the (turned off) imaging device and insert it into the microSD card reader of the *Windows* computer.

53.4 *(option 2)* Open the *rootfs* partition of the microSD card in *Linux Reader* and locate the session folder *home/username/session_YYYYMMDDhhmmss* (Fig 9.11).

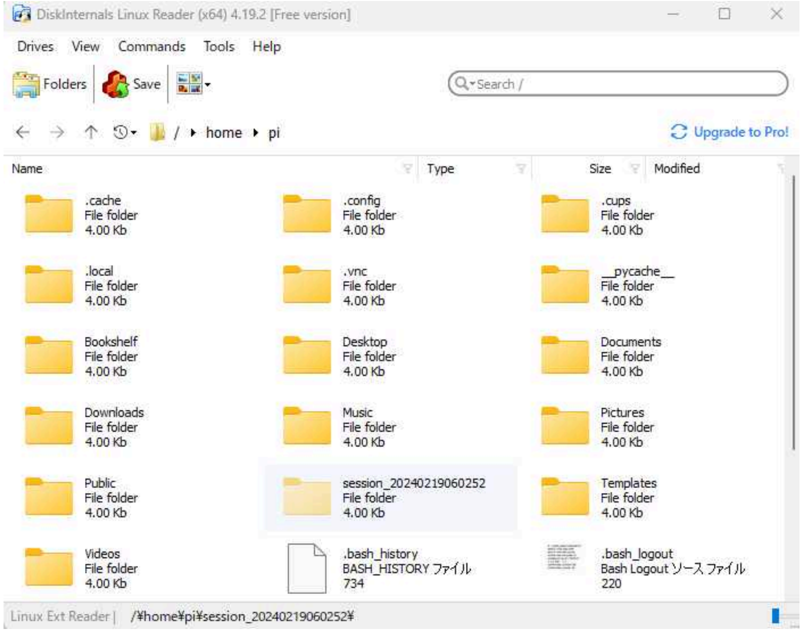

Fig 9.11. Session folder accessed through *Linux Reader*.

53.5 *(option 2)* Mark the image folder and click *Save*. Then follow the *Export Wizard* menu.

54 **Register the retrieved images into a large continuous soil image.**

54.1 Download, extract, launch, and update the ***Fiji* distribution of *imageJ*** (version 1.54f used here).

54.2 Select the *Grid/Collection stitching* plugin (**Preibisch et al. 2009**; version 1.2 used here) in the *Stitching* menu of the *Plugins* tab.

54.3 Select the type *Grid: snake by rows* and the order *Right & Down* and confirm by clicking *OK* (Fig 9.12).

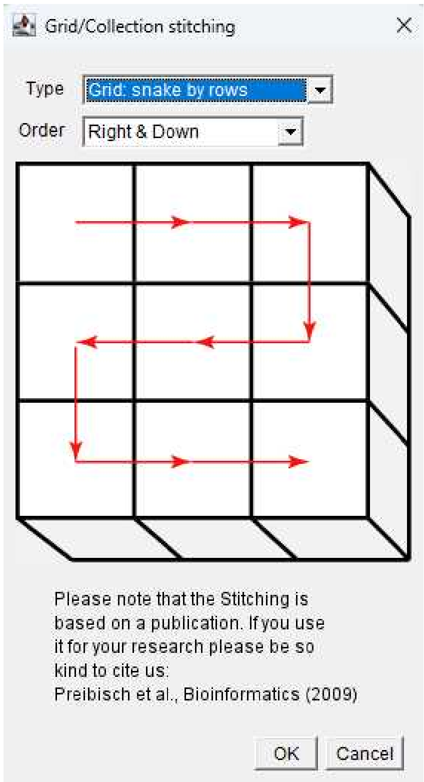

Fig 9.12. First menu of the *Grid/Collection stitching* plugin.

54.4 In the second menu, select the directory of the images to be registered (Fig 9.13). Since images are registered separately for each imaging cycle and focus depth, a subdirectory of the session folder has to be selected.

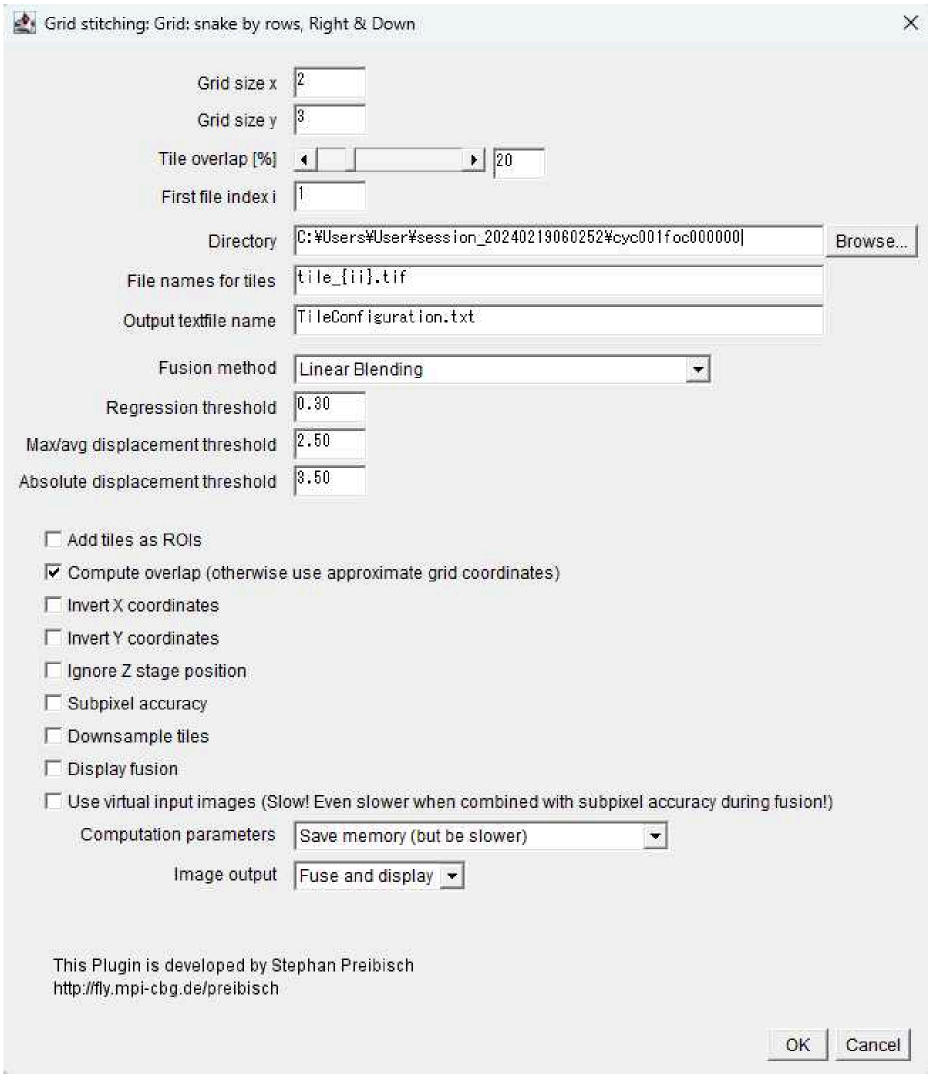

Fig 9.13. Second menu of the *Grid/Collection stitching* plugin.

54.5 For *Grid size x*, enter the value under *n_imgs_x* for the selected imaging cycle in the cycle info file (*cycles_YYYYMMDDhhmmss.csv*) within the session folder. For *Grid size y*, enter the value under *n_imgs_z*.

54.6 For *Tile overlap [%]*, enter 12.

54.7 For *File names for tiles*, enter *tile_{iiiii}.filetype*, e.g. *tile_{iiiii}.jpg*.

54.8 Click *OK* to start the image registration.

54.9 To combine the color channels of the registered image, select *Stack to RGB* in the *Color* menu of the *Image* tab.

54.10 Click *Save* in the *File* tab to save the completed image (Fig 9.14).

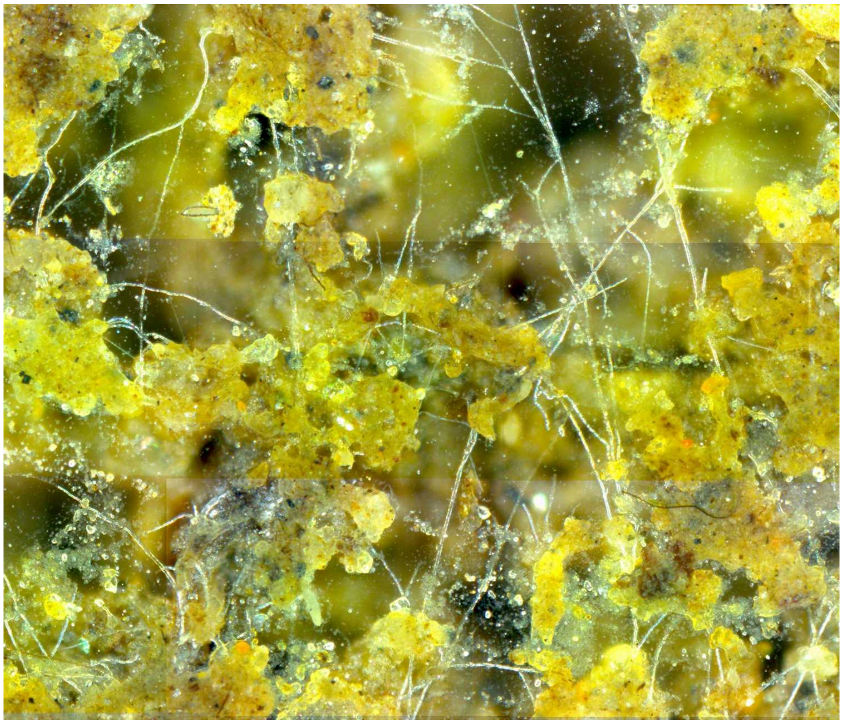

Fig 9.14. Soil profile image assembled from 3 x 3 individual DMC images.

Protocol references

Prusa Research. **Original Prusa i3 MK3S+ kit assembly v3.26**. URL accessed: 2024/03/14.
Adafruit, Rembor K. **Adafruit DC and Stepper Motor HAT for Raspberry Pi**. URL accessed: 2024/03/14.
Preibisch S, Saalfeld S, Tomancak P. **Globally optimal stitching of tiled 3D microscopic image acquisitions**. Bioinformatics. 2009;25: 1463–1465.
