# Supplementary figures and images for: Assembly and application of a low-cost high-resolution imaging device for hyphae in soil
Source: PLoS One. 2025 Jan 24;20(1):e0318083. doi: 10.1371/journal.pone.0318083 (PMC11760638; doi:10.1371/journal.pone.0318083)

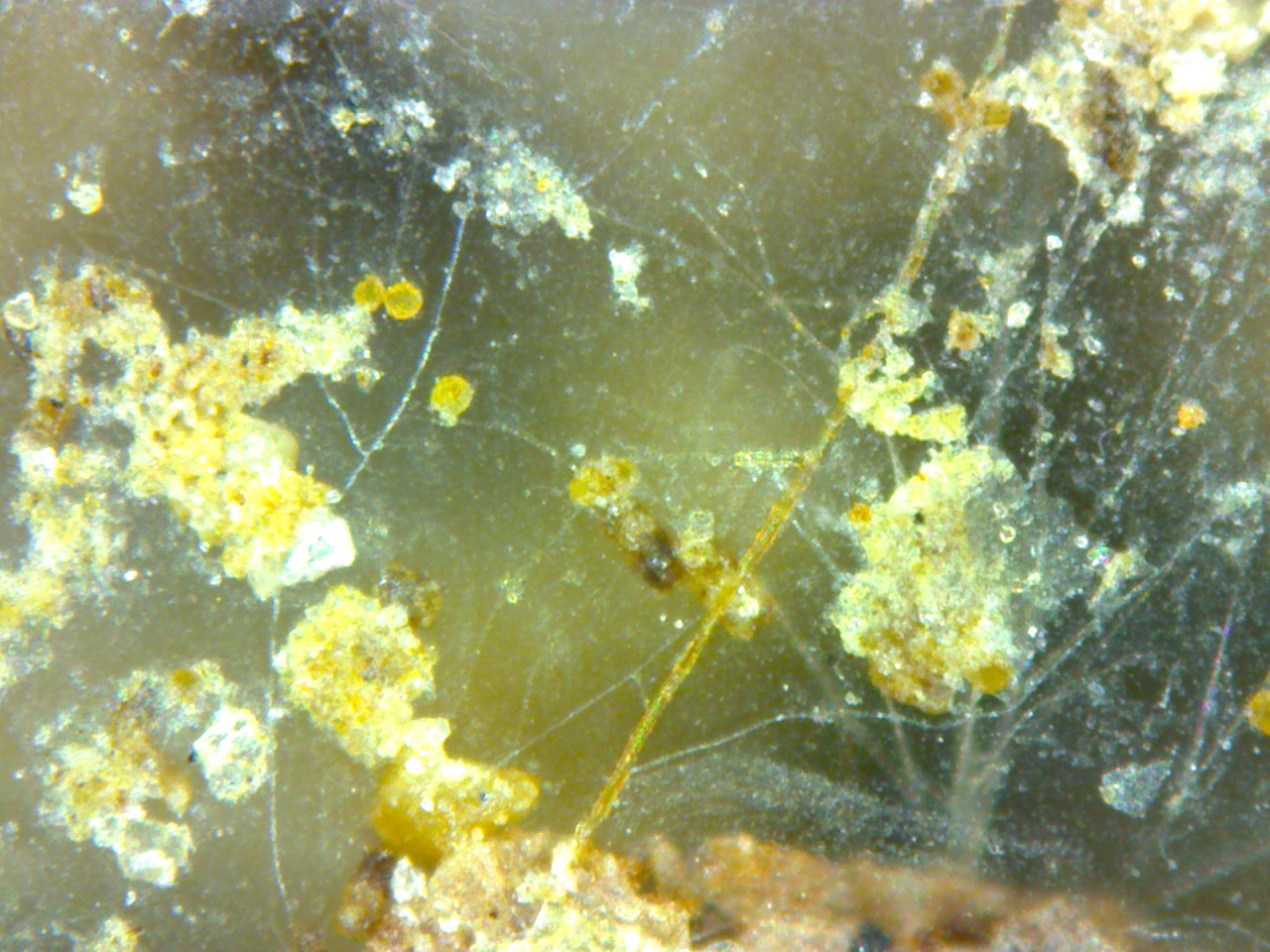

Supplement: S1 Dataset — Original images in the JPG format taken at an imaging resolution of 0.65 μm px-1 (39200 dpi). (ZIP) [file pone.0318083.s002.zip › image_foc00000.jpg]

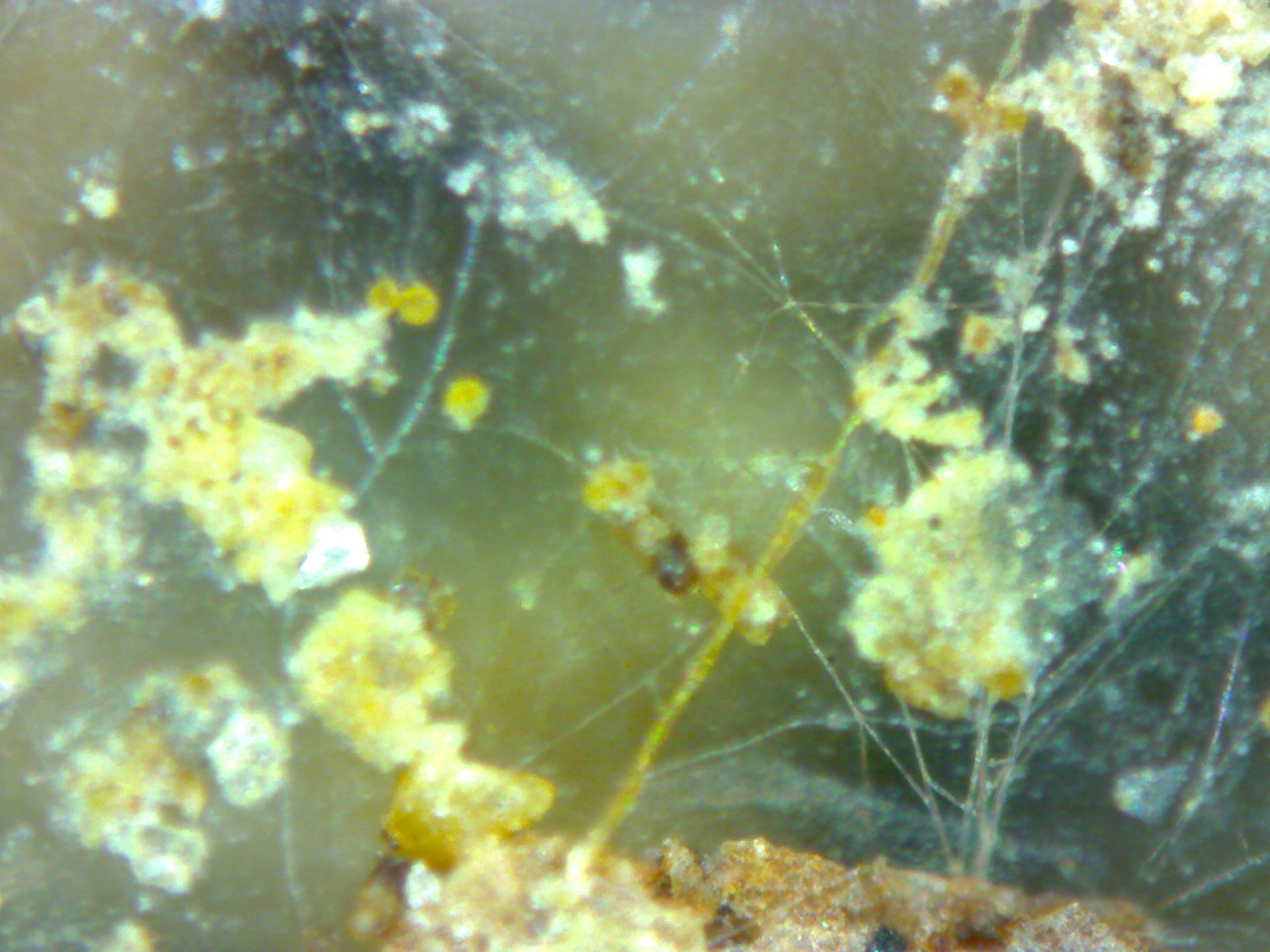

Supplement: S1 Dataset — Original images in the JPG format taken at an imaging resolution of 0.65 μm px-1 (39200 dpi). (ZIP) [file pone.0318083.s002.zip › image_foc00025.jpg]

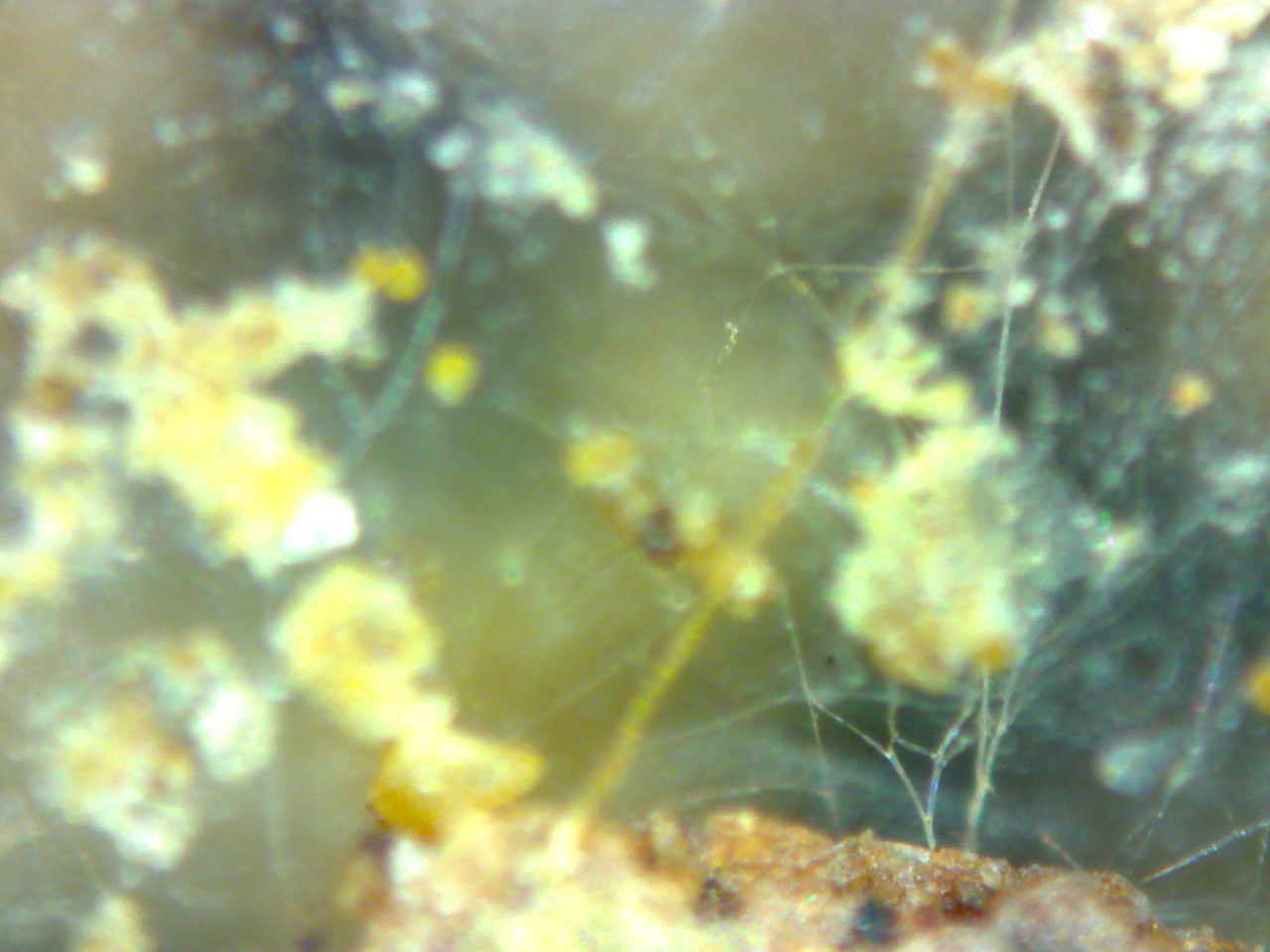

Supplement: S1 Dataset — Original images in the JPG format taken at an imaging resolution of 0.65 μm px-1 (39200 dpi). (ZIP) [file pone.0318083.s002.zip › image_foc00050.jpg]
